# Functional antagonism between CagA and DLC1 in gastric cancer

**DOI:** 10.1038/s41420-022-01134-x

**Published:** 2022-08-13

**Authors:** Isabel Hinsenkamp, Jan P. Köhler, Christoph Flächsenhaar, Ivana Hitkova, Sabine Eberhart Meessen, Timo Gaiser, Thomas Wieland, Christel Weiss, Christoph Röcken, Michael Mowat, Michael Quante, Karin Taxauer, Raquel Mejias-Luque, Markus Gerhard, Roger Vogelmann, Nadja Meindl-Beinker, Matthias Ebert, Elke Burgermeister

**Affiliations:** 1 Department of Medicine II, Medical Faculty Mannheim, Heidelberg University, Mannheim, Germany; 2 Institute of Pathology, Medical Faculty Mannheim, Heidelberg University, Mannheim, Germany; 3 Experimental Pharmacology, European Center for Angioscience (ECAS), Medical Faculty Mannheim, Heidelberg University, Mannheim, Germany; 4 Department of Medical Statistics and Biomathematics, Medical Faculty Mannheim, Heidelberg University, Mannheim, Germany; 5 Institute of Pathology, Christian Albrechts University Kiel, Kiel, Germany; 6 CancerCare Manitoba Research Institute, Department of Biochemistry & Medical Genetics, University of Manitoba, Winnipeg, MB Canada; 7 Department of Medicine II, Gastroenterology, Hepatology, Endocrinology, and Infectious Diseases, Faculty of Medicine, University Medical Center Freiburg, Freiburg, Germany; 8 Institute for Med. Microbiology, Immunology and Hygiene, School of Medicine, Technical University of Munich (TUM), Munich, Germany; 9 DKFZ-Hector Institute at the University Medical Center, Mannheim, Germany; 10 Mannheim Institute for Innate Immunoscience (MI3), Medical Faculty Mannheim, Heidelberg University, Mannheim, Germany; 11 Clinical Cooperation Unit Healthy Metabolism, Center of Preventive Medicine and Digital Health, Medical Faculty Mannheim, Heidelberg University, Mannheim, Germany; 12 Mannheim Cancer Center (MCC), Medical Faculty Mannheim, Heidelberg University, Mannheim, Germany

**Keywords:** Cancer stem cells, Infection

## Abstract

*Helicobacter* (H.) *pylori*-induced gastritis is a risk factor for gastric cancer (GC). Deleted-in-liver-cancer-1 (DLC1/ARHGAP7) inhibits RHOA, a downstream mediator of virulence factor cytotoxin-A (CagA) signalling and driver of consensus-molecular-subtype-2 diffuse GC. DLC1 located to enterochromaffin-like and MIST1+ stem/chief cells in the stomach. DLC1+ cells were reduced in *H. pylori* gastritis and GC, and in mice infected with *H. pylori*. DLC1 positivity inversely correlated with tumour progression in patients. GC cells retained an N-terminal truncation variant DLC1v4 in contrast to full-length DLC1v1 in non-neoplastic tissues. *H. pylori* and CagA downregulated *DLC1v1*/*4* promoter activities. DLC1v1/4 inhibited cell migration and counteracted CagA-driven stress phenotypes enforcing focal adhesion. CagA and DLC1 interacted via their N- and C-terminal domains, proposing that DLC1 protects against *H. pylori* by neutralising CagA. *H. pylori*-induced DLC1 loss is an early molecular event, which makes it a potential marker or target for subtype-aware cancer prevention or therapy.

## Introduction

Chronic gastritis following infection with the gram-negative bacterium *Helicobacter (*H*.) pylori* is a risk factor for gastric cancer (GC) [1], especially by strains positive for the virulence factor cytotoxin-A (CagA) [2]. Among the four consensus-molecularsubtypes (CMS) [3], mutations in RHOA and RHO GTPase-activating proteins (GAPs) are major drivers of diffuse GC (CMS2). These mutations foster constitutive GTPase activities [4], conferring adverse prognosis and therapeutic resistance [5]. Thus, identifying proteins/drugs that inhibit RHO signalling remains an unmet medical need.

Tumour suppressor gene “deleted-in-liver-cancer-1” (*DLC1*) is frequently silenced in GC owing to methylation or genomic rearrangements [6]. Human [7] and murine [8] *DLC1/Dlc1* genes are expressed as multiple mRNA splice variants and protein isoforms. Full-length (FL) DLC1 (DLC1v1, >170 kDa) and N-terminally truncated DLC1v4 (>90 to 130 kDa) share identical multi-domain organisations, containing a sterile-alpha motif (SAM), a serine-rich region, a nuclear localisation sequence (NLS), a caveolae/caveolin-binding motif (Cavbm), followed by RHO GTPase-activating protein (GAP) and steroidogenic acute regulatory protein-related lipid-transfer (START) domains. DLC1 acts on inter-cellular contacts and focal adhesions to alter cell migration and invasion by inducing cytoskeleton reorganisation via GTPases (RHO, RAC, CDC42) and interaction with structural/adapter proteins (catenins, cadherins, caveolins, integrins, talin, tensin, focal adhesion kinase), involved in inflammation, carcinogenesis, and metastasis [6]. DLC1 also functions as a positive predictor of survival and responsiveness to chemotherapy in patients with GC [9, 10].

Based on previous observations that the pathogen-endocytosis receptor caveolin-1 (CAV1) recruits DLC1v4 upon *H. pylori* infection [11], we hypothesised that DLC1 antagonises CagA to neutralise its pro-inflammatory, cell-damaging oncogenic effector functions. Herein, we found that (i) CagA physically interacted with DLC1, (ii) DLC1 inhibited intracellular CagA signalling, and (iii) CagA downregulated transcription from *DLC1v1/4* promoters, proposing DLC1-mediated inhibition of CagA/RHO signalling as a potential therapeutic target.

## Results

### Correlation between DLC1 expression and clinical factors in patients with GC

To detect in situ expression of DLC1 protein, we analysed tissue microarrays (TMAs) from patients with GC. Formalin-fixed paraffin-embedded (FFPE) tissue sections from normal/non-tumour stomach (NT: *n* = 30) and tumour (TU: *n* = 116) cases were stained by immunohistochemistry (IHC) with DLC1 antibody (Ab) against the N-terminus, including the SAM domain (S1a). Quantitative analyses of graded scores displayed prominent DLC1 staining in intestinal-type, differentiated tumours both in the cytoplasm and at membranes, compared with diffusetype, undifferentiated tumours with marked stroma positivity. Compared with epithelial cells of the non-neoplastic gastric mucosa, DLC1 protein was downregulated in tumour cells (*p < 0.05 vs. NT, Kruskal–Wallis test with Dunn post-tests). No differences were detected by Lauren histology (NT: *n* = 30; TU: *n* = 34 diffuse; *n* = 56 intestinal). DLC1 downregulation could be associated with the grade of tumour dedifferentiation (S1b). DLC1 was also reduced by UICC (S1c) and pTNM staging, as determined by tumour size (T) (S1d) and nodal status (N) (S1e). Conversely, DLC1 was upregulated in cases exhibiting metastases (S1f) (TU: *n* = 36 M0; *n* = 11 M1; *p < 0.05 vs. NT, Mann–Whitney test). No significant alterations were recorded in the stroma. Supportive data from independent patient cohorts are provided in Supplementary Results (S2, Tables S3–5).

### Expression of DLC1 variants in human GC cell lines

**Fig. 1:**
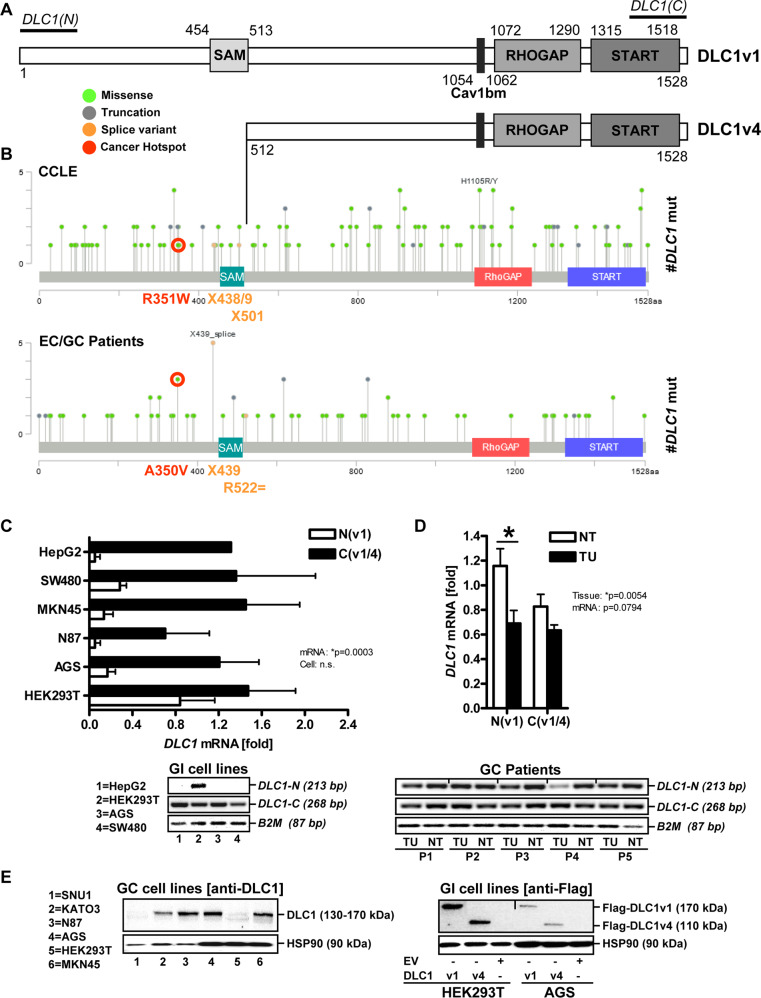
Expression of DLC1 mRNA/protein variants in human GC cells. **A** Scheme of human DLC1 mRNA/protein variants. FL DLC1v1 and the N-terminally truncated DLC1v4 isoform exhibit identical C-terminal multi-domain organisation: sterile-alpha motif (SAM), serine-rich region, nuclear localisation sequence (NLS), caveolin-1 (CAV1)-binding motif (Cavbm), RHO GTPase-activating protein (GAP), and steroidogenic acute regulatory protein-related lipid-transfer (START) domains. DLC1v4 (start codon: MKLEI, aa 513-1528) used in the present study lacks the SAM region compared to DLC1v1 (aa 1-1528). **B** Comparison of putative driver splice mutations in the human FL *DLC1v1* gene located N- and C-terminal of the SAM domain where the *DLC1v4* transcript starts. Point mutation data were retrieved from cBioPortal (Table S6) based on COSMIC/GISTIC entries. Cancer hotspots (red) and splice variants (yellow) are similar in human cancer cell lines (CCLE) and pan-cancer studies (from patients with EC/GC). **C** Detection of *DLC1* mRNA variants. Total RNA from human gastrointestinal cancer and non-cancer (HEK293T) cell lines were subjected to RT-qPCR using primers directed against N- and C-terminal regions of the FL *DLC1v1* cDNA. Quantitative analyses (top) and representative images from PCR-amplification products upon ethidium bromide-stained agarose gel electrophoresis (bottom). Ct-values were normalised to *B2M* and calculated as -fold ± S.E. (*p < 0.05 N vs. C, 2way-ANOVA with Bonferroni post-tests, *n* = 3 per cell line). **D** Downregulation of *DLC1* mRNA variants in human GC. Total RNA from matched TU and NT tissues of patients with GC (*n* = 5 cases) was subjected to RT-qPCR using primers directed against N- and C-terminal regions of FL *DLC1* cDNA. Quantitative analyses (top) and representative gels (bottom). Ct-values were normalised to *B2M* and calculated as -fold ± S.E. (*p < 0.05 TU vs. NT, 2way-ANOVA with Bonferroni post-tests). **E** Detection of endogenous (left, source file: “Original Western Blots” page 2) and ectopic (right, source file: page 3) DLC1 protein variants. Cells (HEK293T, AGS) were transfected with EV or DLC1 expression plasmid for 48 h, followed by extraction of total cell lysates. FL DLC1v1 (170 kDa) was detected by an Ab specific for the N-terminus/SAM + domain of the protein (PA5-18290), ΔSAM DLC1v4 (110 kDa) by anti-FLAG Ab. Representative images from Western blots. Note the presence of endogenous DLC1 (130–170 kDa) in GC cells detected by the Ab directed against the N-terminal part of DLC1, including the SAM domain.

To explore the expression of DLC1 variants in GC [7] (Fig. 1A), the *DLC1* gene was examined for COSMIC/GISTIC entries on somatic mutations and copy number variations (Table S6). Common putative driver splice mutations were detected at identical or adjacent codons (aa 438/439/501/522) within the human FL *DLC1v1* gene and located N- and C-terminal of the SAM domain where the *DLC1v4* CDS (aa 512) begins (Fig. 1B). Cancer hotspot mutations and splice variants were similar in cancer cell lines (CCLE) and pan-cancer patient studies (from EC/GC).

To measure *DLC1* mRNA in human gastrointestinal cancer and non-cancer (HEK293T) cell lines, primers were designed against N- and the C-terminal parts of the FL *DLC1* cDNA. Consistent with previous data [11], RT-qPCR analyses (Fig. 1C) revealed that FL *DLC1v1* was present in HEK293T, whereas truncated *DLC1v4* was detectable in all cancer cell lines examined (*p < 0.05 N vs. C, 2way-ANOVA with Bonferroni post-tests, *n* = 3 per cell line).

RT-qPCR analyses of total RNA extracted from frozen samples of matched normal NT and TU tissues from patients with GC (Fig. 1D) confirmed that *DLC1v1* mRNA was reduced by ~40% compared with controls (*p < 0.05 TU vs. NT, 2way-ANOVA with Bonferroni post-tests, *n* = 5 cases).

Similar results were observed at the protein level (Fig. 1E). We amplified the *DLC1v4* (start codon: MKLEI, aa 513-1528) CDS lacking the SAM region when compared with FL *DLC1v1* (aa 1-1528) [7]. The cDNAs were inserted into FLAG-tagged expression vectors and transfected into HEK293T or AGS cells for 48 h. Western blotting confirmed overexpression of FL DLC1v1 (170 kDa) and N-terminally truncated ΔSAM DLC1v4 (110 kDa). A smaller sized, endogenous DLC1 protein (130–170 kDa) was detected in GC cell lines (AGS, MKN45, KATO3, N87) using an Ab specific for the N-terminal part, including the SAM domain, but not in HEK293T or SNU1, indicative of cell line-specific isoforms.

### *H. pylori* and CagA downregulate DLC1 expression in vivo

**Fig. 2:**
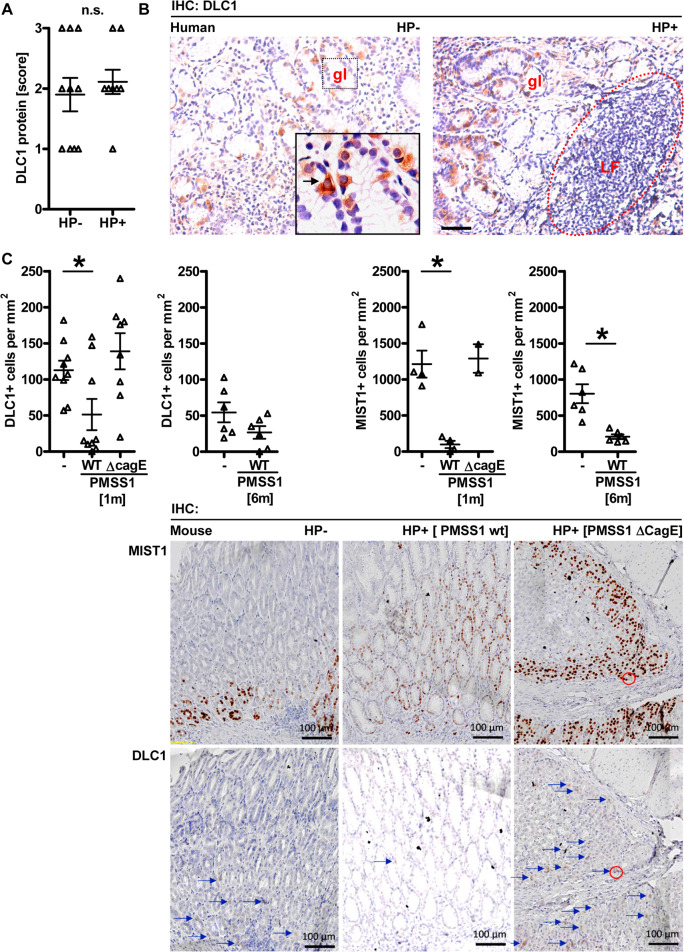
*Helicobacter pylori* infection downregulates DLC1 expression in vivo. **A** DLC1 positivity in uninfected and *H. pylori*-infected human gastric biopsies. Sections from FFPE human stomach tissue (*n* = 9 Hp+ vs. *n* = 10 Hp- cases) from endoscopic material were stained with DLC1 Ab by IHC. Positivity scores for DLC1 were correlated to the infection status. Data are absolute case numbers after dichotomous grouping of stainings as negative (*scores 0/1*) vs. positive (*scores 2/3*) (n.s., Hp+ vs. Hp-, Fisher-Exact test). **B** *H. pylori*-induced inflammatory cell infiltrates outcompete DLC1+ cells in human gastric biopsies. Representative IHC images from (**A**) of the corpus region (left, healthy; right, gastritis). Colour code: Brown = DLC1; Blue = nuclei (hematoxylin). Original magnifications ×200; Scale bar = 50 µm. Note the displacement of DLC1+ cells by accumulating intramucosal leucocytes in inflammatory regions. **C** *H. pylori* downregulates DLC1+ positivity in vivo. C57BL/6J mice were infected with mouse-adapted CagA+ injection-proficient *H. pylori* PMSS1 WT strain or CagA-injection-deficient isogenic mutant Δ*CagE* for 1 or 6 months. Sections from FFPE gastric tissue were stained with Abs against chief/stem cell marker MIST1 or DLC1. Quantitative analyses (top) and representative IHC images of the corpus region (bottom). Data are cell numbers per mm^2^ (*p < 0.05 vs. WT, Kruskal–Wallis test with Dunn or Sidak post-tests, *n* = 2–9 mice per group or time point). Colour code: Brown = DLC1/MIST1; Blue = nuclei (hematoxylin). Original magnifications ×100; Scale bar = 100 µm.

As DLC1 is frequently downregulated in GC, we speculated whether *H. pylori* infection contributes to human or mouse *DLC1/Dlc1* gene silencing. FFPE tissue sections from endoscopic gastric biopsies of naïve and infected individuals were stained by IHC using DLC1 Ab (Fig. 2A). After dichotomous grouping as negative (*scores 0/1*) vs. positive (*scores 2/3*) cases, DLC1 staining was correlated to the infection status (*n* = 9–10 per group). Quantitative analyses of the corpus region exhibited the disappearance and displacement of DLC1 + cells by leucocyte infiltration into inflammatory regions (Fig. 2B). DLC1+ cells accumulated in the periphery of inflammatory follicles. This distribution pattern proposed that *H. pylori* gastritis leads to downregulation of DLC1 positivity in the stomach.

To explore the impact of *H. pylori* virulence factors on DLC1 expression, C57BL/6J mice were infected for 1 and 6 months with the mouse-adapted CagA+ injection-proficient *H. pylori* PMSS1 WT strain or isogenic Δ*CagE* mutant, and DLC1 was visualised by IHC. The CagE mutant cannot translocate CagA into epithelial cells, as CagE is an essential protein for the functionality of the type IV secretion system (T4SS) [2]. Notably, PMSS1 WT reduced the numbers of DLC1+ cells in the stomach of infected mice when compared with uninfected controls (*p < 0.05 vs. WT, Kruskal–Wallis test, *n* = 2–9 mice per group or time point); however, no changes in the numbers of DLC1+ cells were observed in the absence of CagA translocation (Fig. 2C).

**Fig. 3:**
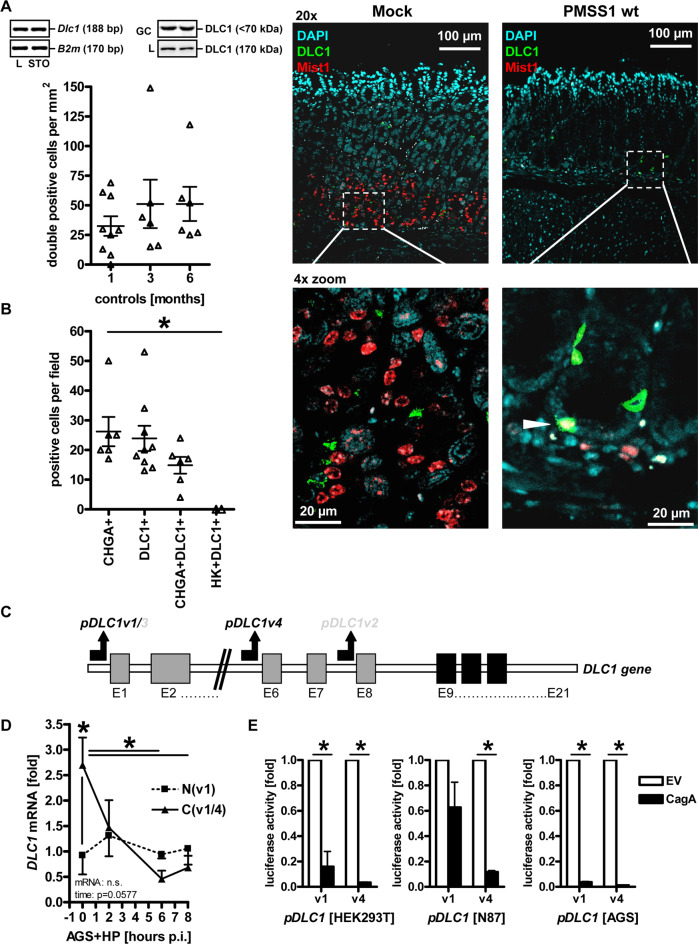
*Helicobacter pylori* and CagA downregulate DLC1 expression in vitro. **A** Partial colocalisation of DLC1 with MIST1+ stem/chief cells. Detection of DLC1 protein in murine gastric chief/stem cells. FFPE sections from stomachs of mock (control) and WT PMSS1-infected (6 months) mice (from Fig. 2C) were co-stained using DLC1 and MIST1 Abs for immunofluorescence microscopy. Quantitative analyses (left) and representative images (right) of the corpus region. Data are numbers of double-positive cells per mm^2^ presented as mean (*n* = 2–9 uninfected control mice per time point, n.c. = no double pos. cases detectable in infected mice). Colour code: Green = DLC1; Red = MIST1; Blue = nuclei (DAPI); original magnifications ×200; Scale bar = 20 and 100 µm. White arrow/frame: Yellow = zoomed-in overlay (DLC1+/MIST1+). Insert (top left): Representative images of PCR-amplification products and murine DLC1 protein isoforms (GC: 70 kDa vs. NT: 170 kDa) from total tissue lysates detected by Western blot using Abs against the N-terminal part of DLC1 including the SAM domain (source file: “Original Western Blots” page 4). Legend: STO = Stomach (Corpus); L = Liver (pos. control); GC = gastric cancer. **B** Colocalisation of DLC1 with ECL cells, but not with parietal cells. FFPE sections from stomachs of uninfected mice were co-stained using DLC1, chromogranin A (CHGA), and H^+^K^+^ATPase (HK) Abs for immunofluorescence microscopy. Quantitative analyses from images of the corpus region presented in **S**3. Data are numbers of single or double-positive cells per field presented as mean (*p < 0.05, Kruskal–Wallis test, *n* = 2–9 uninfected control mice, *n* ≥ 5 fields per image). **C** Scheme of promoters identified in the human *DLC1* gene according to Low et al. [7]. Note that the promoters generate four different transcripts/protein isoforms with identical C-terminus but distinct N-termini. Legend: E = Exon. **D** *H. pylori* downregulates *DLC1* mRNA. AGS cells were infected with CagA+ injection-proficient *H. pylori* strain G27 (MOI = 100) for 3 days. Ct-values from RT-qPCRs on total RNA were normalised to *B2M* and calculated as % ± S.E. (*p < 0.05 vs. mock, 2way-ANOVA with Bonferroni post-tests, *n* = 3). **E** CagA is sufficient for *DLC1* promoter inhibition. Cells (HEK293T, AGS, N87) were co-transfected for 72 h with EV or CagA expression plasmid and reporter vector pGL3 containing the human proximal promoter sequences of *DLC1v1* or *v1*. Luciferase activity was normalised to protein concentration and calculated as -fold ± S.E. (*p < 0.05 vs. EV, one-sample *t* test, *n* = 3 per cell line).

To determine if DLC1 localises to gastric cell types other than ECL cells, the putative stem/chief cell marker MIST1 was examined. Supportive immunofluorescent (Fig. 3A) stainings revealed a scattered distribution of DLC1+ cells in the vicinity of abundant MIST1+ cells at the base of the gland, presenting single double-positive cells, suggesting partial colocalisation. Compared with DLC1+/CHGA+ ECL cells and DLC1+/MIST1+ stem/chief cells, parietal cells showed no colocalisation (Fig. 3B). Notably, after 1 and 6 months of infection, both DLC1+ and MIST1+ cell counts were reduced in PMSS1 WT infected animals vs. littermates receiving the isogenic CagE-deficient mutant (after 1 month) or were uninfected (Fig. 2C), indicating a negative impact of CagE/T4SS-mediated injection of CagA on the expression of both markers.

Conclusively, *H. pylori* CagA downregulated DLC1 in the basal stem cell niche of the gastric gland, which gives rise to both chief and ECL lineages, thus explaining the presence of DLC1 in both cell types. Supportive data on the expression of DLC1 in ECL cells are provided in Supplementary Results (S3).

### *H. pylori* and CagA downregulate DLC1 expression in vitro

An internal promoter is located upstream of exon 9 to allow transcription of N-terminally truncated *DLC1v2/4* mRNAs [7]. Binding motifs for stress-sensitive transcription factors were predicted to bind this promoter (e.g. p53, STAT, HSF), factors known to be also activated by *H. pylori* infection (Fig. 3C).

As shown in Fig. 1, AGS cells expressed *DLC1v4* but no FL *DLC1v1* mRNA, making this cell line suitable to study the effect of CagA on this promoter. AGS cells were infected with the CagA+ injection-proficient *H. pylori* strain G27 (MOI = 100) for 3 days (Fig. 3D). RT-qPCRs revealed a reduction of *DLC1v4* mRNA by ~90% when compared with controls (*p < 0.05 vs. mock, 2way-ANOVA with Bonferroni post-tests, *n* = 3).

To confirm whether *H. pylori* CagA is causal for inhibiting *DLC1* mRNA transcription, we amplified the promoters [7] of *DLC1*v1 and v4, followed by the insertion of genomic DNA sequences into the pGL3 reporter vector. AGS, N87, and HEK293T cells were transfected for 72 h with EV or GFP-CagA-FL expression plasmid, along with *pDLC1v1* or *v4* reporter constructs. CagA reduced luciferase activity of both promoters when compared with control (HEK293T: >95%; AGS: >95%; N87: 10–60%; *p < 0.05 vs. EV, one-sample *t* test, *n* = 3 per cell line) (Fig. 3E).

### DLC1 counteracts cell phenotypes evoked by CagA

Based on the observed CagA-mediated *DLC1* downregulation, we hypothesised that, vice versa, DLC1 inhibits CagA signalling. To explore the mutual antagonism of the two proteins, we examined whether DLC1 prevents CagA-mediated cytoskeleton rearrangements, detectable as spike-like cell elongations, termed the “humming bird” phenotype [2].

**Fig. 4:**
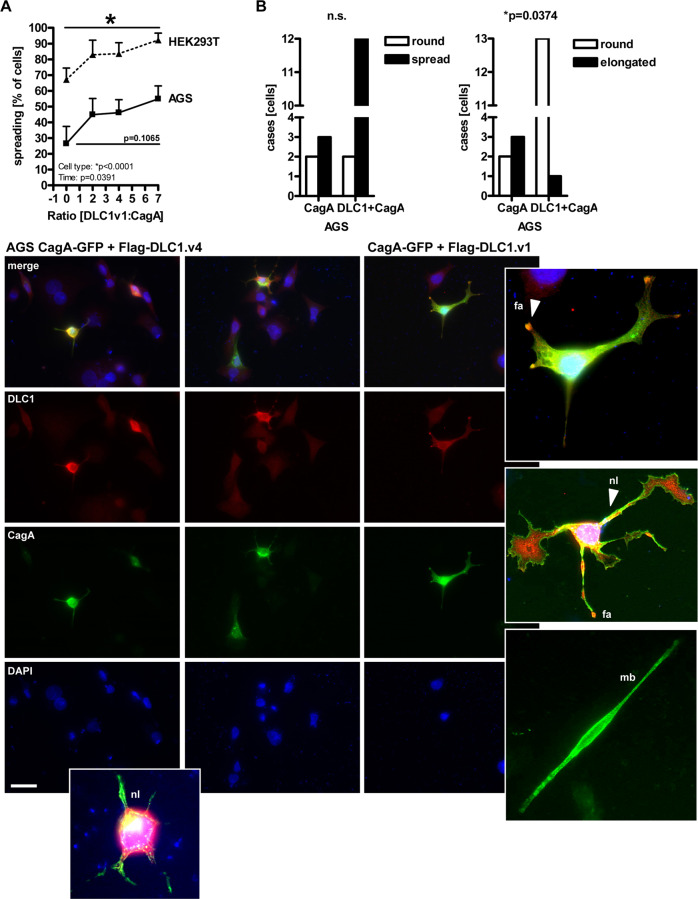
DLC1 counteracts cell elongating (“humming bird”) by CagA. Cells (AGS, HEK293T) were transfected with EV, CagA, or DLC1 expression plasmids or a combination thereof for 36 h, followed by fixation and staining using DLC1 and FLAG Abs for immunofluorescence microscopy. Quantitative analyses (top) and representative images (bottom). Numbers of adherent cells with morphologies (spread-out, round, elongated) were counted, normalised to total cell counts and calculated as % ± S.E. (**A**, HEK293T: *p < 0.05 vs. EV (*t* = 0), 2way-ANOVA Bonferroni post-tests, *n* = 3 per cell line; **B** AGS: *p = 0.0374, Fisher-Exact test; *n* = 3). Colour code: Red = DLC1; Green = CagA (GFP); Yellow = DLC1/CagA (overlay); Blue = nuclei (DAPI); Original magnifications ×400 (zoomed-in 630×); Scale bar = 20 µm. White frames/arrows: DLC1 promotes neurite-like extensions (abbrev. “nl”) (DLC1v4+) or localises to focal adhesions (abbrev. “fa”) (DLC1v1+); membrane-bound (abbrev. “mb”) CagA forms needle-like elongations (“humming bird”).

AGS and HEK293T were transfected with EV, CagA, or DLC1 expression plasmids or a combination thereof for 36 h, followed by fixation and staining using DLC1 or FLAG Ab for immunofluorescent microscopy (Fig. 4A). Numbers of adherent cells normalised to total cell counts were increased (HEK293T: *p < 0.05 vs. EV, 2way-ANOVA with Bonferroni post-tests, *n* = 3 per cell line). DLC1 was localised to the periphery of spread-out cells and in focal adhesions (DLCv1) or neurite-like extensions (DLC1v4), whereas CagA was targeted to the plasma membrane and promoted needle-like cell elongations (“humming bird”). In cells receiving both plasmids, DLC1 reduced CagA-dependent formation of stress spikes (AGS: *p = 0.0374, Fisher-Exact test; *n* = 3) (Fig. 4B). Similar results were obtained for AGS (S4) and HEK293T (S5) cells. Supportive data on DLC1v1/4 effects on morphologies are provided in Supplementary Results (S6–8).

### DLC1 inhibits CagA-mediated oncogenic signalling

Downstream signalling cascades were assessed to elucidate molecular mechanisms on how CagA and DLC1 functionally antagonise each other. AGS cells were transfected with EV or DLC1v1 for 24 h, followed by serum-deprival for 16 h, and restimulation with FCS (20% *v/v*) or EGF (50 ng/ml) for the indicated times. Western blotting (S9a) demonstrated that DLC1v1 augmented phosphorylation of MAPKs (p38, JNK1, ERK1/2: *p < 0.05 vs. EV, 2way-ANOVA, *n* = 3 per cell line) but not of AKT or FAK (not shown).

**Fig. 5:**
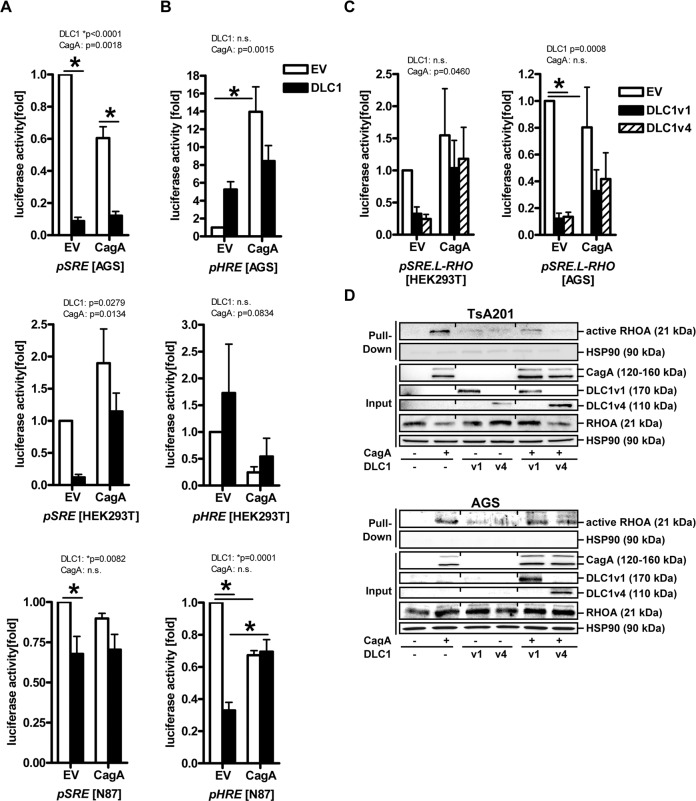
DLC1 inhibits oncogenic signalling by CagA. **A** DLC1 inhibits c-*FOS* promoter activity. Cells (HEK293T, AGS, N87) were co-transfected for 72 h with EV, DLC1v1, or CagA expression plasmids or combinations thereof along with pGL3 reporter vector containing the SRE from the human c-*FOS* gene promoter. Luciferase activity was normalised to protein concentration and calculated as -fold ± S.E. (*p < 0.05 vs. EV, 2way-ANOVA with Bonferroni post-tests, *n* = 3 per cell line). **B** DLC1 inhibits *HMGB2* promoter activity. Cells were transfected as in (**A**), along with pGL3 reporter containing the HRE from the human *HMGB2* gene promoter. Luciferase activity was normalised to protein concentration and calculated as -fold ± S.E. (*p < 0.05 vs. EV, 2way-ANOVA with Bonferroni post-tests, *n* = 3 per cell line). **C** DLC1 inhibits RHO-driven promoter activity. Cells were transfected as described above, with EV, DLC1v1/4, or CagA expression plasmids, along with pSRE.L reporter plasmid containing an RHO-driven modified SRE. Luciferase activity was normalised to protein concentration and calculated as -fold ± S.E. (*p < 0.05 vs. EV, 2way-ANOVA with Bonferroni post-tests, *n* = 3 per cell line). **D** DLC1 inhibits CagA-driven activation of RHOA. TsA201 (top, source file: “Original Western Blots” page 5–9) and AGS (bottom, source file: page 10–14) cells were co-transfected for 72 h with EV, DLC1v1/4, or CagA expression plasmids or combinations thereof. Total cell lysates were subjected to GST-RHOA pulldown assays and Western blotting to detect active and total (input) RHOA, respectively. Representative images are shown.

Next, we examined whether DLC1v1-mediated activation of stress/growth-related kinases is translated to the nucleus towards *c-FOS* transcription, a cell proliferation surrogate (Fig. 5A). HEK293T, AGS and N87 cells were co-transfected for 72 h with EV, DLC1v1, or CagA expression plasmids or combinations thereof, along with pGL3 reporter containing the SRE from the human c-*FOS* gene promoter. Compared with CagA-transfected controls, luciferase activity was reduced by DLC1v1 to 10% (*p < 0.05 vs. EV, 2way-ANOVA with Bonferroni post-tests, *n* = 3 per cell line).

*H. pylori* triggers oxidative stress by releasing reactive oxygen species from infected host cells [12]. To determine whether DLC1v1 interferes with hypoxia-driven stress responses (Fig. 5B), cells were transfected as above with a pGL3 reporter containing the HRE from the promoter of the human *HMGB2* gene [13]. DLC1v1 decreased luciferase activity to 20% when compared with CagA-transfected controls (*p < 0.05 vs. EV, 2way-ANOVA with Bonferroni post-tests, *n* = 3 per cell line). Overall, FL DLC1v1 protected host cells from CagA-mediated damaging and oncogenic effects.

DLC1 inhibits RHOA via its RHO-GAP domain [6], whereas CagA activates RHOA [14]. We thus examined whether DLC1 inhibits CagA-driven RHOA activity. Cells were transfected as before with EV, DLC1v1/4, or CagA plasmids, along with a modified pSRE.L reporter [15] containing an RHO-driven SRE deficient for the TCF-binding site in the human c-*FOS* promoter (Fig. 5C). Both DLC1 isoforms reduced RHO-driven luciferase activity compared with CagA-transfected controls. Moreover, DLC1 counteracted RHO-driven reporter gene expression in presence of CagA overexpression (*p < 0.05 vs. EV, 2way-ANOVA with Bonferroni post-tests, *n* = 3 per cell line).

Specificity was verified by employing an RHOA-activating plasmid expressing a constitutively active α subunit of heterotrimeric G-protein α13 (G13qL) [16] and an RHO-inhibiting plasmid encoding for *C. botulinum* C3 toxin (C3T) [17] targeting RHO. C3T abolished CagA-mediated activation of the RHO-driven SRE, whereas DLC1-mediated inhibition of the SRE pertained in presence of G13qL (S9b).

GST-pulldown assays confirmed these findings using recombinant RHO-binding domain of rhotekin as bait for binding active RHOA (Fig. 5D). TsA201 cells were co-transfected with EV, DLC1v1/4, or CagA plasmid or combinations for 72 h. Total cell lysates were subjected to pulldown assays and Western blotting to detect active and total (input) RHOA. CagA increased G-protein-coupled RHOA activation, an event inhibited by both DLC1 isoforms when compared with input controls. Consistently, RHOA specificity was verified by C3T and G13qL for RHOA pulldown. C3T inhibited CagA- and G13qL-mediated RHOA activation (S9c).

### DLC1 interacts with and neutralises CagA

**Fig. 6:**
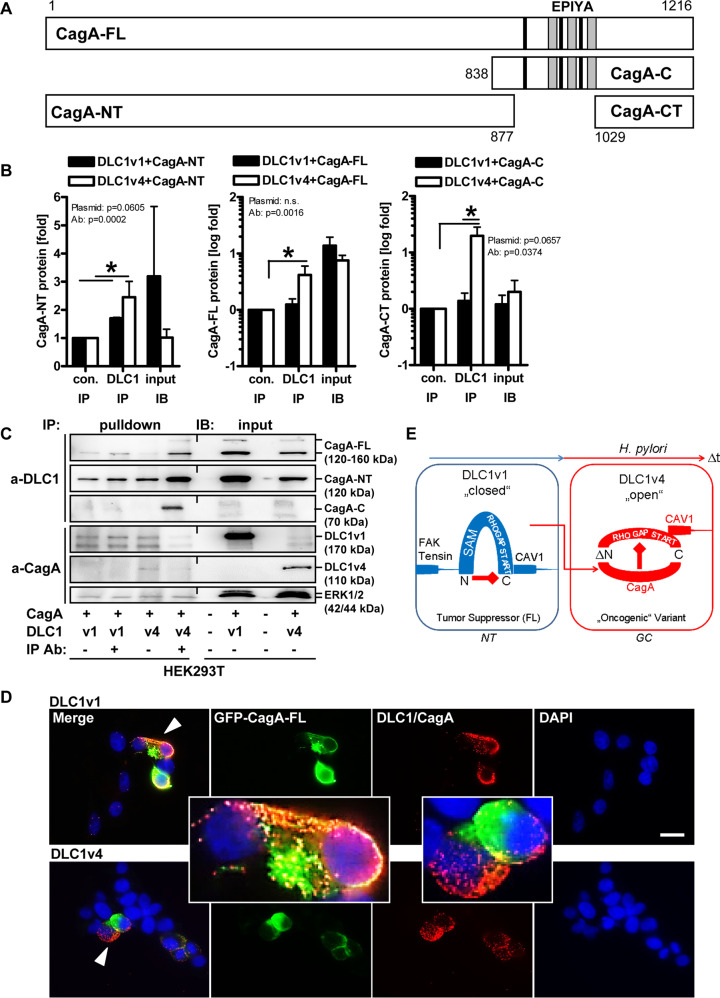
DLC1 interacts with CagA. **A** Scheme of DLC1 and CagA expression constructs. Legend: SAM = sterile-alpha motif, RHO-GAP = RHO GTPase-activating protein (catalytic domain for RHO inhibition); START = StAR-related lipid-transfer (domain), Cav1bm = caveolin-1 binding motif, EPIYA = Src kinase tyrosine phosphorylation motif; grey = multimerisation domain. **B**, **C** CagA interacts with DLC1. HEK293T cells were transfected with CagA-FL (aa 1-1216), CagA-NT (aa 1-877), or CagA-C (aa 838-1216), combined with DLC1v1 or v4 expression plasmids, respectively. CoIP was performed on cell lysates using Abs against CagA, DLC1 (v1) and FLAG (v4). Quantitative analyses (**B**) and representative images (**C**) from Western blots (Source file: “Original Western Blots” page 15–17). OD values from gels were normalised to ERK1/2 (as loading control) and presented as -fold ± S.E. (*p < 0.05 vs. bead control, 2way-ANOVA with Bonferroni post-tests, *n* = 3 per plasmid). **D** HEK293T cells were transfected with CagA-FL (aa 1-1216) in combination with DLC1v1 or v4 plasmids. Representative images from PLA microscopy. Colour code: Red dots = DLC1/CagA complex; Green = CagA (GFP); Blue = nuclei (DAPI); original magnifications ×630; Scale bar = 10 µm. Note accumulation of DLC1+ PLA complexes in CagA-GFP+ cell areas (yellow = overlay). **E** Model of DLC1 and CagA cross-talk in GC. Legend: NT = non-tumour healthy epithelial (“stem”) cell; GC = gastric cancer cell. Normal cells express FL DLC1v1 protein in a “closed” conformation mediated by intra-molecular interaction between the N- and the C-terminus, tethering it to focal adhesion complexes and cellular junctions. Tumour cells express N-terminally truncated DLC1v4 protein lacking this intra-molecular interaction, resulting in an “open” conformation recognised by CagA and promoting stress and malignancy-related cell phenotypes. *H. pylori* infection may evoke loss (LOF) of DLC1v1 and gain (GOF) of DLC1v4 isoforms over time (Δt) during chronic infection.

We inferred that DLC1 neutralises oncogenic and cell-damaging effects of CagA by physically interacting with the two proteins. Several CagA deletion constructs were generated as described previously (Fig. 6A) [18]. CagA-FL (1-1216 aa) plasmid was complemented by two C-terminal fragments, CagA-C (838-1216 aa) and CagA-CT (1029-1216 aa), and the N-terminal fragment, CagA-NT (1-877 aa). CagA-CT and CagA-NT did not contain EPIYA motifs and multimerisation domains. DLC1 plasmids included FL DLC1v1 and truncated DLC1v4 without SAM domain [7]. DLC1 constructs were FLAG-tagged, CagA mutants fused to GFP.

CoIP experiments were performed to verify complex formation between the two proteins. HEK293T cells were transfected with CagA-FL combined with DLC1v1 or v4 plasmids. IP was performed on cell lysates using Abs against CagA or DLC1 (Fig. 6B, C). Western blotting corroborated the interaction between CagA-FL and both DLC1 isoforms. We observed that DLC1v4 was more efficiently precipitated than DLC1v1 (*p < 0.05 vs. bead control, 2way-ANOVA with Bonferroni post-tests, *n* = 3 per plasmid). To determine which CagA domains are necessary for the interaction, cells were transfected with CagA-NT or CagA-C in combination with DLC1v1 or v4 plasmids, respectively. Both N- and C-terminal parts of FL CagA interacted with the two DLC1 isoforms. Again, DLC1v4 was more efficiently precipitated than DLC1v1 (*p < 0.05 vs. bead control, 2way-ANOVA with Bonferroni post-tests, *n* = 3 per plasmid). PLA microscopy confirmed the proximity of the two proteins. Oligonucleotide-labelled secondary Abs generated a red fluorescent signal on colocalisation of GFP-CagA and DLC1 (Fig. 6D). All examined CagA constructs interacted with both DLC1 isoforms (S10).

Collectively, these findings suggested that DLC1 binds and neutralises CagA in vitro [11]. Supportive data on phenotypes of *Dlc1*-deficient mice are shown in Supplementary Results (S11-15), confirming that DLC1 also prevents gastric inflammation in vivo.

## Discussion

Herein, we demonstrated that the RHO GTPase inhibitor DLC1 prevented gastric inflammation in vivo and counteracted the oncogenic actions of CagA, a major virulence factor of *H. pylori* and a risk factor for GC [14]. Mechanistically, we provided evidence indicating mutual functional antagonism of the two proteins, wherein DLC1 inhibits CagA-dependent RHO signalling and, vice versa, CagA prevents transcription from the *DLC1* promoter, thereby lowering DLC1 protein in living cells.

This cross-talk (Fig. 6E) was driven by protein-protein interaction mediated via the N- and C-terminal CagA domains and the internal/C-terminal regions of FL DLC1v1 and ΔSAM DLC1v4. The N-terminal membrane domain of CagA inhibits the signalling activity of the C-terminal EPIYA motifs [18], and the N-terminal SAM domain in DLC1 inhibits the enzymatic activity of the RHO-GAP domain [19]. Hence, steric competition between these mutual intra- and inter-molecular regulatory protein interactions determines the outcome of signalling activities for both proteins. Notably, N-terminally truncated DLC1v4 protein, retaining the C-terminal RHO-GAP and START domains such as the caveolin-binding motif (Cavbm), was recruited to caveolin-rich areas of the plasma membrane by *H. pylori* [11]. Moreover, this truncation variant was present after transformation, whereas FL DLC1v1 was lost in GC cells. Thus, DLCv4, lacking the auto-inhibitory N-terminal domain and/or harbouring other unidentified mutations [20], may gain aberrant oncogenic function in gastric cells, a feature which could explain the observed enrichment of *DLC1* mRNA/protein in diffuse-type (CMS2) and advanced stages of GC with distant metastases [6]. Consistently, therapeutic response in patients with GC has been correlated to DLC1 expression [9, 10].

Genomic alterations in *DLC1* contribute to drug sensitivity and confer growth advantage upon activation of RHO-ROCK signalling, rendering tumour cells more susceptible to ROCK-inhibitors (e.g., Y-27632). Low DLC1 expression also predicts poor therapeutic efficiency of fluoropyrimidine/oxaliplatin in adjuvant chemotherapy and was associated with advanced, metastatic disease and shortened time to recurrence and overall survival. Likewise, ROCK-inhibitor fasudil [21] attenuated murine GC growth in vivo. Joshi et al. have designed cell-permeable peptides derived from the SAM domain of DLC1 [22], which destroy the intra-molecular auto-inhibition between the SAM and the RHO-GAP domains. Thereby, the peptides trigger a conformational reactivation of DLC1, from a “closed” towards an “open” state (Fig. 6E), allowing recruitment and interaction with potent tumour suppressors like PTEN, culminating in decreased RHOA activation, tumour cell growth and migration in vitro, proposing DLC1 as a druggable target.

To date, DLC1 has not been functionally connected with *H. pylori* in patients or animal models, although gene hypermethylation was detected upon infection [23]. Similar to humans [7], at least four murine splice/protein variants have been identified [8]. Gene trapped *Dlc1*^*gt/+*^ mice harbour an insertion between exon 1 and 2 of the 6.1 kb transcript and exhibit developmental defects and disease-prone phenotypes [8, 24, 25]. However, *Dlc1*^*gt/+*^ mice do not per se progress to cancer, suggesting that cooperative damage cues or oncogenic mutations are required for transformation. Consistently, we showed that *Dlc1*-deficient animals exhibit gastritis and disturbed expression of neuro-entero-endocrine hormones, characterised by moderate leucocyte infiltration and enhanced proliferation of epithelial cells, indicating an early step in the transition from inflammation to malignancy (S15). The highest in situ expression of DLC1 protein both in mouse and human gastric tissue was found in ECL cells [26], known to produce a majority of the gut peptide and histidine/tryptophan-derived hormones [27]. Therefore, DLC1 may play an additional role in stomach homeostasis via auto/paracrine regulation of secretory factors, in addition to its well-established function in epithelial cells as a cytoskeleton regulator. *H. pylori* infection correlates with deterioration of the integrity and secretory activity of ECL cells (e.g. somatostatin producing D cells [28]) and hypergastrinemia (by gastrin producing G cells [26]), resulting in altered histamine release and subsequent gastric acid secretion from adjacent parietal cells in the oxyntic mucosa and pre-neoplastic alterations in humans and animal models. Consistent with this sequence of events, *Dlc1*^*gt/+*^ mice exhibited reduced levels of several ECL hormones, primarily somatostatin [29], which counteracts the pro-angiogenic and trophic effects of gastrin [27]. However, further in-depth studies need to clarify the causality, considering the unidentified stem cell origin of gastric adenocarcinoma and neuroendocrine tumours (carcinoids).

According to Lauren’s classification, *H. pylori* can be associated with intestinal GC, via the “Correa” sequence of histomorphological changes, and with diffuse GC [2], in addition to sporadic mutational events causative for this subtype. The current CMS classification [3] highlights the function of RHO signalling in diffuse (CMS2) GC. This is underscored by the fact that several mutations in *RHOA* and RHO GAP (e.g. CLDN18-ARHGAP6 or CTNND1-ARHGAP26 fusions) genes, to which DLC1 (also termed ARHGAP7) also belongs, were identified as oncogenic drivers for diffuse GC [3, 4]. Therefore, we propose that loss of the RHO-inhibitor DLC1 correlates with *H. pylori* infection in certain GC subtypes. Despite the declining incidence of intestinal GC, that of diffuse GC continues growing and is characterised by poor prognosis and rapid metastasis. Thus, there is an urgent medical need to specifically address this GC subtype. Consistently, our data propose that novel subtype-specific treatment strategies could be developed for RHOA inhibition.

## Materials and methods

### Subjects

All patients provided written informed consent. Studies were performed in accordance with the principles of the Declaration of Helsinki and approved by the Medical Ethics Committees of the Universities of Heidelberg, TU München and Kiel. Patients’ tissue specimens were collected after surgical resection, stored and classified by Lauren’s histological classification. Tissue microarrays (TMAs) were custom-made [30] or purchased from US Biomax (Rockville, MD).

### Animals

Tissue biobanks from C57BL/6J mice infected with *H. pylori* strains SS1 [11] or PMSS1 [31] were generated previously. *Dlc1*^*gt/+*^ mice [8] were bred in a pathogen-free mouse facility. Animal studies were conducted in agreement with animal welfare guidelines and approved by the governments of Bavaria and Baden-Württemberg (55.2-1-54-2531-74-08/-99-10; -2532-155-12; 35-9185.81/G-146/15; 55.2-1-55-2532-196-2016).

### DNA-constructs

The ~1 kb fragment of the proximal human *DLC1v1* (NC_018919.2; position 135.59.304–135.60.451) promoter [7] was amplified by PCR from genomic DNA of normal human liver and inserted into the KpnI/SacI sites of pGL3-luc luciferase reporter plasmid (Promega, Mannheim, Germany). The upstream ~0.6 kb region of the proximal human *DLC1v4* (NG_015998.1; position 242.923-243.520) promoter [7] was amplified as detailed above and inserted into the KpnI/HindIII sites of pGL3-luc. Isoform 1 of FL human *DLC1* cDNA (NM_182643, start aa 1 MSVAI, *DLC1v1*) was obtained in pCMV-SPORT6.1 (Open Biosystems, Thermo Fisher Scientific, Waltham, MA) and subcloned into the BamHI/NotI sites of mammalian expression vector pTarget (Promega). Isoform 4 of human *DLC1* cDNA (NM_001164271.1; ΔSAM, start aa 513 MKLEI, *DLC1v4*) [7] was amplified from total RNA isolated from human hepatoma HepG2 cells and inserted into pTarget, as described above. Both *DLC1* cDNA variants were FLAG-tagged on their respective N-termini. The pEGFP fusion constructs with CagA-FL, CagA-N (aa 1-877) and CagA-C (aa 838-1216), and pTRE-Tight with GFP-tagged CagA-CT (aa 1029-1216) were described previously (both from BD Biosciences, San Jose, CA) [18]. Firefly luciferase reporter gene plasmids were pHRE-luc [13] driven by the human *HMGB2* promotor in pGL3 (Promega), pSRE-luc (Stratagene/Agilent, La Jolla, CA) and pSRE.L-luc containing a mutated TCF-binding-deficient SRE from the human *c-FOS* promotor [15], and renilla luciferase control plasmid pR.L-TK-luc (Promega). Expression plasmids for RHO pulldown assays were GST-tagged RHO-binding domain (aa 7-89) of rhotekin in pGEX-2T (Merck/Sigma), C3 toxin of *Clostridium botulinum* (C3T) [17], and G-protein α subunit 13 (G13qL) [16] in pcDNA3 (Thermofisher).

### Reagents

Chemicals were from Merck/Sigma (Darmstadt, Germany). Primary antibodies (Abs) are listed in Table S1. Secondary Abs were anti-mouse or rabbit IgG conjugated to Alexa Fluor® 488/594 (Thermofisher) or ECL™ HRP (GE Healthcare, Stafford, UK).

### Cell culture

Human TsA201 cells were purchased from the European Collection of Authenticated Cell Cultures (ECACC, Salisbury, UK); MKN45 from the German Collection of Microorganisms and Cell Cultures (DSMZ, Braunschweig, Germany); HEK293T and other human cancer cell lines (Gastric: AGS, N87, MKN45, SNU1, KATO3; Colorectal: SW480; Liver: HepG2) from the American Type Culture Collection (ATCC, Rockville, MD). All lines were maintained as suggested by the distributors. Transient transfection, reporter gene and cellular assays are described in Supplementary Methods.

### Bacterial culture and infection of mammalian cells

*H. pylori* wildtype (WT) SS1 (Sydney strain, CagA+ injection-deficient, mouse-adapted), PMSS1 (pre-mouse Sydney strain, CagA+ injection-proficient, mouse-adapted), and its CagA-injection-deficient isogenic mutant (*ΔCagE*) or G27 (clinical isolate, CagA+ injection-proficient, cell culture-adapted) were recovered from −80 °C glycerol stocks and grown on blood agar plates under microaerobic conditions as detailed in Supplementary Methods [11, 31]. Bacteria were harvested from agar plates, propagated on AGS cell monolayers for in vitro experiments or animal inoculations.

### Reverse transcription PCR (RT-PCR) and quantitative PCR (qPCR)

Total RNA was extracted from cells or frozen tissues with RNeasy™ Mini Kit (Qiagen, Hilden, Germany) followed by cDNA synthesis (Verso™ cDNA Kit, Thermofisher). Two-step PCR (95 °C 15 s, 60 °C 1 min, 40 x cycles) was performed using Power SYBR^®^ Green Master Mix and ABI PRISM Real Time 7900HT device (Thermofisher). Relative mRNA expression was calculated with the ∆∆Ct method [32]. Oligonucleotides are listed in (Table S2) [33].

### RHO pulldown, co-immunoprecipitation (CoIP) and Western blotting

GTPase activity (pull-down) assays were performed as detailed by the manufacturer (Biozol/Cell Biolabs Inc., San Diego, CA) or custom-made [16]. Procedures are presented in Supplementary Methods [11].

### Immunofluorescence

Proximity ligation assay (PLA) was conducted as recommended by the manufacturer (Duolink® In Situ Red Starter Kit Mouse/Rabbit, Merck/Sigma). Staining protocols for cell lines and formalin-fixed and paraffin-embedded (FFPE) tissue sections are given in Supplementary Methods. Slides were visualised in triple-colour mode using a digital camera-connected (Axiovision, release 4.4) fluorescence microscope (Axiovert 200M, Carl Zeiss MicroImaging, Hallbergmoos, Germany). Single or double-positive cells (*n* > 5 per field; *n* = 5 fields per image) were quantified with ImageJ.

### Immunohistochemistry (IHC)

Procedures for Ab (Table S1) staining on FFPE tissue sections were performed as described by the Vectastain ABC kit (Vector Labs, Burlingame, CA) using 3,3-diaminobenzidine (DAB), followed by hematoxylin counterstaining. Frequency and intensity of positivity [30] were determined in epithelial and lamina propria (stroma) cells [Scores: 0+= negative (0–25%), 1+= weak (25–50%), 2+= moderate (50–75%), and 3+= strong (75–100%)]. Percentages were calculated as positive cells divided by the total number of cells per field. Signals were quantified by a blinded observer using a bright-field microscope (*n* > 10 cells per field; *n* = 5 fields per image) or BX61VS slide scanner (Olympus, Hamburg, Germany). Field areas were defined by the morphology of a given anatomical structure, such as the foveolar-neck-base unit, and measured in mm^2^.

### Statistics

Sample size estimates (POWER 0.8) for animal experiments were calculated with SAS (Cary, NC). Results are displayed as means ± standard error (S.E.) from at least three independent experiments (*n* ≥ 3) from different cell passages or individuals (mice, patients). Optical densities (ODs) of bands in gels from Western blots and PCRs were measured using automated imaging devices and quantified with ImageJ (Washington, DC). Data were normalised to house-keeping genes or proteins and calculated as -fold change or % when compared with control. Statistical analyses were performed with GraphPad Prism (La Jolla, CA). Data were tested for non- vs. parametric distribution and equal vs. unequal variance, followed by the recommended test with correction for multiple comparisons. Tests were 2-sided and unpaired if not stated otherwise in the legends to figures. *P*-values < 0.05 were considered significant (*).

## Supplementary information

Supplementary Methods and Results

Supplementary Tables

Supplementary Figure Legends

Supplementary Figure S1

Supplementary Figure S2

Supplementary Figure S3

Supplementary Figure S4

Supplementary Figure S5

Supplementary Figure S6

Supplementary Figure S7

Supplementary Figure S8

Supplementary Figure S9

Supplementary Figure S10

Supplementary Figure S11

Supplementary Figure S12

Supplementary Figure S13

Supplementary Figure S14

Supplementary Figure S15

Original Western Blots

## Data Availability

For all data requests, please contact the corresponding author.
