# Supplementary material for: Functional antagonism between CagA and DLC1 in gastric cancer
Source: Cell Death Discov. 2022 Aug 13;8:358. doi: 10.1038/s41420-022-01134-x (PMC9376073; doi:10.1038/s41420-022-01134-x)
Supplement: Supplementary file 1 — Supplementary Methods and Results [file 41420_2022_1134_MOESM1_ESM.doc]

**APPENDIX**

**Supplementary Methods**

**Cell culture**

AGS, SW480, HepG2, HEK293T and tsA201 were cultivated in DMEM, while N87, MKN45 and SNU1 in RPMI 1640 medium. All lines were grown at 37 °C under 5 % (*v/v*) CO_2_ in a humidified atmosphere. Media, herewith defined as “complete”, were supplemented with 1 % penicillin/streptomycin, 1 % L-glutamine and 10 % FCS. At 80-90 % confluency, cells were detached and reseeded using 0.25 % Trypsin/EDTA solution (all *v/v*, Thermofisher). For serum deprival experiments, RPMI 1640 medium or DMEM was without any supplements, designated as “basal medium”. Cell lines were tested for mycoplasma (Invivogen, Toulouse, France).

**Transient transfection**

Cells were seeded in 6-well plates (0.5-0.75 x 10^6^/well) and grown overnight to adhere. Then, the cells were transfected with plasmid DNA in basal media using TurboFect™ reagent (Thermofisher) according to the manufacturer’s instructions. After 16 h (HEK293T: 6 h), the transfection mixture was removed and replaced with complete medium, and the transfectants were incubated for additional 24 h for subsequent analyses.

**Luciferase activity assay**

Cells were lysed in 1 x lysis buffer (100 μl per well of a 6-well plate, Promega) under constant shaking for 10 min at RT. After centrifugation, reporter gene activity was measured in supernatants by Steady-Glo® luciferase assay system (Promega) using Tecan Infinite M200 microplate reader (TECAN Group, Männedorf, Switzerland). Firefly luciferase counts were normalized to the protein content of each sample or to renilla luciferase (pRL-TK) employing the Dual-Luciferase® Reporter Assay System (Promega).

***H. pylori* culture**

*H. pylori* G27 strain was plated on Columbia blood agar plates with 10 % (v/v) horse blood and Dent supplement (Oxoid, Wesel, Germany). The plates were incubated in sealed air-proof plastic boxes with GasPak™ (BD, Heidelberg) at 37 °C to produce a hypoxic environment. *H. pylori* strain PMSS1 was grown on Wilkins-Chalgren blood agar plates with 10 % (*v/v*) horse blood and Dent supplement (Oxoid) under microaerobic conditions (10 % CO_2_, 5 % O_2_, 85 % N_2_; 37 °C).

**Infection of eukaryotic cells with *H. pylori***

AGS “feeder” cells were grown in complete DMEM medium to 80 % confluency in 75 cm^2^ cell culture flasks. Thereafter, medium was replaced with *H. pylori* cultivation medium [DMEM/F12, 10 % (*v/v*) FCS (PAN-Biotech, Aidenbach, Germany), 10 % (*w/v*) Brucella Broth medium, 1 µg/ml vancomycin]. Bacteria were collected from agar plates using sterile cotton buds and resuspended in 2 ml *H. pylori* cultivation medium. AGS monolayers were overlaid with the bacteria suspension, and the cells were incubated at 37 °C under 5 % CO_2_ in a humidified incubator. After 24 h, cell culture supernatants were collected and centrifuged for 5 min at 4000 rpm. The pellet was resuspended in 2 ml *H. pylori* cultivation medium and used for re-infection of feeder cells or consecutive experiments.

**Infection of mice with *H. pylori***

C57BL6/J mice were bred under specific pathogen-free conditions and housed at the Institute for Medical Microbiology, Immunology and Hygiene (TUM, Munich, Germany). Prior to infection, mice were fasted for 4 h. Mice (6-10 weeks of age, female) were randomized and then infected with *H. pylori* strains (2-3 x 10^8^ CFU) three times by oral gavage in Brucella broth containing 10 % (*v/v*) FCS every other day. Mice were sacrificed after 1-6 months, and gastric tissue was collected for histologic examination.

**Protein extraction**

Adherent cells were washed with PBS before adding lysis buffer (50 mM Tris-HCl, pH 7.4, 1 % (*w/v*) SDS, 1 mM Na_3_VO_4_, 1 mM DTT, Protease Inhibitor Complete®, Roche) for 10 min on ice. Cells on plates were scraped off and lysates sonicated for 20 s. Frozen tissue (2-3 mm³) was added to ice-cold lysis buffer (HEPES 20 mM, pH 7.4, EDTA 1 mM, β-glycerophosphate 50 mM, glycerol 10 % (*v/v*), Triton X-100 1% (*v/v*), 1 mM Na_3_VO_4_, 1 mM DTT, Protease Inhibitor Complete®). Tissue fragments were then homogenized and incubated on ice for 60 min. Finally, cell and tissue lysates were centrifuged at maximum speed for 10 min at 4 °C, and supernatants were subjected to Pierce™ BCA protein assay (Thermofisher) and stored at -80 °C.

**Western Blot**

Gels were loaded with equal amounts of protein per lane (25 µg) for SDS-PAGE, and transfer was visualized by Ponceau Red staining of nitrocellulose membranes (Mini-PROTEAN® Tetra Cell system, Biorad, Hercules, CA). Primary and secondary peroxidase-coupled Abs were diluted as recommended by the manufacturers (**Table S1**). Bands were detected using GE Healthcare Amersham™ ECL Prime Western-Blot Detection Reagent (Thermofisher) and quantified with automated chemoluminescence imaging device (Fusion Solo, Peqlab VWR, Radnor, PA).

**Co-Immunoprecipitation (CoIP)**

Cells were grown to 90 % confluency in 6-cm dishes and incubated on ice for 20 min in 1 ml hypotonic lysis buffer (10 mM Tris-HCl, pH 7.4, 2 mM EDTA, 2 mM MgCl_2_, 1 mM Na_3_VO_4_, 1 mM DTT, Protease Inhibitor Complete®). Cells were scraped off, homogenized by repetitive pipetting and centrifuged for 10 min at full speed at 4 °C. For pre-clearing, the supernatant was mixed with 10 µl Pierce™ Protein A/G Plus Agarose (Thermofisher) and incubated for 1 h at 4 °C under rotation. After centrifugation (as above), an aliquot of the supernatant was stored at -20 °C, termed “input control”. For CoIP, 400 µl supernatant were mixed with 4 µg primary Ab (**Table S1**) or left without (negative “bead only” control) and incubated on a tube rotator overnight at 4 °C. Thereafter, 60 µl Protein A/G PLUS-Agarose were added, and the reaction rotated for additional 2 h at 4 °C. Protein/Agarose complexes were pelleted by centrifugation followed by three washing steps (lysis buffer supplemented with 150 mM NaCl). For elution of the co-precipitated proteins, the pellet was mixed with 50 µl of 100 mM glycine (pH 2.2) and incubated on ice for 2 min. The reaction was stopped by adding 10 µl of 1.5 mM Tris-HCl (pH 8.8). The eluate was collected after centrifugation and, together with the input controls, supplemented with 5x SDS-loading buffer for Western Blot.

**GST Pulldown Assay**

GTPase activity was detected using RHOA/RAC1/CDC42 Activation Assay Combo Kit (Biozol/Cell Biolabs Inc., San Diego, CA) following the protocol provided by the manufacturer. Cells, grown to 90 % confluency on a 6-cm dish, were lysed in 1 ml 1x Assay/Lysis Buffer (containing 1 mM Na_3_VO_4_, 1 mM DTT, Protease Inhibitor Complete®), scraped off and incubate on ice for 15 min. After centrifugation, an aliquot of the supernatant was stored on ice as input control. Samples were mixed with 40 µl agarose beads linked to the RHO-binding domain (RBD) of rhotekin, which is only bound by active GTP-loaded RHOA, and incubated for 1 h at 4 °C on a tube rotator. Beads were pelleted by centrifugation and washed three time with 1x Assay/Lysis Buffer and resuspended in 30 µl 1 M Tris-HCl buffer (pH 7.4) and 10 µl 5x SDS-loading buffer for Western Blot analysis. Custom-made RHOA pull-down assay was performed in tsA201 (HEK293T equivalent) cells. GST-rhotekin-RBD fusion protein in pGEX-2T vector (Addgene, Watertown, MA) was expressed in *E. coli* Rosetta™ bacteria and linked to glutathione sepharose beads. Cells, grown to 90 % confluency in 6-well plates, were lysed in 500 µl GST-fishing buffer per well [50 mM Tris-HCl pH 7.4, 150 mM NaCl, 4 mM MgCl_2_, glycerol 10 % (*v/v*), Igepal® CA-630 1 % (*v/v*)]. After centrifugation, the supernatant (an aliquot was stored as input control) was incubated with 90 µl rhotekin-loaded sepharose beads on ice for 1 h under rotation. Beads were pelleted by centrifugation, washed twice with GST-fishing buffer and finally resuspended in 15-20 µl for addition of 5x SDS-loading buffer and subsequent Western blot analysis.

**Cell immunofluorescence**

Cells were grown to 50 % confluency on sterile cover slips in a 6-well plate, followed by fixation with 4 % (*v/v*) formaldehyde in PBS for 20 min. Cells were permeabilized by detergent buffer (0.1 % Triton® X-100 (*v/v*) in PBS) for 10 min, and unspecific Ab binding sites were blocked with pure FCS for 30 min, all at RT. Primary Ab in dilution buffer (1% FCS (*v/v*) in PBS) was incubated overnight at 4 °C in a humidified chamber. Then, fluorescence-labelled secondary Abs or phalloidin dye (Thermofisher) were added in Ab dilution buffer for 1 h at RT in a humid chamber in the dark. DAPI (50 ng/ml) was added for 10 min to stain the nuclei. After washing, cover slips were transferred onto glass slides using fluorescence mounting medium (Dako/Agilent, Santa Clara, US).

**Tissue immunofluorescence**

Tissue samples were fixed in 4 % (*v/v*) paraformaldehyde in PBS at 4 °C for 24-48 h, followed by dehydration in an automated device (TP 1020, Leica) and manual embedding into liquid paraffin. FFPE blocks were cut into 2-5µm sections using a microtome (Leica RM 2145). Paraffin sections on glass slides were deparaffinized in xylol and rehydrated in 96-70 % (*v/v*) EtOH/H_2_0. Antigen retrieval was performed using Vectastain antigen unmasking solution (pH 6.0, Vector Labs.) and steam-heating for 10 min. Staining procedure was then followed as above for cells with more stringent Ab diluent buffer (10 % FCS, 0.3 % Triton® X-100 in PBS, all *v/v*). Finally, tissue sections were covered with Eukitt® mounting medium (VWR/Merck) and a cover slip.

**Proliferation**

Cell viability was measured using 1-(4,5-dimethylthiazol-2-yl) 3,5-diphenyl-formazan (MTT) (Roche Diagnostics, Mannheim, Germany). Cells were seeded at a density of 2000 cells/well of 96-well plates and grown for 1-7 days before adding MTT reagent (5 mg/ml in PBS, 10 µl per well) for 4 h. Upon overnight incubation with 100 µl MTT lysis buffer (10 % (*w/v*) SDS, 0.01 N HCl) per well at 37 °C. The O.D. was determined at 570-650 nm using a microplate reader (Infinite 200, Tecan, Männedorf, Switzerland).

**Adhesion**

Cells were seeded at a density of 1×10^4^ per 6-cm cell culture dish for 1 to 8 h, followed by repetitive washing with PBS at RT. The remaining adherent cells were fixed with 4 % (*w/v* in PBS) formaldehyde, subsequently stained with 1 % (*w/v* in PBS) crystal violet dye and counted under a standard bright field microscope.

**Migration and invasion**

Cell migration was examined with uncoated control, invasion with MatriGel™-coated (wide pore, 8 µm) trans-well inserts according to the manufacturer’s instructions (BD Biosciences). In brief, chambers were rehydrated with 750 µl complete medium. Cells were seeded onto the circular inserts at a density of 5x10^4^/ml for overnight at 37 °C. Remaining cells in the upper chamber were removed with a cotton swab. Cells migrated to the lower side of the insert were fixed and stained with crystal violet as above. The circular insert was removed from the plate and transferred onto a glass slide with Eukitt® mounting medium (VWR/Merck) and sealed with a cover slip. Cells were counted as above.

**Supplementary Results**

**Correlation of *DLC1* gene alterations to clinical factors in GC patients**

To explore *DLC1* mRNA expression in human GC, we re-analyzed cancer data sets from Oncomine® [1] [Forster Gastric (n=43), **Table S3**]. Overall, *DLC1* mRNA was increased in mixed tumour/stroma samples of diffuse GC compared to normal gastric tissue or intestinal GC (**S2a)**. To relate histomorphological (“Lauren”) to CMS classifications [2], we interrogated *DLC1* mRNA expression based on TCGA entries from the cBioPortal® database [3] [Gastric Adenocarcinoma, TCGA, PanCancer Atlas (n=440) and Nature (n=295)]. *DLC1* mRNA was high in the CMS2 subgroup of GS/diffuse GC compared to the other 3 CMS, indicative of differential expression and function of DLC1 in GC subtypes **(S2b,c).** *DLC1* mRNA up-regulation was further observed in patients with metastatic disease (M1) compared with patients without metastases (M0) **(S2d).** Oncoprint® files confirmed alterations in genes encoding for components of the *RHOA* oncogenic driver pathway for diffuse GC (e.g. *RHOA*, *SRC, CAV1, ROCK1*/2 e.a., **Table S4)**, mainly amplifications and mRNA up-regulations as contrasted by deletions and missense mutations in the *DLC1* gene (**S2e)**. Kaplan-Meier survival analysis associated these changes with a trend (p=0.0518, log-rank test, n=32 studies) towards poor prognosis in patients (**S2f, Table S5)**. Conclusively, *DLC1* mRNA was positively associated with advanced, metastatic and diffuse GC.

**DLC1 is present in gastric ECL, but not in parietal cells**

To identify the cell types which express DLC1 in the non-neoplastic stomach, FFPE tissue sections from gastric biopsies of healthy individuals were stained for IF microscopy using the same Ab as for IHC **(S3a**). In the non-neoplastic human stomach, DLC1 protein was found at the base (crypt) of gastric corpus glands in single cells adjacent to acid-secreting parietal cells. Overlay of co-stainings (yellow colour) with chromogranin A (CHGA), a marker of enterochromaffin-like (ECL) cells connected to neuroendocrine tumours (NETs) and diffuse GC [4], suggested that DLC1 was predominantly expressed in this gastric hormone producing subpopulation. Moreover, the staining was excluded from the nucleus and prominent in the cytoplasm and membrane and did not colocalize with H^+^K^+^ATPase, a marker for parietal cells which was confined to the apical (luminal) part of the gastric foveolae **(S3b**).

Overall, these data indicated that DLC1 is present in human ECL cells previously associated with diffuse GC. Consistently, DLC1 protein was detectable in murine gastric ECL cells **(S3c**). FFPE sections from stomach tissue of C57BL6/J WT mice were stained using DLC1 and chromogranin A or H^+^K^+^ATPase Abs for IF microscopy. Imaging analyses of the murine corpus region confirmed the distribution pattern observed for humans.

**DLC1 inhibits migration and promotes adhesion of GC cells**

Intrigued by the biphasic DLC1 expression in patients, i.e. early loss *vs*. gain in diffuse and advanced GC, we were interested in its function in GC cells. DLC1 actively reshapes the actin cytoskeleton [5], hence, we first measured time-dependent adhesion to surfaces (**S6a**). Subconfluent cells (AGS, HEK293T) were transfected with EV or DLC1v1/4 expression plasmids for 36 h, respectively, followed by re-seeding onto plastic culture dishes for the times indicated before fixation and staining with crystal violet. Numbers of adherent cells were counted, normalized to total cell counts and calculated as means ± S.E. (*p<0.05 *vs*. EV, 2way-ANOVA with Bonferroni post-tests, n=3 per cell line). Notably, the morphology of DLC1v1+ cells was more pyramidal-shaped, epithelial and spread-out than the one of EV controls.

Accordingly, we next conducted IF microscopy to better visualize DLC1-driven spreading morphologies (**S6b**). Cells (AGS, N87) were transfected as above, followed by fixation and staining using DLC1 or FLAG Abs and phalloidin for visualisation of the actin cytoskeleton. Numbers of adherent cells were counted, normalized to total cell counts and calculated as means ± S.E. (*p<0.05 *vs*. EV, 2way-ANOVA with Bonferroni post-tests, n=3 per cell line). As before, DLC1+ cells, both v1 and v4, were flattened and spread-out with more focal adhesions (v1>v4) and neurite-like extensions (v4>v1) [6]. Similar results were obtained for N87 cells **(S7).**

Since cytoskeleton rearrangements are also connected to cell motility, we explored the role of DLC1 in cell migration (**S8a**) and matrix invasion (**S8b**). Cells were transfected as above, followed by re-seeding into wide-pore trans-well inserts for 16 h before fixation and staining with crystal violet. Numbers of cells which migrated to the opposite side of the plastic insert were counted, normalized to total cell counts and calculated as means ± S.E. (*p<0.05 *vs*. EV, Kruskal-Wallis test with Dunn post-test, n=3 per cell line). Here, DLC1+ cells exhibited a lower propensity to migrate than controls. To investigate matrix invasion, cells were transfected as before, followed by re-seeding into MatriGel™-coated trans-well inserts for 16 h before fixation and staining with crystal violet. Numbers of invading cells were counted, normalized to total cell counts and calculated as means ± S.E. (*p<0.05 *vs*. EV, Kruskal-Wallis test with Dunn post-test, n=3 per cell line). As for the overall motility, invasion into matrices was reduced by both DLC1 isoforms.

In contrast to its role in regulation of cytoskeleton-dependent cell phenotypes, the impact of DLC1 on cell proliferation is less clear. Therefore, cells were transfected as above for the times indicated, and cell viability measured by colorimetric MTT assay (**S8c**). O.D. values were calculated as -fold ± S.E. compared with day 0 (n.s., 2way-ANOVA with Bonferroni post-tests, n=3 per cell line). However, after 4-6 days no difference in growth rates between DLC1+ and control cells could be recorded.

Conclusively, the data showed that DLC1v1 and v4 are both able to actively reshape the actin cytoskeleton towards adhesion at the expense of cell motility, emphasizing the hypothesis that DLC1v4 is a potentially oncogenic variant of FL DLC1v1 which is retained in GC cells.

**Expression and localization of DLC1 in the murine healthy stomach and GC**

*Dlc1^gt/+^* mice contain a gene trap insertion between exon 1/2 of the isoform 2 transcript resulting in a loss of *Dlc1v2* mRNA but not of other transcripts, while homozygosity was lethal [7-9]. To verify the genotype, whole tissue lysates were extracted from frozen livers of WT and *Dlc1^gt/+^* mice (n=5 per genotype). Murine DLC1 (123 kDa) protein was visualised using an Ab specific for the C-terminus. Western blot analyses confirmed loss of DLC1 protein in heterozygous gene trapped mice compared to WT littermates (*p<0.05 *vs.* WT, t-test, n=5 per genotype) **(S11a)**.

Likewise, gastric DLC1 positivity was reduced in stomachs from *Dlc1^gt/+^* mice. FFPE sections from stomachs collected from WT and *Dlc1^gt/+^* mice were stained using DLC1 Ab for IHC **(S11b)** and IF **(S11c)** microscopy. Numbers of DLC1+ cells per area (mm^2^) were counted, normalized to total nuclei counts and calculated as means ± S.E. Quantitative analyses of corpus and antrum regions corroborated loss of DLC1 in gene trapped mice (IF: *p<0.05 *vs.* WT, 2way-ANOVA with Bonferroni post-tests, IF: n=6 mice per genotype; IHC: n=3 mice).

Referring to the above collected evidence that DLC1 was present in ECL cells [4], we were intrigued to explore if expression of gastrointestinal peptid hormones [10] is altered in *Dlc1^gt/+^* mice **(S12)**. Total RNA from stomach and ileum tissues of WT and *Dlc1^gt/+^* mouse stomachs was extracted and subjected to RT-qPCR (*p<0.05 *vs.* WT, t-test, n=3 mice per genotype). Notably, mRNA levels for marker enzymes [11] of ECL cells, namely histidine decarboxylase (*Hdc*) and chromogranin A (*Chga*) were reduced by 30-40 %, ghrelin (*Ghrl*) and somatostatin (*Sst*) were decreased by 60 % in gene trapped mice compared with WT littermates. In contrast, mRNAs encoding for the parietal cell marker H^+^K^+^ATPase (*Atp4*) and the chief cell marker pepsinogen C (*Pgc*) were not diminished. These data were consistent with the previously stated predominant localization of DLC1 in ECL cells.

To study the so far unknown phenotype of gene trapped stomachs, FFPE sections from WT and *Dlc1^gt/+^* mouse stomachs (n=3 per genotype) were stained for IHC. Histopathological microscopic analyses of 12 months-old animals evinced active gastritis with increased submucosal and intramucosal infiltration of inflammatory leukocytes with rare cases of small adenomas, mainly in the antrum region of the lower part of the stomach. Supportive analyses confirmed increased cell proliferation and infiltration of macrophages and lymphocytes in stomachs from *Dlc1^gt/+^* mice. FFPE sections from WT and *Dlc1^gt/+^* mouse stomachs (n=3 per genotype) were stained using Ki67 (**S13a)**, F4/80 (**S13b)** and CD3 (**S13c)** Abs. Numbers of Ab+ cells per area (mm^2^) were counted and calculated as means ± S.E. (*p<0.05 *vs.* WT, 2way-ANOVA with Bonferroni post-tests, n=3 mice per genotype). Quantitative analyses revealed low macrophage infiltration in the corpus compared with high F4/80 staining in the antrum crypts. Likewise, Ki67 positivity was 2 to 3-fold stronger in the corpus and antrum of *Dlc1^gt/+^* mice as compared with WT mice.

In sum, these staining patterns were consistent with the above evinced morphological phenotypes that hypomorphism of one *Dlc1* allele facilitates transition from proliferation and inflammation towards malignancy.

This conclusion was supported by examination of proinflammatory cytokines and CD marker molecules on subpopulations from immune cells (**S14a)**. Total RNA from stomach and ileum tissues of WT and *Dlc1^gt/+^* mouse stomachs (n=3 mice per genotype) was subjected to RT-qPCR (n.s. *vs.* WT, t-test, n=3 per genotype). *Dlc1^gt/+^* mice exhibited a trend for more gastric mRNA encoding for the T-helper cell marker *Cd4* and the cytotoxic T-cell marker *Cd8* than WT mice. Accordingly, *Nos2* encoding for the inducible nitric oxide (NO) synthase enzyme and the proinflammatory cytokine *Ifnγ* were augmented. Supportive Western blot analyses (**S14b**) evinced increased inflammatory signalling in stomachs from *Dlc1^gt/+^* mice. Total tissue lysates were extracted from frozen livers of WT and *Dlc1^gt/+^* mice (n=5 per genotype). Murine general and phosphorylated STAT3 and JNK1 (SAPK1, p46/p54) proteins were visualised. Quantitative analyses confirmed 2-fold elevated phosphorylation of JNK1 in *Dlc1^gt/+^* stomachs (*p<0.05 *vs.* WT, 2way-ANOVA with Bonferroni post-tests, n=5 per group). Conclusively, these findings emphasize that loss of DLC1 promotes gastritis-dependent phenotypes *in vivo*.

**References**

1 Rhodes DR, Yu J, Shanker K, Deshpande N, Varambally R, Ghosh D *et al*. ONCOMINE: a cancer microarray database and integrated data-mining platform. *Neoplasia* 2004; 6: 1-6.

2 Cancer Genome Atlas Research N. Comprehensive molecular characterization of gastric adenocarcinoma. *Nature* 2014; 513: 202-209.

3 Cerami E, Gao J, Dogrusoz U, Gross BE, Sumer SO, Aksoy BA *et al*. The cBio cancer genomics portal: an open platform for exploring multidimensional cancer genomics data. *Cancer Discov* 2012; 2: 401-404.

4 Waldum HL, Rehfeld JF. Gastric cancer and gastrin: on the interaction of Helicobacter pylori gastritis and acid inhibitory induced hypergastrinemia. *Scand J Gastroenterol* 2019; 54: 1118-1123.

5 Braun AC, Olayioye MA. Rho regulation: DLC proteins in space and time. *Cell Signal* 2015; 27: 1643-1651.

6 Kim TY, Healy KD, Der CJ, Sciaky N, Bang YJ, Juliano RL. Effects of structure of Rho GTPase-activating protein DLC-1 on cell morphology and migration. *J Biol Chem* 2008; 283: 32762-32770.

7 Sabbir MG, Wigle N, Loewen S, Gu Y, Buse C, Hicks GG *et al*. Identification and characterization of Dlc1 isoforms in the mouse and study of the biological function of a single gene trapped isoform. *BMC Biol* 2010; 8: 17.

8 Sabbir MG, Prieditis H, Ravinsky E, Mowat MR. The role of Dlc1 isoform 2 in K-Ras2(G12D) induced thymic cancer. *PLoS One* 2012; 7: e40302.

9 Sabbir MG, Dillon R, Mowat MR. Dlc1 interaction with non-muscle myosin heavy chain II-A (Myh9) and Rac1 activation. *Biol Open* 2016; 5: 452-460.

10 Gribble FM, Reimann F. Function and mechanisms of enteroendocrine cells and gut hormones in metabolism. *Nat Rev Endocrinol* 2019; 15: 226-237.

11 Schubert ML, Rehfeld JF. Gastric Peptides-Gastrin and Somatostatin. *Compr Physiol* 2019; 10: 197-228.
