# Supplementary material for: Functional antagonism between CagA and DLC1 in gastric cancer
Source: Cell Death Discov. 2022 Aug 13;8:358. doi: 10.1038/s41420-022-01134-x (PMC9376073; doi:10.1038/s41420-022-01134-x)
Supplement: Supplementary file 2 — Supplementary Tables [file 41420_2022_1134_MOESM2_ESM.docx]

**Supplementary Tables**

| **Table S1 Antibodies** | | |
| --- | --- | --- |
| **Name** | **Company** | **Cat. No.** |
| β-Actin | Sigma Aldrich/Merck | A1978 |
| H+K+ATPase (β Subunit) | Sigma Aldrich/Merck | A274(2G11) |
| Chromogranin A | Santa Cruz Bio. | sc-13090 |
| DLC1(C) | Abcam | ab180697 |
| DLC1(N) | Thermo Fisher Scientific | PA5-18290 |
| F4/80 | Thermo Fisher Scientific | MF48000 |
| FLAG | Abcam | ab8112 |
| FLAG | Sigma Aldrich/Merck | F1804 |
| GFP | Roche Diagnostics | 11814460001 |
| Ki67 | BD Biosciences | 550609 |
| Ki67 | Novus | NB600-1252 |
| MIST1 | Cell Signaling | 14896 |
| p38 MAPK | Cell Signaling | 9212 |
| Phospho-p38 | Cell Signaling | 4511 |
| p44/42 MAPK (ERK1/2) | Cell Signaling | 4695 |
| Phospho-p44/42 | Cell Signaling | 9101 |
| p46/54 JNK1(SAPK1) | Cell Signaling | 9926 |
| Phospho-JNK1 | Cell Signaling | 9910 |
| STAT1 | Cell Signaling | 9939 |
| Phospho-STAT1 | Cell Signaling | 9914 |
| RhoA | Santa Cruz Bio. | sc-418 |
| CagA | Santa Cruz Bio. | sc-25766 |

| **Table S2 Primers** | | |
| --- | --- | --- |
| **Oligonucleotide** | **Sequence (5‘ -> 3‘)** | **Amplicon (bp)** |
| **RT-qPCR (mouse)** |  |  |
| 5-*Sst* | gagcccaaccagacagagaa | 150 |
| 3-*Sst* | gaagttcttgcagccagctt |  |
| 5-*Chga* | ccaatacccaatcaccaacc | 148 |
| 3-*Chga* | acagcctcctcttcctcctc |  |
| 5-*Hdc* | ctcatcccggctactatcca | 118 |
| 3-*Hdc* | caaggttagcagcctcttgg |  |
| 5-*Tph* | catcagccgagaacagttga | 184 |
| 3-*Tph* | ttcggatccatacaacagca |  |
| 5-*Ghrl* | ccatctgcagtttgctgcta | 178 |
| 3-*Ghrl* | gcttgtcctctgtcctctgg |  |
| 5-*Gif* | cttggccctgacctgtatgt | 191 |
| 3-*Gif* | taggttgctcaggtgtcacg |  |
| 5-*Pgc* | ccaacctgtgggtgtcttct | 187 |
| 3-*Pgc* | ttagggacctggatgctttg |  |
| 5-*Atp4a* | gttcctgatgctgtgctcaa | 118 |
| 3-*Atp4a* | tgccctctgagatgataccc |  |
| 5-*Foxp3* | ttcatgcatcagctctccac | 185 |
| 3-*Foxp3* | ctggacacccattccagact |  |
| 5-*Cd4* | aggaagtgaacctggtggtg | 107 |
| 3-*Cd4* | ctcctgcttcagggtcagtc |  |
| 5-*Cd8* | tatggcttcatcccacaaca | 190 |
| 3-*Cd8* | gactggcacgacagaaCtga |  |
| 5-*Nos2* | caccttggagttcacccagt | 170 |
| 3-*Nos2* | accactcgtacttgggatgc |  |
| 5-*Arg1* | aaagctggtctgctggaaaa | 122 |
| 3-*Arg1* | acagaccgtgggttcttcac |  |
| 5-*Ifng* | GCGTCATTGAATCACACCTG | 129 |
| 3-*Ifng* | TGAGCTCATTGAATGCTTGG |  |
| 5-*Gata3* | ccgaaaccggaagatgtcta | 131 |
| 3-*Gata3* | agatgtggctcagggatgac |  |
| 5-*Rorc* | tgcaagactcatcgacaagg | 177 |
| 3-*Rorc* | aggggattcaaCatcagtgc |  |
| 5-*Dlc1(N)* | ggggaagagcggtttctatc | 188 |
| 3-*Dlc1(N)* | tgcatggtggacagtgtctt |  |
| 5-*Dlc1(C)* | cgtattgaggacctggagga | 293 |
| 3-*Dlc1(C)* | tcgtgtccttgctttcagtg |  |
| **RT-qPCR (human)** |  |  |
| 5-*DLC1(N)* | ctttctctggaagccagcac | 213 |
| 3-*DLC1(N)* | accagctattccccaggagt |  |
| 5-*DLC1(C)* | ccctcactctggaagcactc | 268 |
| 3-*DLC1(C)* | tcccagaggtgctgttcttt |  |
| **Cloning (human gene)** |  |  |
| 5-BamHI-DLC1v1(FL) | at *GGATCC* **atg**tctgtagctatcagaaagagaag | 4586 |
| 3-NotI-DLC1v1(FL) | at *GCGGCCGC* **tca**cctagatttggtgtctttggtttcag |  |
| 5-BamHI-DLC1v4(ΔSAM) | at *GGATCC* **atg**aagctagaaattagtcctcatc | 3053 |
| 3-NotI-DLC1v4(ΔSAM) | at *GCGGCCGC* **tca**cctagatttggtgtctttggtttcag |  |
| **Cloning (human promoter)** |  |  |
| 5-KpnI-*pDLC1v1* | at *GGTACC* ccatattctaacagaaatatgcaaac | 1148 |
| 3-SacI-*pDLC1v1* | at *GAGCTC* gtcatcatagtttaacaacagacaga |  |
| 5-KpnI-*pDLC1v4* | at *GGTACC* aagtgctccttccagccatatctt | 658 |
| 3-HindIII-*pDLC1v4* | at *AAGCTT* ccgctcgcagacgccttcagc |  |

| **Table S3 *DLC1* mRNA up-regulation in upper GI cancers**  Data were retrieved from *Oncomine*. Legend: N/n patient case numbers; BE Barrett esophagus; DIF diffuse type;  IM intestinal metaplasia; NT non-tumour (normal gastric tissue); TU tumour. | | | | | | |
| --- | --- | --- | --- | --- | --- | --- |
| **data set** | **N** | **NT** | **n** | **TU** | **n** | **mRNA** |
| **Chen**  **Gastric** | 29 | **Normal Stomach** | 18 | **Diffuse Gastric**  **Adenocarcinoma** | 11 | Up-regulation in TU  *p=0.003 |
| **Cho**  **Gastric** | 50 | **Normal Stomach** | 19 | **Diffuse Gastric**  **Adenocarcinoma** | 31 | Up-regulation in TU  *p=0.028 |
| **DErrico**  **Gastric** | 37 | **Normal Stomach** | 31 | **Diffuse Gastric**  **Adenocarcinoma** | 6 | Up-regulation in TU  p=0.045 |
| **Wang**  **Gastric** | 27 | **Normal Stomach** | 15 | **Gastric**  **Adenocarcinoma** | 12 | Up-regulation in TU  *p=0.044 |
| **data set** | **N** | **TU** | **n** | **TU** | **n** | **mRNA** |
| **Chen**  **Gastric** | 71 | **Intestinal Gastric**  **Adenocarcinoma** | 60 | **Diffuse Gastric**  **Adenocarcinoma** | 11 | Up-regulation in DIF  *p=0.011 |
| **DErrico**  **Gastric** | 32 | **Intestinal Gastric**  **Adenocarcinoma** | 26 | **Diffuse Gastric**  **Adenocarcinoma** | 6 | Up-regulation in DIF  *p=0.036 |
| **Forster**  **Gastric** | 43 | **Gastric**  **Adenocarcinoma** | 24 | **Diffuse Gastric**  **Adenocarcinoma** | 19 | Up-regulation in DIF  *p=3.37*e-6 |
| **Ooi**  **Gastric 2** | 27 | **Intestinal Gastric**  **Adenocarcinoma** | 21 | **Diffuse Gastric**  **Adenocarcinoma** | 6 | Up-regulation in DIF  *p=0.020 |
| **Ooi**  **Gastric** | 101 | **Intestinal Gastric**  **Adenocarcinoma** | 54 | **Diffuse Gastric**  **Adenocarcinoma** | 47 | Up-regulation in DIF  *p=8.49*e-4 |
| **data set** | **N** | **TU** | **n** | **IM/TU** | **n** | **mRNA** |
| **Hao**  **Esophagus** | 28 | **Normal**  **Esophagus** | 14 | **Barrett**  **Esophagus** | 14 | Up-regulation in BE  *p=0.003 |
| **Kim**  **Esophagus** | 43 | **Normal**  **Esophagus** | 28 | **Barrett**  **Esophagus** | 15 | Up-regulation in BE  *p=2.45*e-10 |
| **Kim**  **Esophagus** | 103 | **Normal**  **Esophagus** | 28 | **Esophageal**  **Adenocarcinoma** | 75 | Up-regulation in TU  *p=7.95*e-20 |

| **Table S4 Overview of *DLC1* pathway gene alterations in human cancers**  Data were retrieved from *cBioPortal*. All tumours were included in the analysis. Legend: a = Amplification, b = Deep deletion, c = missense/truncation mutation, d = mRNA up-regulation, e = mRNA down-regulation. N = number of patient cases. | | | | | | |
| --- | --- | --- | --- | --- | --- | --- |
| **Tumour data set** | **Cases altered** | **Type and number of alterations (N)** | | | | |
|  |  | **a** | **b** | **c** | **d** | **e** |
| **Gastric Adenocarcinoma (TCGA, Nature)** | | | | | | |
| *DLC1* | 56 of 295 (19 %) | **7** | 7 | **28** | **14** | 0 |
| *CAV1* | 28 of 295 (10 %) | **11** | 0 | **4** | **13** | 0 |
| *RHOA* | 36 of 295 (12 %) | **0** | 7 | **16** | **13** | 0 |
| *SRC* | 44 of 295 (15 %) | **8** | 0 | **3** | **33** | 0 |
| *ROCK1* | 38 of 295 (13 %) | **7** | 0 | **16** | **15** | 0 |
| *ROCK2* | 30 of 295 (10 %) | **4** | 1 | **11** | **14** | 0 |
| *MAPK8 [SAPK1]* | 23 of 295 (8 %) | **1** | 3 | **5** | **14** | 0 |
| **Gastric Adenocarcinoma (TCGA, PanCancerAtlas)** | | | | | | |
| *DLC1* | 78 of 440 (18 %) | **17** | 7 | **34** | **13** | 7 |
| *CAV1* | 38 of 440 (9 %) | **11** | 1 | **4** | **19** | 3 |
| *RHOA* | 48 of 440 (11 %) | **0** | 7 | **24** | **10** | 7 |
| *SRC* | 32 of 440 (7 %) | **11** | 0 | **5** | **7** | 9 |
| *ROCK1* | 54 of 440 (12 %) | **8** | 0 | **27** | **12** | 7 |
| *ROCK2* | 42 of 440 (10 %) | **4** | 0 | **20** | **7** | 11 |
| *MAPK8 [SAPK1]* | 27 of 440 (6 %) | **1** | 2 | **5** | **7** | 12 |
| **Gastric Adenocarcinoma (TCGA, Firehose Legacy)** | | | | | | |
| *DLC1* | 66 of 478 (14 %) | **18** | 8 | **31** | **9** | 0 |
| *CAV1* | 53 of 478 (11 %) | **12** | 1 | **25** | **15** | 0 |
| *RHOA* | 49 of 478 (10 %) | **1** | 9 | **19** | **12** | 8 |
| *SRC* | 97 of 478 (20 %) | **15** | 0 | **15** | **67** | 0 |
| *ROCK1* | 55 of 478 (12 %) | **12** | 0 | **24** | **19** | 0 |
| *ROCK2* | 38 of 478 (8 %) | **6** | 1 | **10** | **21** | 0 |
| *MAPK8 [SAPK1]* | 27 of 478 (6 %) | **1** | 3 | **6** | **16** | 1 |
|  | | | | | | |

| **Table S5 Correlation of *DLC1* pathway gene alterations with prognosis in GC**  Data and Kaplan-Meier analyses were retrieved from *cBioPortal.* Legend: cases with alterations (“ALT“); cases without alterations (“WT”); not assessable (“NA”); OS overall survival; PFS progression-free survival; DSS disease-specific survival; DFS disease-free survival. | | | | | |
| --- | --- | --- | --- | --- | --- |
| **gene(s)** | **status** | **total cases** | **cases deceased** | **median month**  **survival** | **log-rank test**  **p-value** |
| *[Chr8: DLC1]* | | | | | |
| **GC_PanCA** | | | | | |
| **OS** | ALT | 61 | 24 | 28.96 | 0.720 |
|  | WT | 374 | 144 | 30.90 |  |
| **PFS** | ALT | 62 | 19 | 55.10 | 0.365 |
|  | WT | 375 | 123 | 42.31 |  |
| **GC_TCGA FHL** | | | | | |
| **OS** | ALT | 56 | 19 | NA | 0.630 |
|  | WT | 379 | 151 | 28.71 |  |
| **DFS** | ALT | 45 | 11 | 45.20 | 0.206 |
|  | WT | 297 | 104 | 42.28 |  |
| **GC_NAT** | | | | | |
| **OS** | ALT | 37 | 7 | 27.93 | 0.852 |
|  | WT | 251 | 48 | 59.01 |  |
| **DFS** | ALT | 22 | 3 | NA | 0.578 |
|  | WT | 140 | 28 | 55.06 |  |
| **TCGA PanCancer Altlas Studies (n=32)** | | | | | |
| **OS** | ALT | 662 | 202 | 88.86 | 0.216 |
|  | WT | 10143 | 3312 | 78.41 |  |
| **DSS** | ALT | 638 | 131 | 202.65 | **0.0834** |
|  | WT | 9622 | 2304 | 133.74 |  |
| **EC/GC Studies (n=17)** | | | | | |
| **OS** | ALT | 246 | 81 | 31.54 | **0.0899** |
|  | WT | 2543 | 1052 | 30.90 |  |
| **PFS** | ALT | 71 | 25 | 45.24 | 0.459 |
|  | WT | 582 | 222 | 26.53 |  |
|  | | | | | |
| *[Chr8: DLC1] CAV1 SRC RHOA ROCK1 ROCK2 MAPK8* | | | | | |
| **TCGA PanCancer Altlas Studies (n=32)** | | | | | |
| **OS** | ALT | 1651 | 579 | 70.82 | **0.0518** |
|  | WT | 9153 | 2934 | 79.99 |  |
| **PFS** | ALT | 1647 | 618 | 57.76 | 0.426 |
|  | WT | 8967 | 3278 | 62.00 |  |
| **EC/GC Studies (n=17)** | | | | | |
| **OS** | ALT | 701 | 245 | 31.56 | **0.101** |
|  | WT | 2085 | 888 | 30.90 |  |
| **PFS** | ALT | 199 | 77 | 41.16 | 0.964 |
|  | WT | 451 | 168 | 27.12 |  |
| **GC_PanCA** | | | | | |
| **OS** | ALT | 247 | 90 | 34.29 | 0.198 |
|  | WT | 188 | 78 | 26.47 |  |
| **PFS** | ALT | 249 | 74 | 55.10 | **0.0723** |
|  | WT | 188 | 68 | 31.63 |  |
| **GC_TCGA FHL** | | | | | |
| **OS** | ALT | 177 | 63 | 28.71 | 0.874 |
|  | WT | 258 | 107 | 34.26 |  |
| **DFS** | ALT | 140 | 42 | NA | 0.771 |
|  | WT | 202 | 73 | 42.28 |  |
| **GC_NAT** | | | | | |
| **OS** | ALT | 104 | 20 | 27.93 | 0.838 |
|  | WT | 184 | 35 | 59.01 |  |
| **DFS** | ALT | 63 | 14 | NA | 0.255 |
|  | WT | 99 | 17 | 55.06 |  |
|  | | | | | |

| **Table S6 *DLC1* gene mutations in GI cancers**  Data were retrieved from *cBioPortal.* Legend: cases with alterations (“ALT“); cases without alterations (“WT”); not assessable (“NA”); $ localized N-terminal of SAM domain; & localized in or C-terminal of SAM domain.  Abbrev: CCLE cancer cell line encyclopedia; EC esophageal cancer (squamous cell carcinoma & adenocarcinoma); GC gastric cancer; FHL fire hose legacy; NAT nature; NCI National Cancer Institute; OncoSG Genome Institute of Singapore; UHK University of Hongkong. | | | | |
| --- | --- | --- | --- | --- |
| **gene(s)** | **Studies**  **(n)** | **Sample Number (N)** | **Cancer**  **Hotspot (**$) | **Splice**  **Variant (**&) |
| *[Chr8: DLC1]* | | | | |
| **Cell Lines [multiple]** | | | | |
| CCLE and NCI-60 | 3 | 2826 | **R351W** | X501,X438,X439_splice |
| **Tissues [multiple]** | | | | |
| PanCancer | 7 | 37748 | none | X450_splice |
| EC and GC | 17 | 3791 | **A350V** | X439_splice,R522= |
| **Tissues [single]** | | | | |
| **GC** | | | | |
| OncoSG | 1 | 147 | **A350V** | X439_splice |
| Pfizer and UHK | 1 | 100 | **A350V** | none |
| UHK | 1 | 22 | none | X439_splice |
| **EC** | | | | |
| FHL | 1 | 186 | none | R522= |
| NAT | 1 | 559 | none | R522= |
