# Supplementary material for: Functional antagonism between CagA and DLC1 in gastric cancer
Source: Cell Death Discov. 2022 Aug 13;8:358. doi: 10.1038/s41420-022-01134-x (PMC9376073; doi:10.1038/s41420-022-01134-x)
Supplement: Supplementary file 3 — Supplementary Figure Legends [file 41420_2022_1134_MOESM3_ESM.doc]

**Supplement Figure Legends**

**S1 Correlation of DLC1 protein expression to clinical factors of GC patients**

**A,** Down-regulation of DLC1 protein in GC grouped by Lauren histology. Sections from human FFPE normal/non-tumour stomach tissue (NT: n=30) and tumour cases (TU: n=116) were stained by IHC with DLC1 Ab specific for the N-terminus/SAM+ domain of the protein. Data are means ± S.E. of graded scores (*p<0.05 *vs.* NT, Kruskal-Wallis test with Dunn post-tests). Representative images (left) and quantitative analyses (right) [epithelium only; stroma not shown] are shown (NT: n=30; TU: n=34 diffuse, n=56 intestinal). Colour code: Brown = DLC1; Blue = nuclei (hematoxylin); Original magnifications 100×; Scale bar = 100 µm.

Legend: TU intestinal, differentiated: i) Score 0 (neg.), (ii) Score 1+, iii) Score 2+, iv) Score 3+ (stroma pos.); TU diffuse, undifferentiated: (v) Score 0 (neg.), (vi) Score 1+ (pos.).

**B,** Down-regulation of DLC1 protein by grade (G) of tumour dedifferentiation.

Cases (TU: n=14 G1; n=31 G2; n=62 G3).

**C,** by UICC stage.

Cases (TU: n=9 “0”; n=13 I; n=6 II; n=23 III; n=20 IV).

**D**, by tumour (T) size.

Cases (TU: n=11 T1; n=15 T2; n=58 T3; n=23 T4).

**E**, by nodal (N) status.

Cases (TU: n=32 N0; n=24 N1; n=10 N2; n=31 N3).

**F,** Up-regulation of DLC1 protein by metastatic (M) status.

Cases (TU: n=36 M0; n=11 M1) (*p<0.05 *vs.* NT, Mann-Whitney test).

**S2 *DLC1* gene alterations predict prognosis in GC patients**

**A**, *DLC1* mRNA in cancer data sets from Oncomine® (**Table S3**). Exemplary data are shown for [Forster Gastric: INT n=24 *vs.* n=19 DIF, *p=3.37e-6, t-test]. Legend: DIF = Diffuse GC; INT = Intestinal GC.

**B**, Association of *DLC1* mRNA with diffuse and CMS2 (GS) GC. Data were retrieved from cBioportal® of Cancer Genomics data sets [Gastric Adenocarcinoma, TCGA, PanCancer Atlas (n=440) and Nature (n=295)] and calculated as -fold ± S.E. compared to the mean of all diploid samples (*p<0.05, Mann-Whitney test or Kruskal-Wallis test with Dunn post-tests). Legend: MSI = microsatellite instable; GS = genomically stable; CIN = chromosomal instable; EBV = Epstein Barr Virus; DIPL = diploid (reference population).

**C**, Up-regulation of *DLC1* mRNA in diffuse GC. Data based on B. Legend: DIF = diffuse; INT = intestinal.

**D,** Up-regulation of *DLC1* mRNA by metastatic (M) status. Data based on B. Legend: M0 = no metastases, M1 = distant metastases present.

**E**, *DLC1* pathway gene alterations. Oncoprint® files were retrieved from cBioportal® of Cancer Genomics data set [Gastric Adenocarcinoma, TCGA, PanCancer Atlas (n=440)]: Left: Percent (%) altered cases compared with the total patient number; Right: Patients’ samples without gene alterations were marked in grey or cut off.

**F,** Association of *DLC1* pathway 7-gene alterations (*DLC1*, *CAV1, SRC, RHOA, ROCK1/2, JNK1*) with prognosis in GC patients. Kaplan-Meier survival analysis was performed based on the data set provided in [TCGA, PanCancer Atlas (n=32 studies)]. Log-Rank test is depicted in the graph (**Tables S4-5)**. Legend: OS = overall survival; WT = “wildtype” / unaltered genes; ALT = altered genes.

**S3 Expression and localization of DLC1 in the non-neoplastic stomach**

**A,** DLC1 protein is localized to gastric enterochromaffin-like (ECL) cells. Sections from human FFPE stomach tissue (n=3 cases) were stained with DLC1 and chromogranin A (CHGA) Abs for IF microscopy. Colour code: Green = DLC1; Red = CHGA; Blue = nuclei (DAPI). Original magnifications 400× (right: zoomed-in 630×); Scale bar = 100 µm. White arrows/frames: Yellow = zoomed-in overlay (DLC1+/CHGA+).

**B,** DLC1 protein is not colocalized with acid-secreting parietal cells. Sections from human FFPE stomach tissue (n=3 cases) were stained with DLC1 and H^+^K^+^ATPase Abs for IF microscopy. Colour code: Green = DLC1; Red = H^+^K^+^ATPase; Blue = nuclei (DAPI). Original magnifications 400× (right: merged DAPI image 630×); Scale bar = 100 µm.

**C,** Detection of DLC1 protein in murine gastric enterochromaffin-like (ECL), but not acid secreting parietal cells. FFPE sections from WT mouse stomachs were stained using DLC1 and chromogranin A or H^+^K^+^ATPase Abs for IF microscopy. Colour code: Green = DLC1; Red = CHGA or H^+^K^+^ATPase; Blue = nuclei (DAPI). Original magnifications 400× (left: zoomed-in 630×); Scale bar = 100 µm.

Representative images of the corpus regions (**A-C**) are shown.

**S4 Supportive images on DLC1 antagonisms on CagA+ AGS phenotypes**

Subconfluent cells (AGS) were transfected with EV, CagA or DLC1 expression plasmids or a combination thereof for 36 h, followed by fixation and staining using DLC1 and FLAG Abs for IF microscopy. Representative images are shown. Colour code: Red = DLC1; Green = CagA (GFP); Yellow = DLC1/CagA (overlay); Blue = nuclei (DAPI); Original magnifications 400× (zoomed-in 630×); Scale bar = 20 µm. White frames/arrows: DLC1 promotes neurite-like extensions (DLC1v4+) or localizes to focal adhesions (DLC1v1+) (abbrev. “fa”), membrane-bound (abbrev. “mb”) CagA forms needle-like elongations (“humming bird”).

**S5 Supportive images on DLC1 antagonisms on CagA+ HEK293T phenotypes**

Subconfluent cells (HEK293T) were transfected, fixed and stained as in **S4**.

**S6 DLC1 promotes adhesion and spreading morphologies of GC cells**

**A,** Adhesion. Subconfluent cells (AGS, HEK293T) were transfected with EV or DLC1v1/4 expression plasmids for 36 h, followed by re-seeding onto plastic culture dishes for the times indicated before fixation and staining with crystal violet. Quantitative analyses (left) and representative images (AGS: right) are shown. Numbers of adherent cells were counted, normalized to total cell counts and calculated as means ± S.E. (*p<0.05 *vs*. EV, 2way-ANOVA with Bonferroni post-tests, n=3 per cell line). Colour code: Violet = Adherent cells; Original magnifications 100×; Scale bar = 100 µm.

**B**, Spreading. AGS cells were transfected as in A, followed by fixation and staining using DLC1 and FLAG Abs for IF microscopy. Quantitative analyses (left) and representative images (right) are shown. Numbers of transfected (genotype+) and adherent (phenotype+) cells were counted, normalized to total cell counts and calculated as means ± S.E. (*p<0.05 *vs*. EV, 2way-ANOVA with Bonferroni post-tests, n=3 per cell line). Colour code: Red = DLC1; Green = actin (phalloidin); Blue = nuclei (DAPI); Original magnifications 400× (zoomed-in 630×); Scale bar = 20 µm. White frames/arrows: Cells with neurite-like extensions (DLC1v4+) and focal adhesions (DLC1v1+) spreading morphologies.

**S7 Supportive images on DLC1-mediated morphologies of GC cells**

Subconfluent cells (N87) were transfected with EV or DLC1v1/4 expression plasmids for 36 h,

followed by fixation and staining using DLC1 or FLAG Abs for IF microscopy. Quantitative analyses (left) and representative images (right) are shown. Numbers of transfected (genotype+) and adherent (phenotype+) cells were counted, normalized to total cell counts and calculated as means ± S.E. (*p<0.05 *vs*. EV, 2way-ANOVA with Bonferroni post-tests, n=3 per cell line). Colour code: Red = DLC1; Green = actin (phalloidin); Blue = nuclei (DAPI); Original magnifications 400× (zoomed-in 630×); Scale bar = 20 µm. White frames/arrows: Cells with neurite-like extensions and focal adhesion spreading morphologies.

**S8 DLC1 inhibits migration of GC cells**

**A,** Migration. Cells (AGS, HEK293T) were transfected as in **S6**, followed by re-seeding into trans-well inserts for 16 h before fixation and staining with crystal violet. Quantitative analyses (left) and representative images (right) are shown. Numbers of adherent cells were counted, normalized to total cell counts and calculated as means ± S.E. (*p<0.05 vs. EV, Kruskal-Wallis test with Dunn post-test, n=3 per cell line). Colour code: Violet = Migrated cells; Original magnifications 100×; Scale bar = 100 µm.

**B,** Invasion. Cells were transfected as in A, followed by re-seeding into MatriGel™-coated trans-well inserts for 16 h before fixation and staining with crystal violet. Quantitative analyses are shown. Numbers of invading cells were counted, normalized to total cell counts and calculated as means ± S.E. (*p<0.05 *vs*. EV, Kruskal-Wallis test with Dunn post-test, n=3 per cell line).

**C,** Proliferation. Cells were transfected as in A for the times indicated, and cell viability measured by colorimetric MTT assay. O.D. values were calculated as -fold ± S.E. compared with day 0 (n.s., 2way-ANOVA with Bonferroni post-tests, n=3 per cell line).

**S9 Supportive oncogenic signalling pathway analyses**

**A,** DLC1 augments phosphorylation of MAPKs. AGS cells were transfected with EV or DLC1v1 in absence or presence of GFP-CagA expression plasmid for 24 h, followed by serum deprival for additional 16 h, and restimulation with FCS (20 % *v/v*) or EGF (50 ng/ml) for the times indicated before extraction of total cell lysates. Quantitative analyses (top) and representative images (bottom) from Western blots (source file: “Original Western Blots” page 18-19). O.D. values from gels were normalized to HSP90 and presented as -fold ± S.E. (*p<0.05 *vs.* EV, 2way-ANOVA with Bonferroni post-tests, n=3 per cell line).

**B,** Gα protein activates, C3T inhibits CagA-mediated RHO-dependent transcription from a modified SRE derived of the human *c-FOS* promoter. Left: TsA201 (HEK293T equivalent) cells were transfected with EV, CagA or C3T (C3 toxin, *Clostridium botulinum*) expression plasmids or a combination thereof together with pSRE.L- or pRL-TK-luc reporters. Firefly luciferase was normalized to renilla luciferase and calculated as -fold ± S.E. (*p<0.05 *vs.* EV, 2way-ANOVA with with Bonferroni post-tests, n=6). Right: TsA201 cells were transfected with EV, DLCv1/4 or G13qL (constitutively active G-protein α subunit Gα13) expression plasmids or a combination thereof together with the reporters. Data are presented as above (*p<0.05 *vs.* EV, 2way-ANOVA with Bonferroni post-tests, n=2).

**C,** C3T inhibits G-protein-coupled RHOA activation by CagA and G13qL. Left: TsA201 cells were transfected with EV and increasing concentrations of CagA in presence or absence of C3T expression plasmid (source file: “Original Western Blots” page 20-22). Right: TsA201 cells were transfected with EV, G13qL or together with C3T (source file: page 23-24). Total cell lysates (Input) were subjected to RHOA pull-down assays. Representative images from Western Blots**.**

**S10 Supportive data on DLC1 colocalization with CagA**

HEK293T cells were transfected with CagA-FL (aa 1-1216), CagA-NT (1-877 aa), CagA-C (838-1216 aa) or CagA-CT (1029-1216 aa) expression constructs in combination with DLC1v1 or v4 plasmids, respectively. Quantitative analyses (top, left) and representative images (bottom) from proximity ligation assay (PLA) together with Western blots (top, right) confirming overexpression of DLC1v1 and v4 proteins (source file: “Original Western Blots” page 25). Numbers of red dots were counted, normalized to total nuclei counts and calculated as means ± S.E. (*p<0.05, 2way-ANOVA with Bonferroni post-tests, n=3 per plasmid). Colour code: Red dots = DLC1/CagA complex; Green = CagA (GFP); Blue = nuclei (DAPI); Original magnifications 630x; Scale bar = 10 µm. Note accumulation of DLC1+ PLA complexes in CagA-GFP+ cell areas (yellow = overlay).

**S11 Expression and localization of DLC1 in the non-neoplastic murine stomach**

**A,** Total DLC1 protein is reduced in *Dlc1^gt/+^* mice. Whole tissue lysates were extracted from frozen livers of WT and *Dlc1^gt/+^* mice (n=5 per genotype). Murine DLC1 (123 kDa) was visualised by an Ab specific for the C-terminus. Quantitative analysis (left) and representative images (right) from Western blots (source file: “Original Western Blots” page 26). O.D. values from gels were normalized to HSP90 and presented as -fold ± S.E. (*p<0.05 *vs.* WT, t-test, n=5 per group).

**B,** Gastric DLC1 positivity is reduced in stomachs from *Dlc1^gt/+^* mice. FFPE sections from WT and *Dlc1^gt/+^* mouse stomachs (n=6 per genotype) were stained using DLC1 Ab for IHC. Quantitative analyses (top) and representative images (bottom) of corpus and antrum. Numbers of DLC1+ cells per area (mm^2^) were counted and calculated as means ± S.E. (*p<0.05 *vs.* WT, 2way-ANOVA with Bonferroni post-tests, n=3 mice per genotype). Colour code: Brown = DLC1; Blue = nuclei (hematoxilin); Original magnifications 200×; Scale bar = 100 µm.

**C,** Gastric DLC1 positivity is reduced in stomachs from *Dlc1^gt/+^* mice. FFPE sections from WT and *Dlc1^gt/+^* mouse stomachs were stained using DLC1, CHGA and H^+^K^+^ATPase Abs for IF microscopy. Quantitative analyses (top) and representative images (bottom) of the corpus region. Numbers of DLC1+ cells per area (mm^2^) were counted, normalized to total nuclei counts and calculated as means ± S.E. (*p<0.05 *vs.* WT, 2way-ANOVA with Bonferroni post-tests, n=6 mice per genotype). Note the absence of overlay with parietal cells (DLC1/ H^+^K^+^ATPase). Colour code: Red = DLC1; Green = CHGA; Blue = nuclei (DAPI); Original magnifications 400×; Scale bar = 50 µm. White arrows/frames: Yellow = zoomed-in overlay (DLC1/CHGA).

**S12 Expression of ECL cell-related markers in the non-neoplastic murine stomach**

Gastric hormones are reduced in stomachs from *Dlc1^gt/+^* mice. Total RNA from stomach tissues of WT and *Dlc1^gt/+^* mouse stomachs (n=3 mice per genotype) was subjected to RT-qPCR. Ct-values were normalized to *B2m* and calculated as -fold ± S.E. (*p<0.05 *vs.* WT, t-test, n=3 per genotype).

**S13 Expression of immune-related markers in the non-neoplastic murine stomach**

Increased epithelial cell proliferation and leukocyte infiltration in stomachs from *Dlc1^gt/+^* mice. FFPE sections from WT and *Dlc1^gt/+^* mouse stomachs (n=3 per genotype) were stained by IHC using Ki67 **(A)**, F4/80 **(B)** or CD3 **(C)** Abs. Quantitative analyses (left) and representative images (right) of corpus and antrum. Numbers of DLC1+ cells per area (mm^2^) were counted and calculated as means ± S.E. (*p<0.05 *vs.* WT, 2way-ANOVA with Bonferroni post-tests, n=3 mice per genotype). Colour code: Brown = DLC1; Blue = nuclei (hematoxylin); Original magnifications 200×; Scale bar = 100 µm.

**S14 Expression of immune-related markers in the non-neoplastic murine stomach**

**A,** Increased inflammatory gene expression in stomachs from *Dlc1^gt/+^* mice. Total RNA from stomach tissues of WT and *Dlc1^gt/+^* mouse stomachs was subjected to RT-qPCR. Ct-values were normalized to *B2m* and calculated as -fold ± S.E. (n.s. *vs.* WT, t-test, n=3 per genotype).

**B,** Increased inflammatory signalling in stomachs from *Dlc1^gt/+^* mice. Whole tissue lysates were extracted from frozen livers of WT and *Dlc1^gt/+^* mice. General (un-phosphorylated) and phosphorylated proteins were visualised. Quantitative analyses (top) and representative images (bottom) from Western blots (source file: “Original Western Blots” page 27-28). O.D. values from gels were normalized to HSP90 and presented as -fold ± S.E. (*p<0.05 *vs.* WT, 2way-ANOVA with Bonferroni post-tests, n=5 per genotype).

**S15 Model of DLC1 and CagA cross-talk in the GC stem cell niche**

*H. pylori* down-regulates DLC1 expression (i) directly via binding of CagA to CAV1-rich membrane complexes at focal adhesions and cell junctions triggering RHOA-dependent malignant phenotypes (loss of polarity/adhesion, migration e.a.) and inflammation (NFκB/IL8 e.a.); and (ii) indirectly by targeting putative DLC1+/MIST1+ stem/chief cells which give rise to other gastric lineages including ECL cells, which showed the most prominent DLC1 positivity in uninfected individuals (humans, mice). *H. pylori* infection also alters the overall gastric hormone milieu (as marked by arrows), hampering H+ secretion by parietal cells and finally leading to a deterioration of organ function.
