# Supplementary figures and images for: Functional antagonism between CagA and DLC1 in gastric cancer
Source: Cell Death Discov. 2022 Aug 13;8:358. doi: 10.1038/s41420-022-01134-x (PMC9376073; doi:10.1038/s41420-022-01134-x)

IHC: DLC1

GC Patients

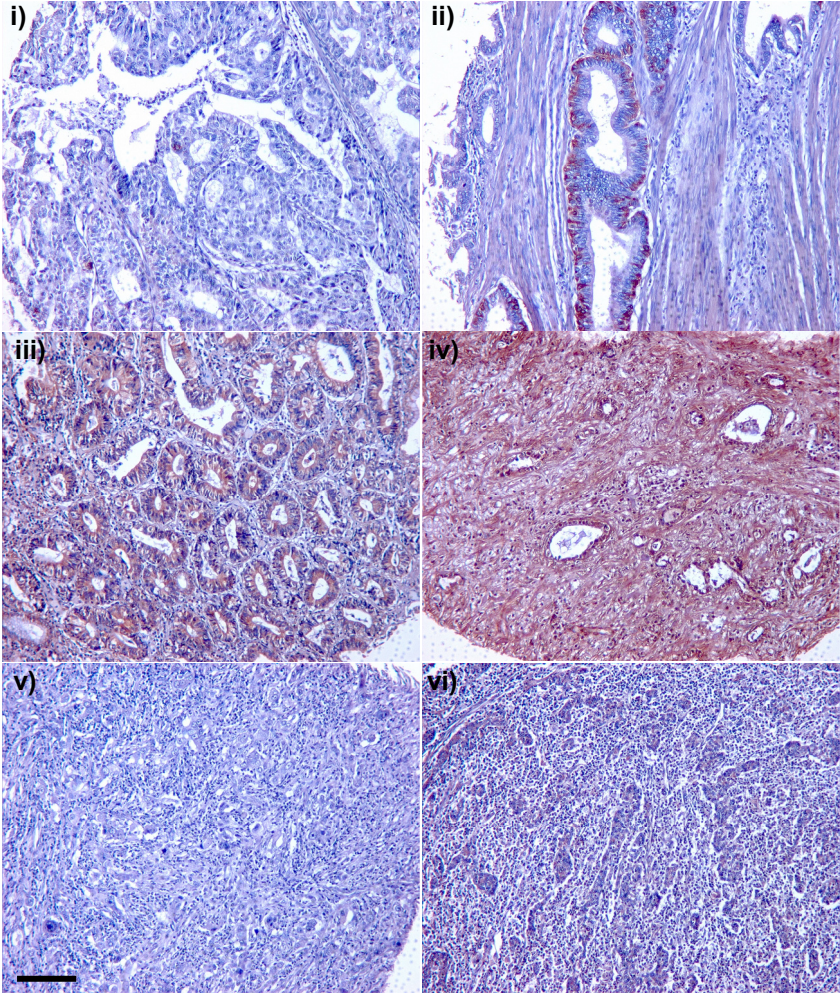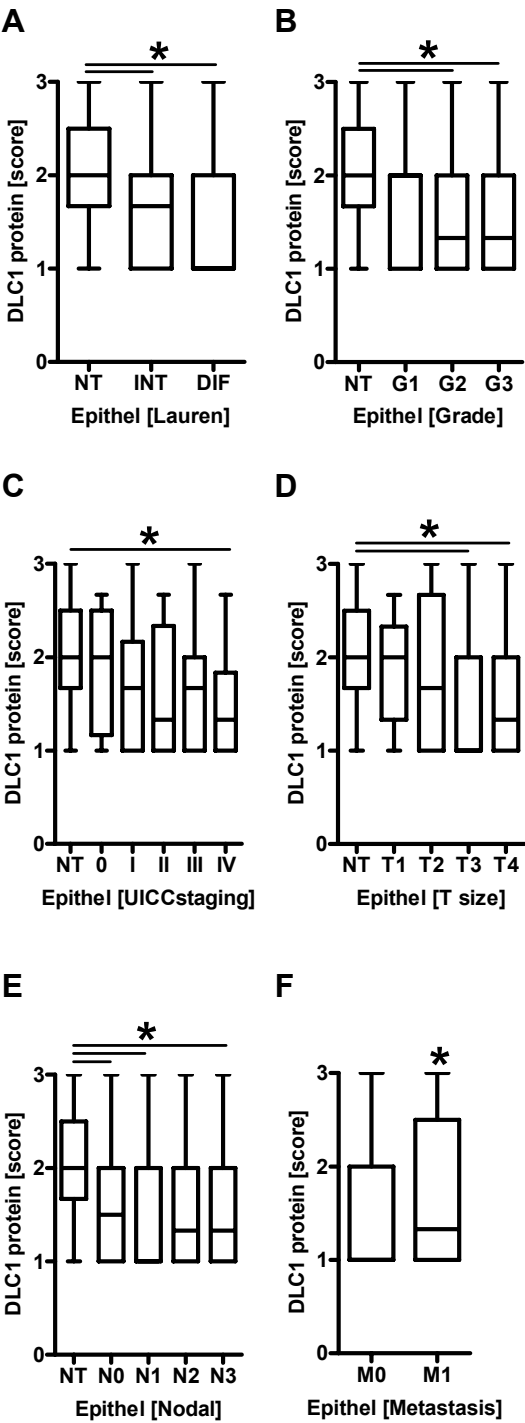

Supplement: Supplementary file 4 — Supplementary Figure S1 [file 41420_2022_1134_MOESM4_ESM.pdf]

A

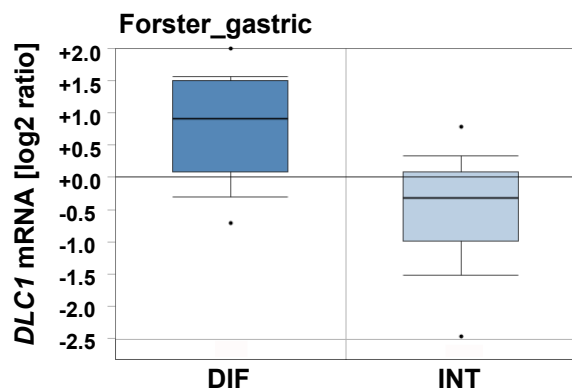

B

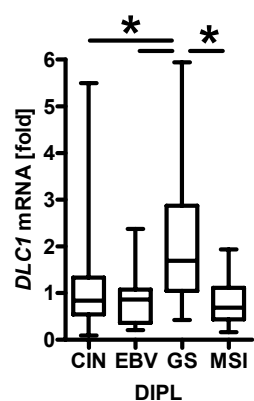

C

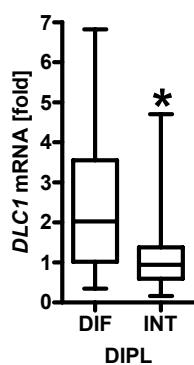

D

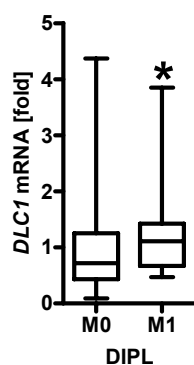

GC NAT&amp;PanCA

E

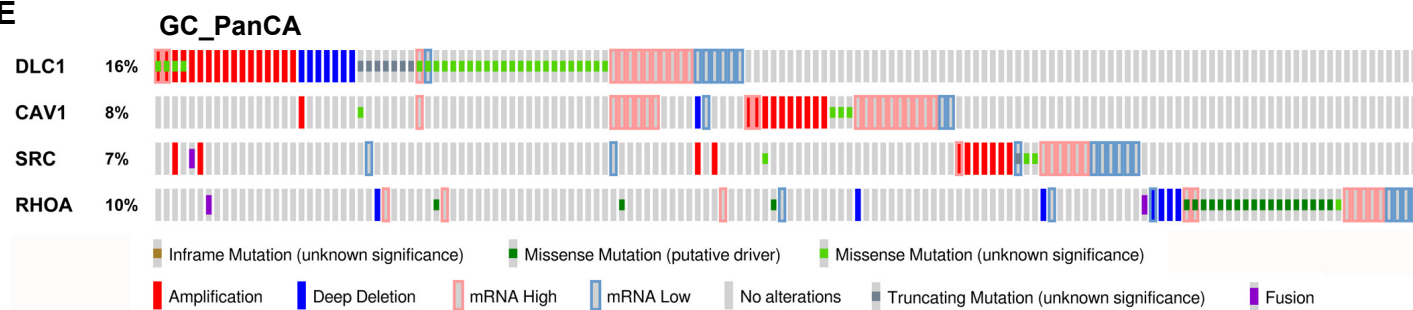

F

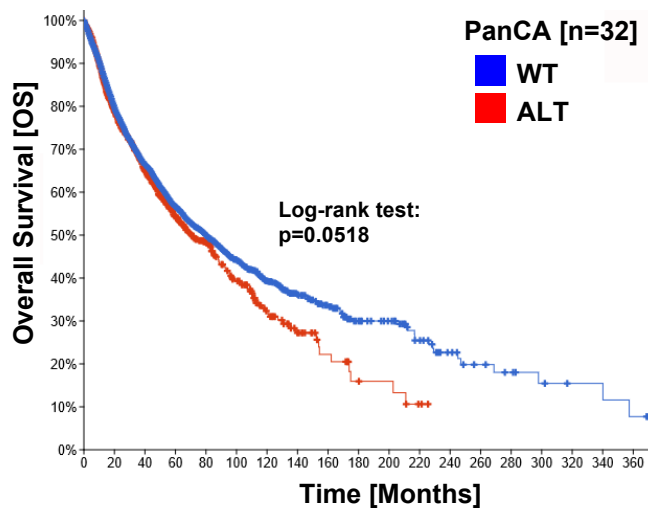

S2

Supplement: Supplementary file 5 — Supplementary Figure S2 [file 41420_2022_1134_MOESM5_ESM.pdf]

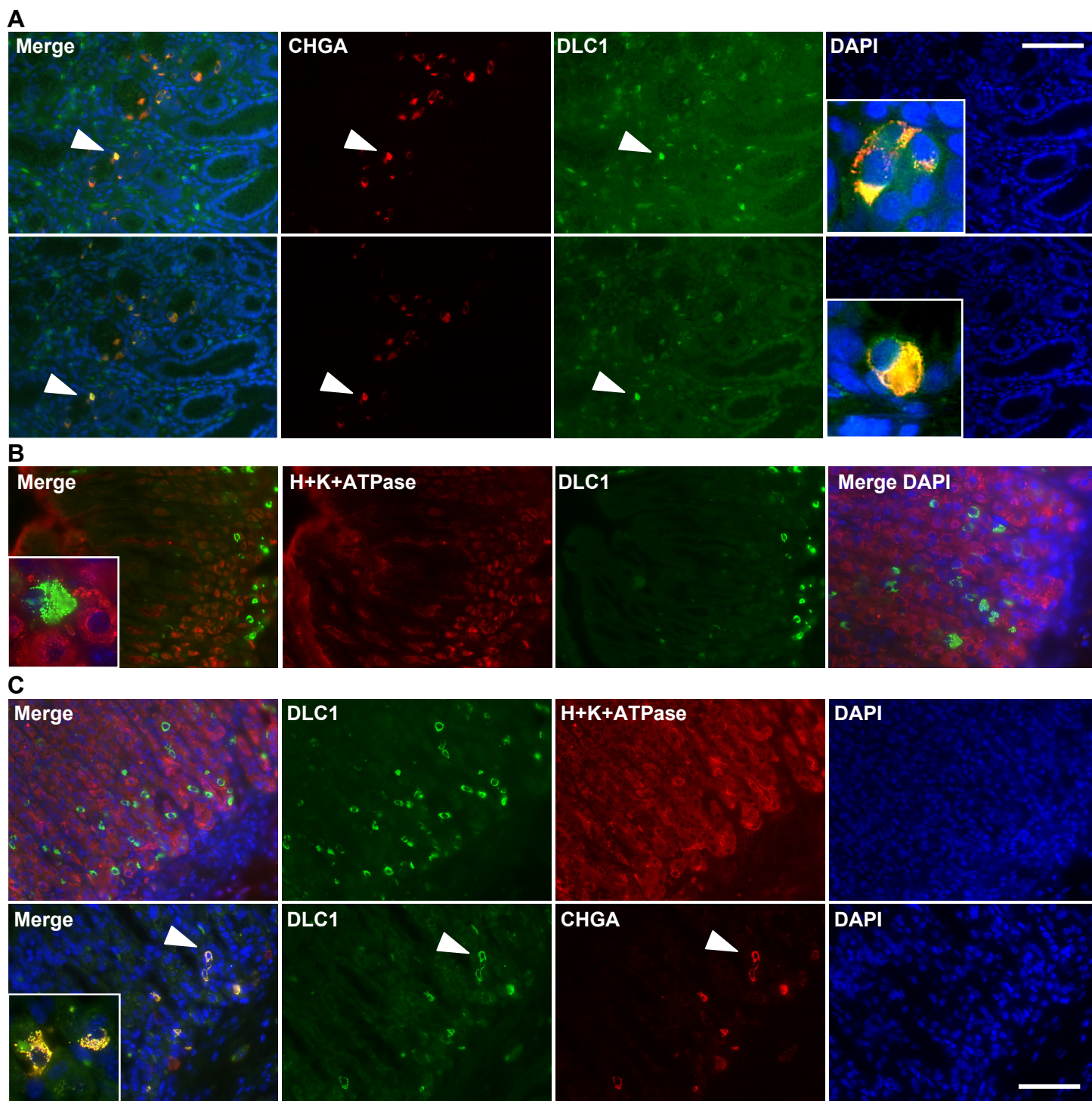

Supplement: Supplementary file 6 — Supplementary Figure S3 [file 41420_2022_1134_MOESM6_ESM.pdf]

**CagA-GFP + Flag-DLC1.v1**

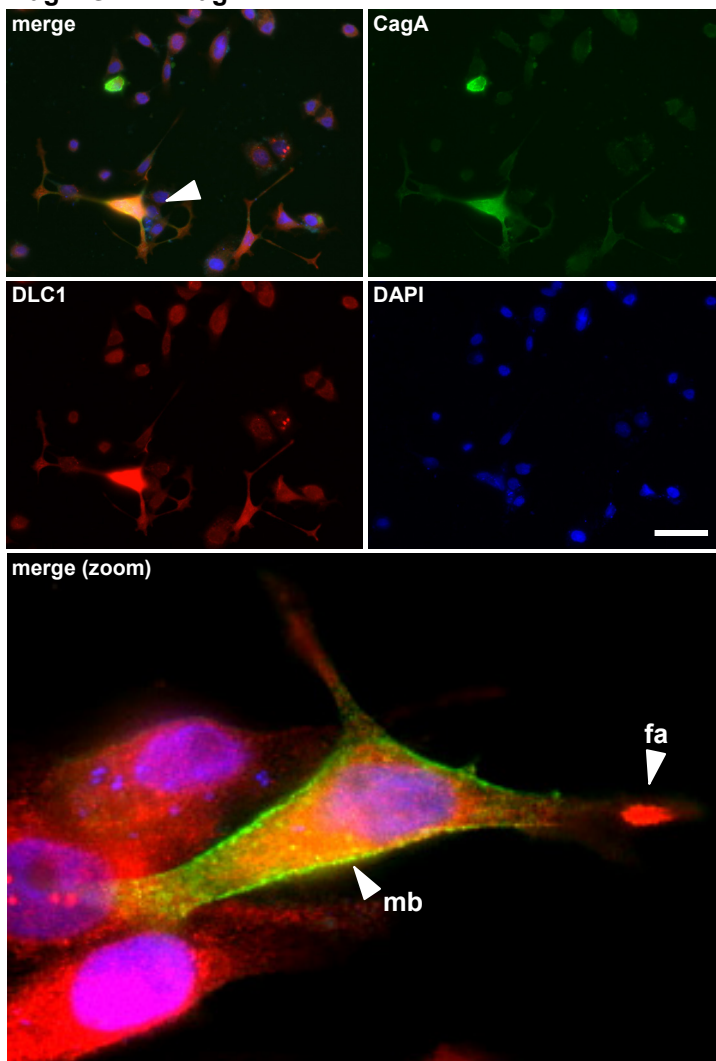

Supplement: Supplementary file 7 — Supplementary Figure S4 [file 41420_2022_1134_MOESM7_ESM.pdf]

EV+CagA

DLC1v.1+CagA

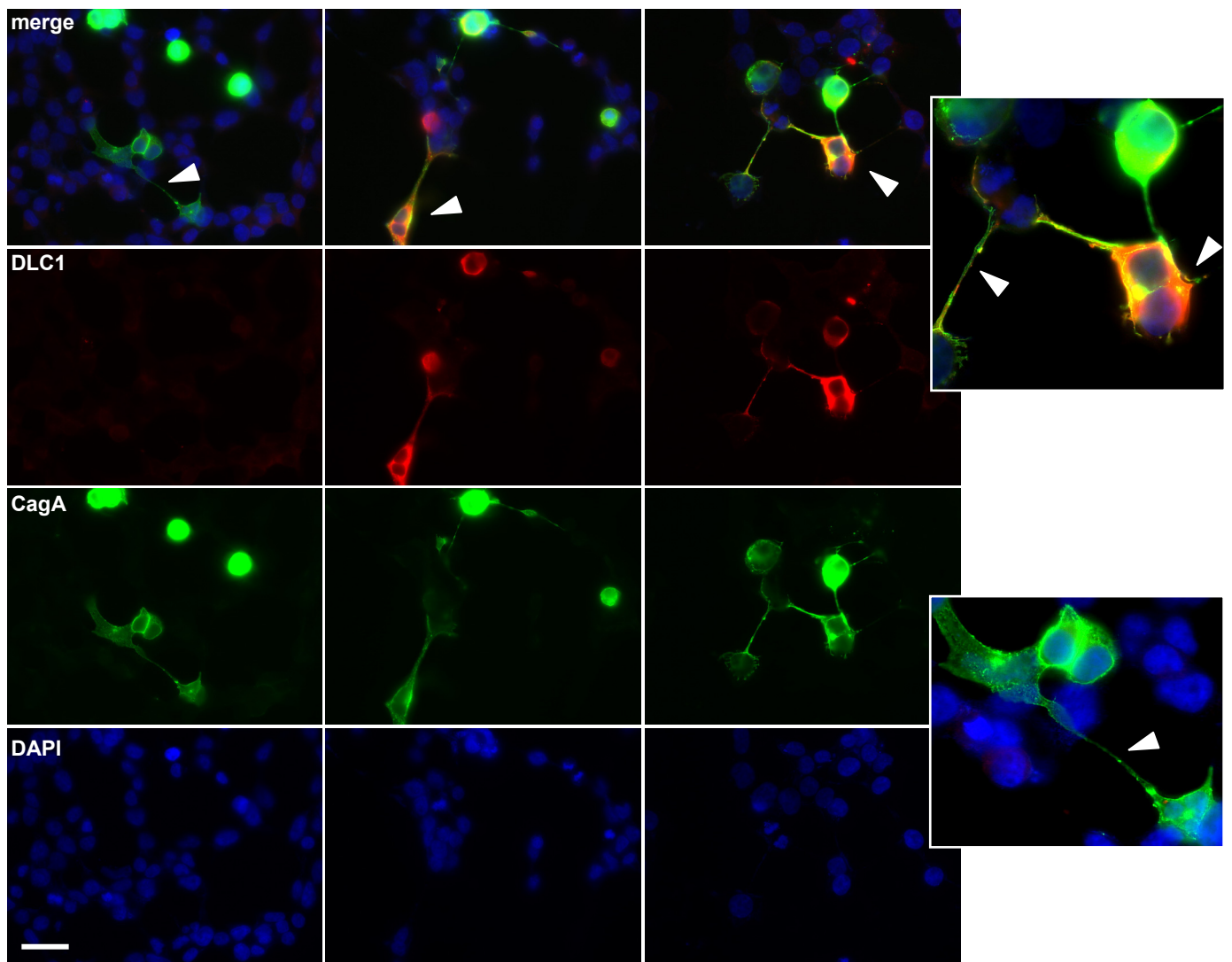

Supplement: Supplementary file 8 — Supplementary Figure S5 [file 41420_2022_1134_MOESM8_ESM.pdf]

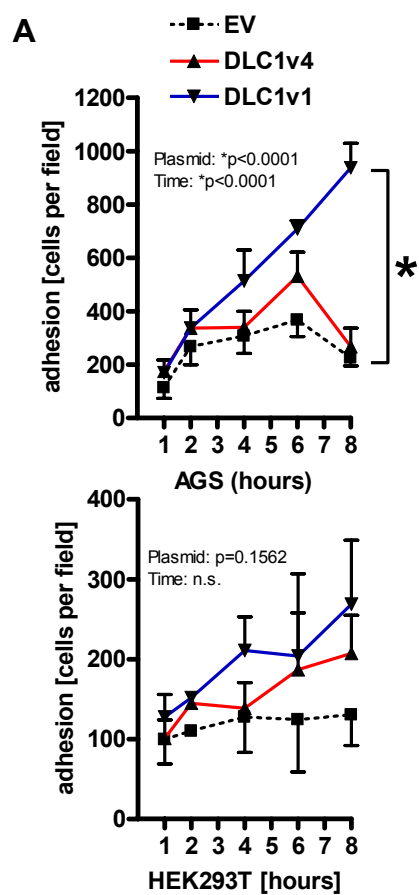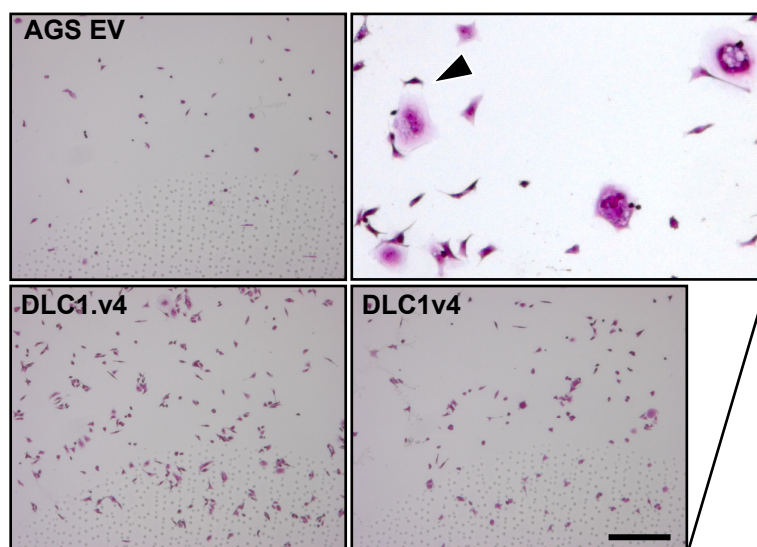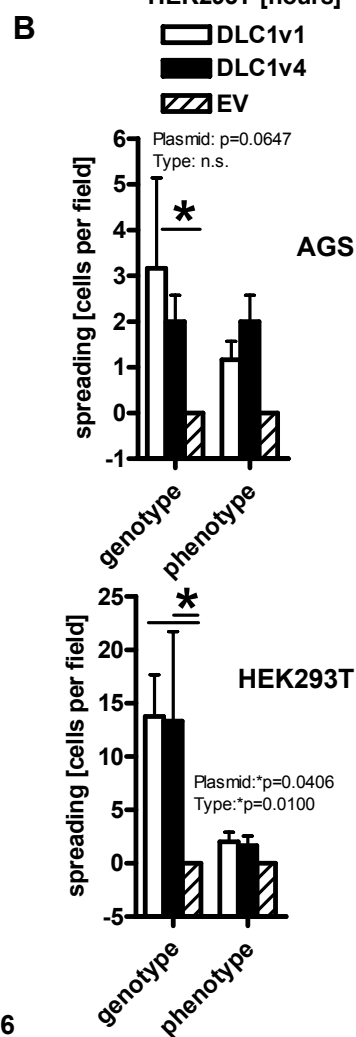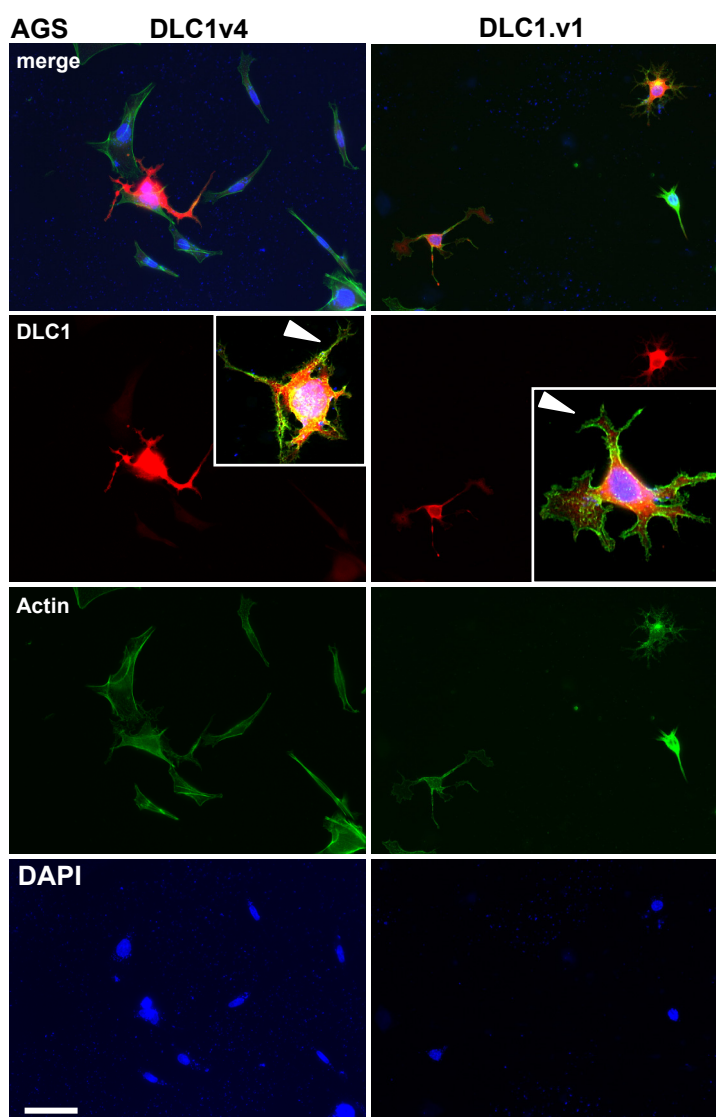

Supplement: Supplementary file 9 — Supplementary Figure S6 [file 41420_2022_1134_MOESM9_ESM.pdf]

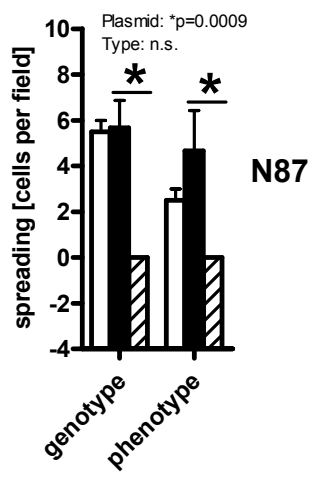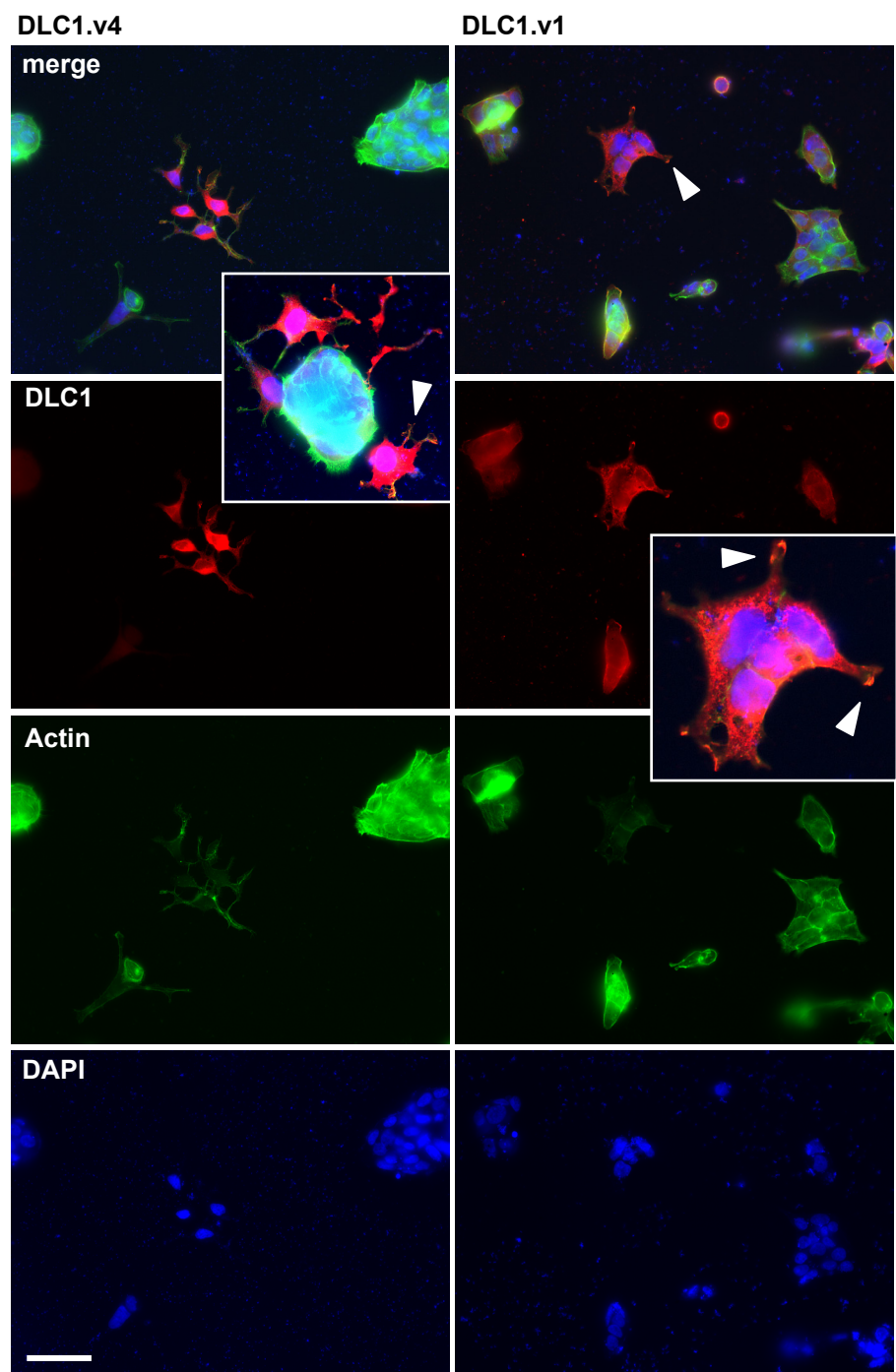

Supplement: Supplementary file 10 — Supplementary Figure S7 [file 41420_2022_1134_MOESM10_ESM.pdf]

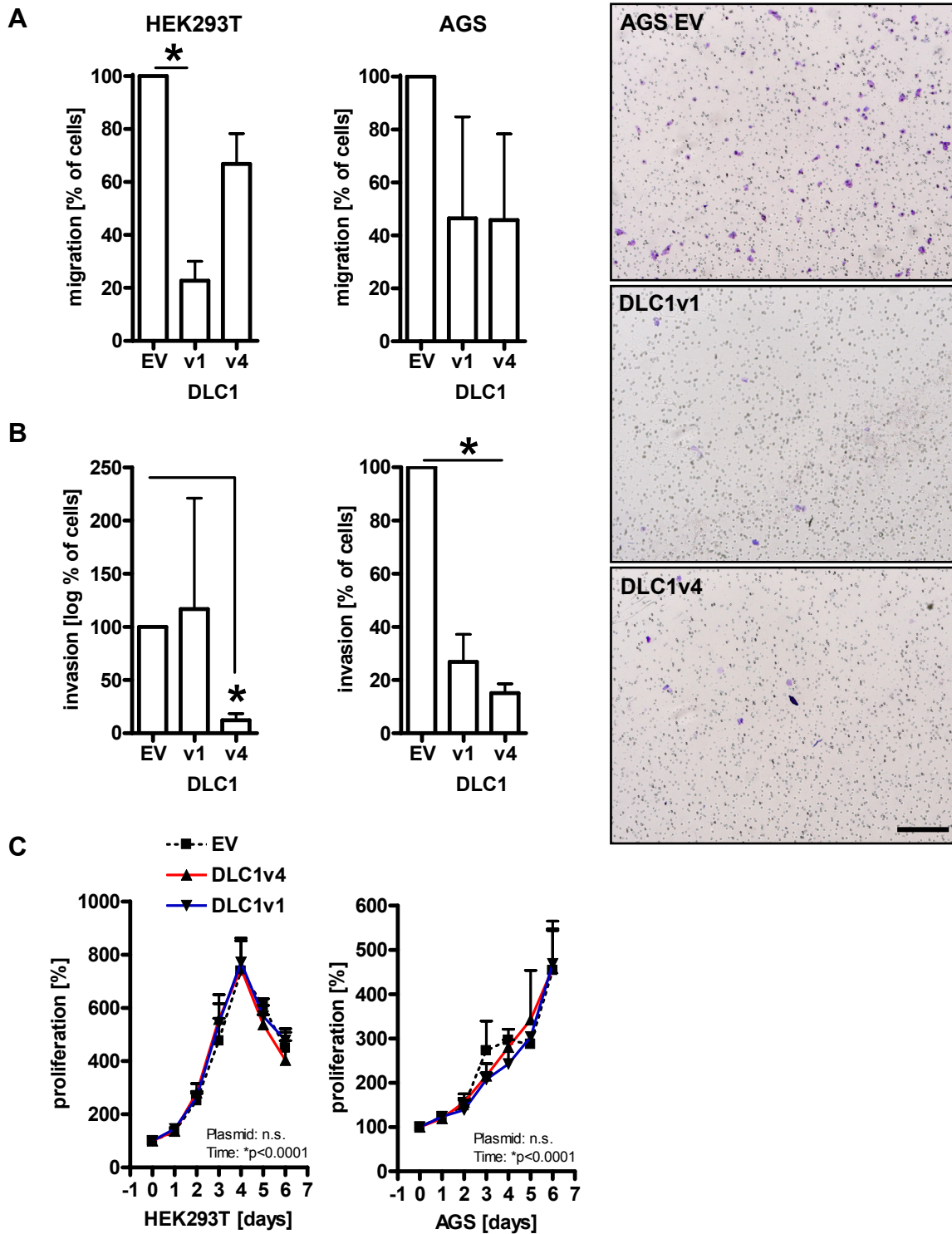

Supplement: Supplementary file 11 — Supplementary Figure S8 [file 41420_2022_1134_MOESM11_ESM.pdf]

**A**

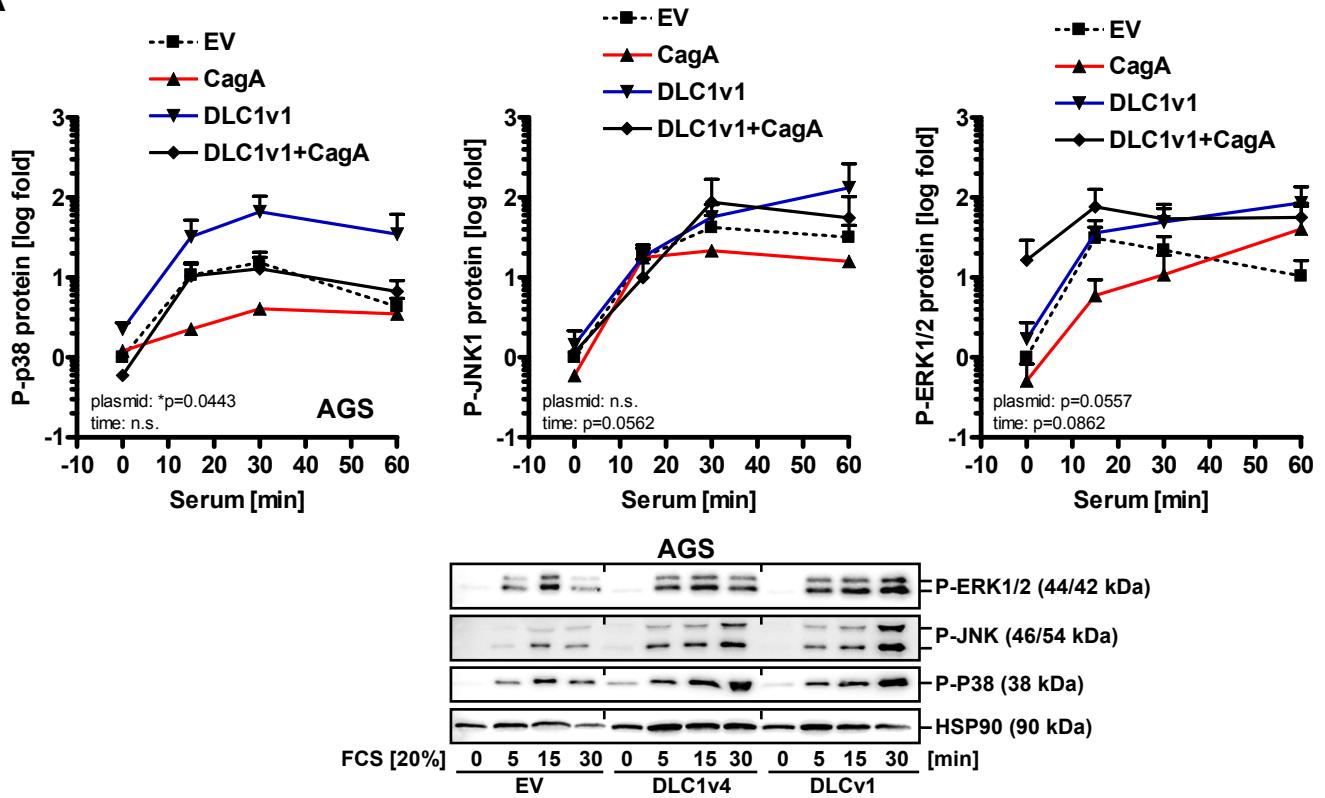

**B**

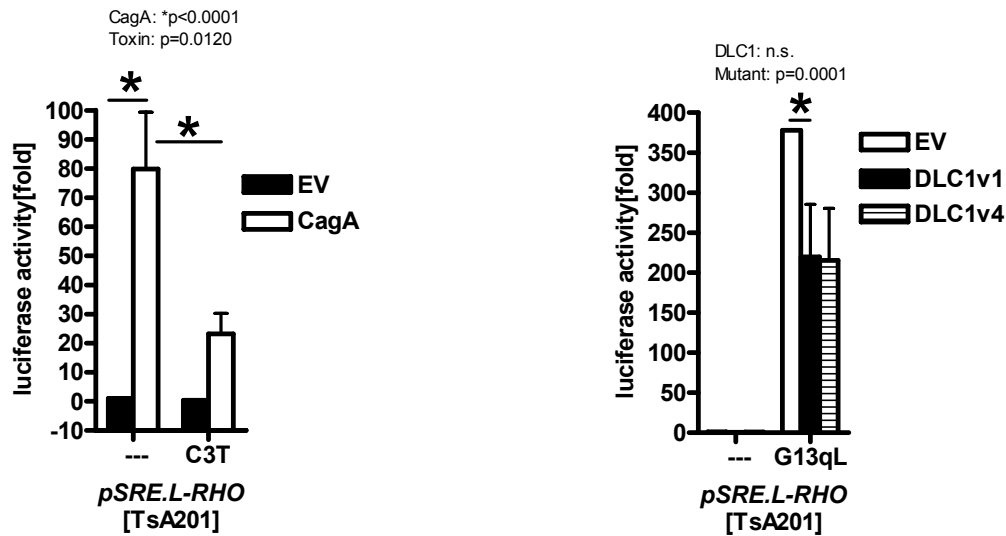

**C**

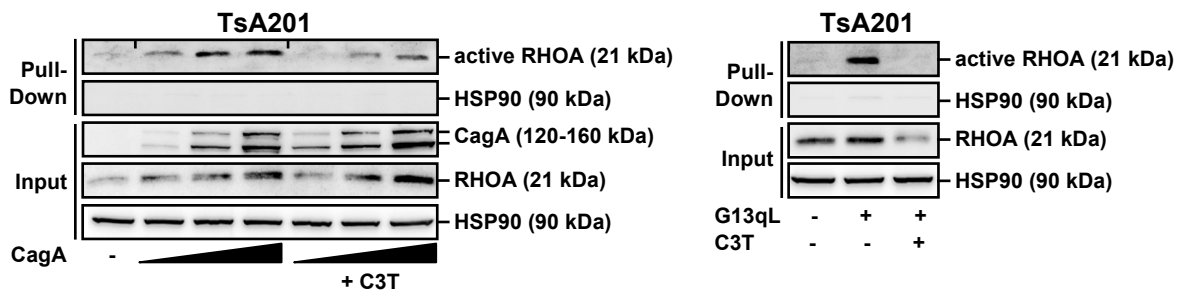

Supplement: Supplementary file 12 — Supplementary Figure S9 [file 41420_2022_1134_MOESM12_ESM.pdf]

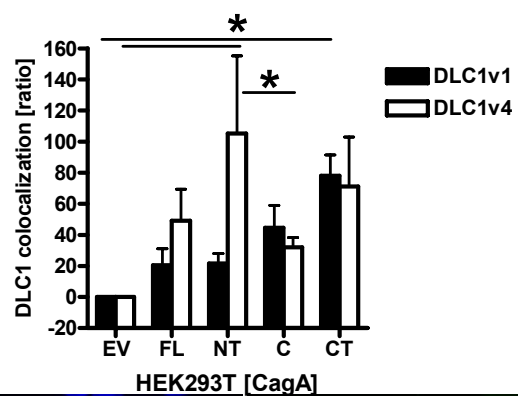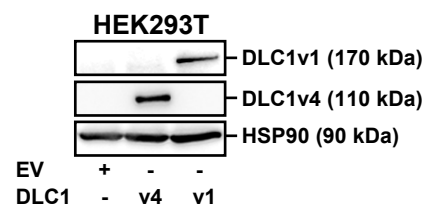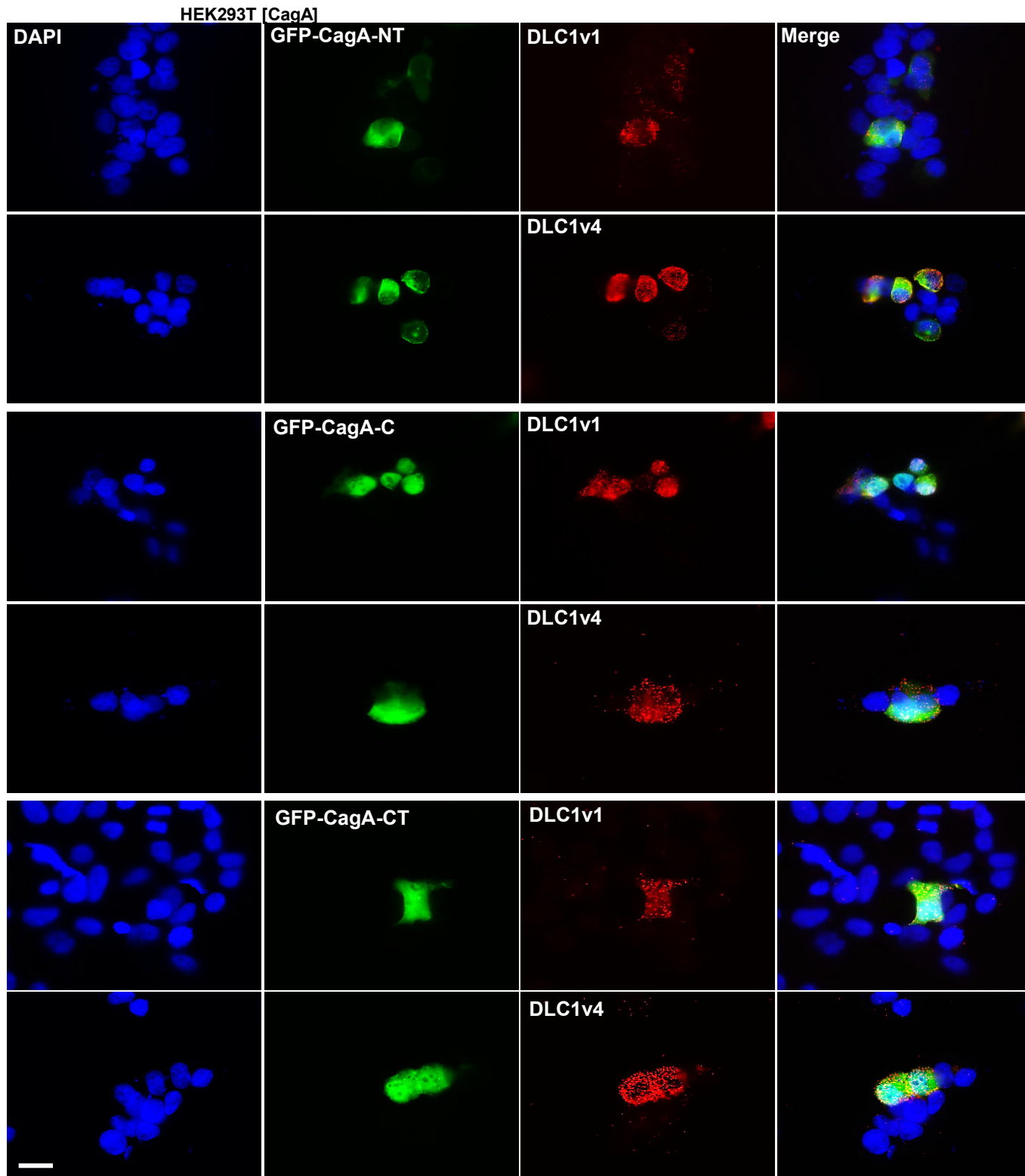

Supplement: Supplementary file 13 — Supplementary Figure S10 [file 41420_2022_1134_MOESM13_ESM.pdf]

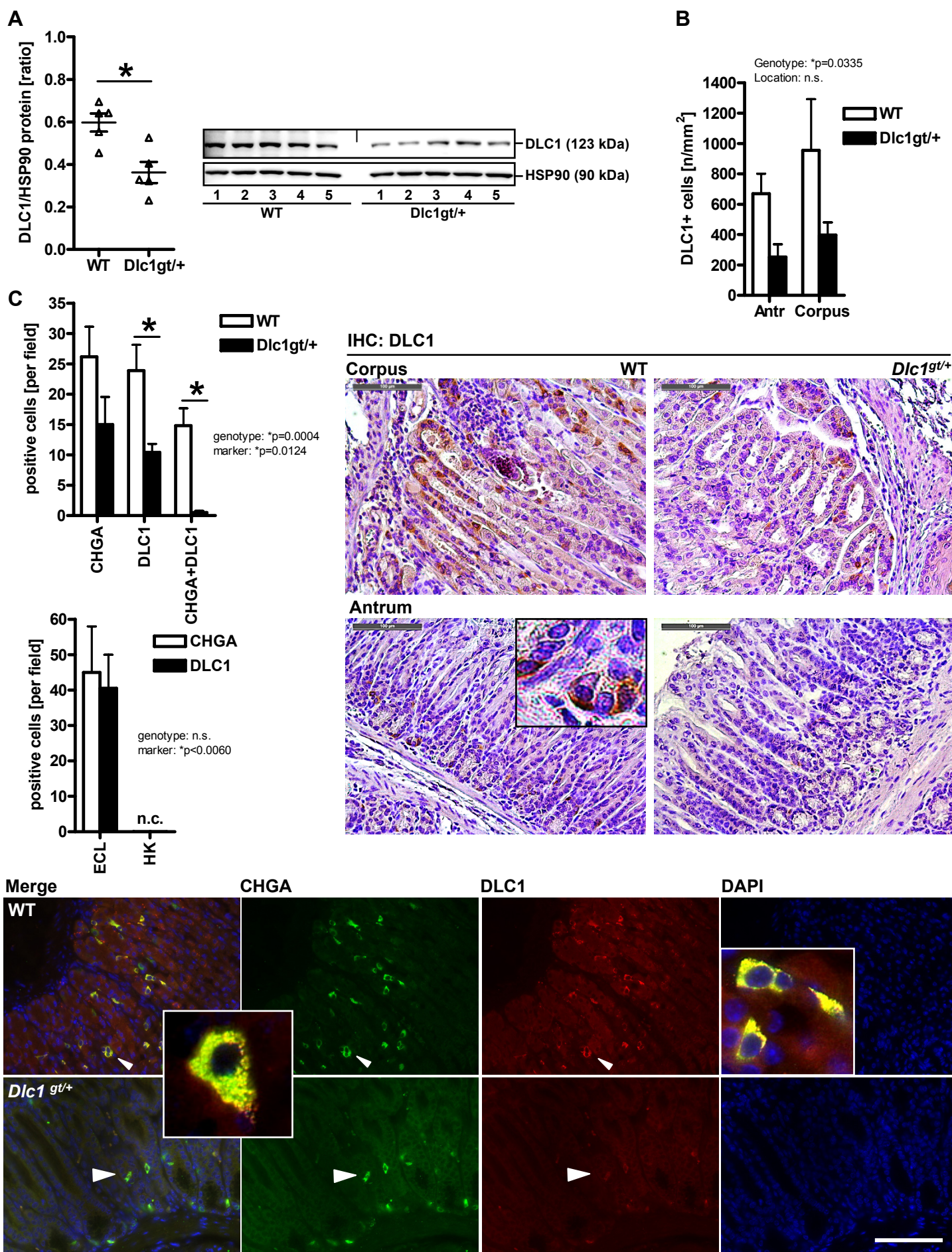

Supplement: Supplementary file 14 — Supplementary Figure S11 [file 41420_2022_1134_MOESM14_ESM.pdf]

# ECL cells

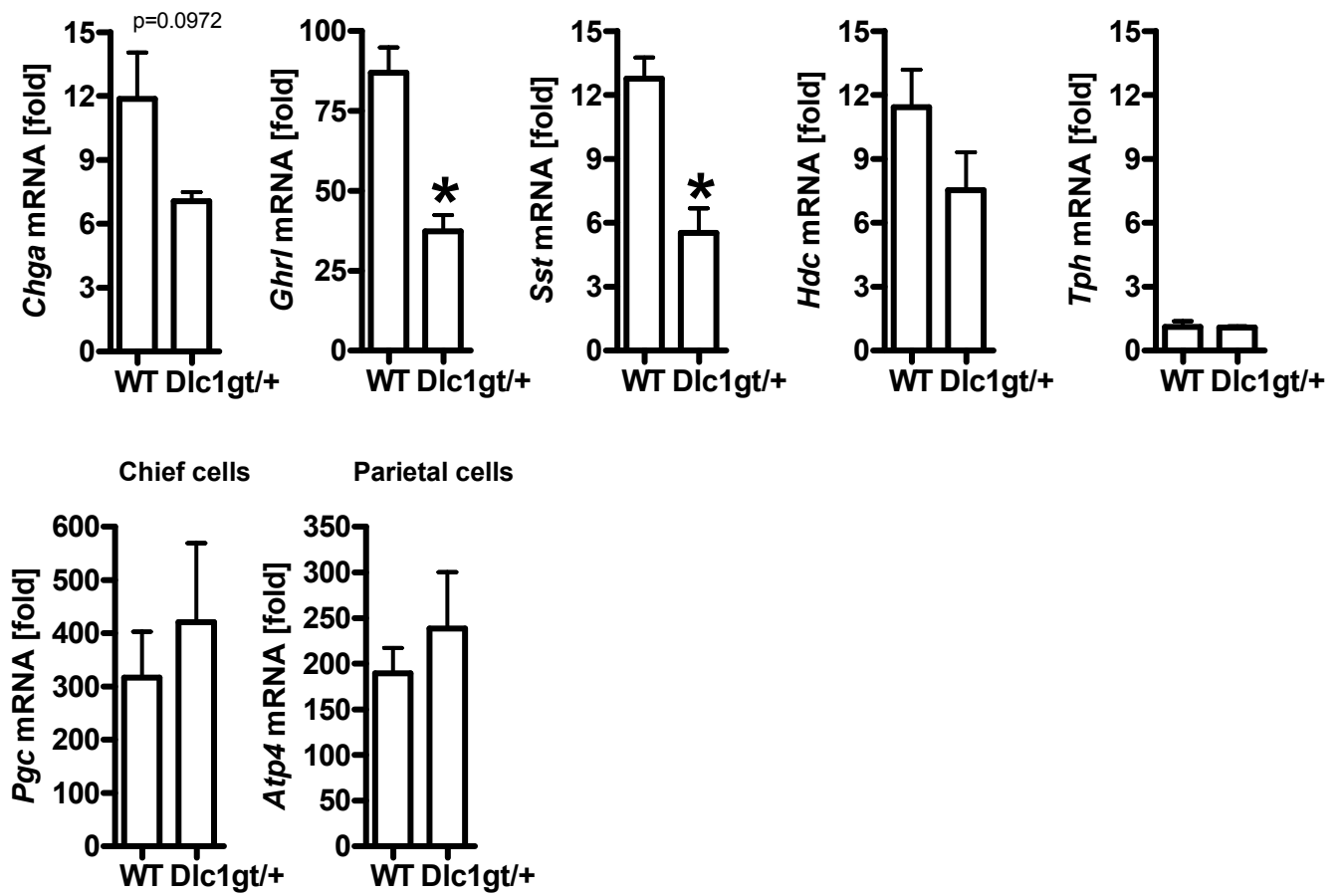

Supplement: Supplementary file 15 — Supplementary Figure S12 [file 41420_2022_1134_MOESM15_ESM.pdf]

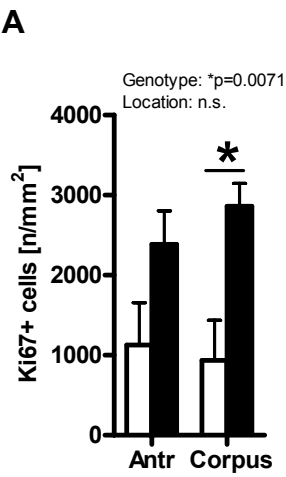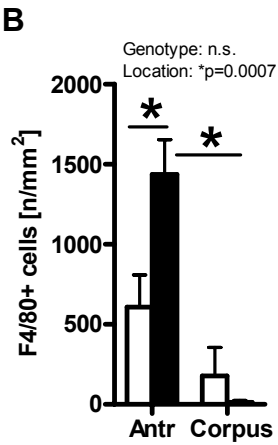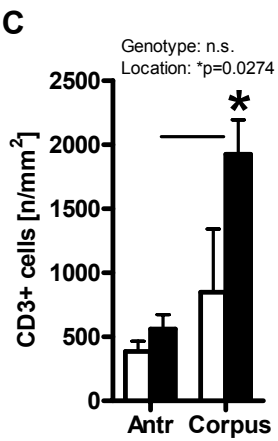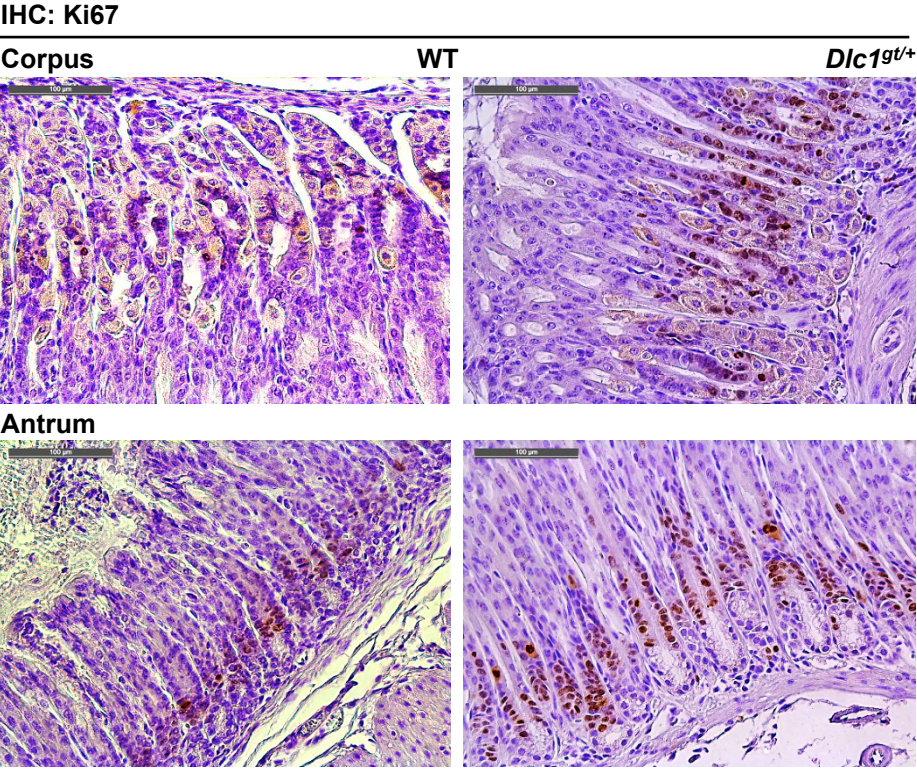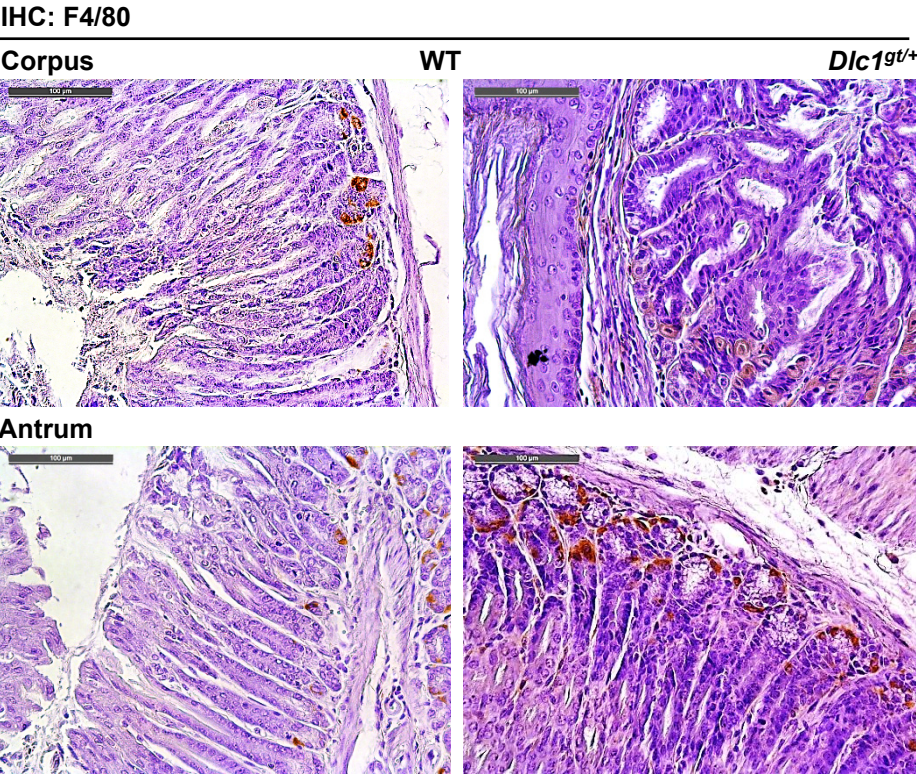

Supplement: Supplementary file 16 — Supplementary Figure S13 [file 41420_2022_1134_MOESM16_ESM.pdf]

A

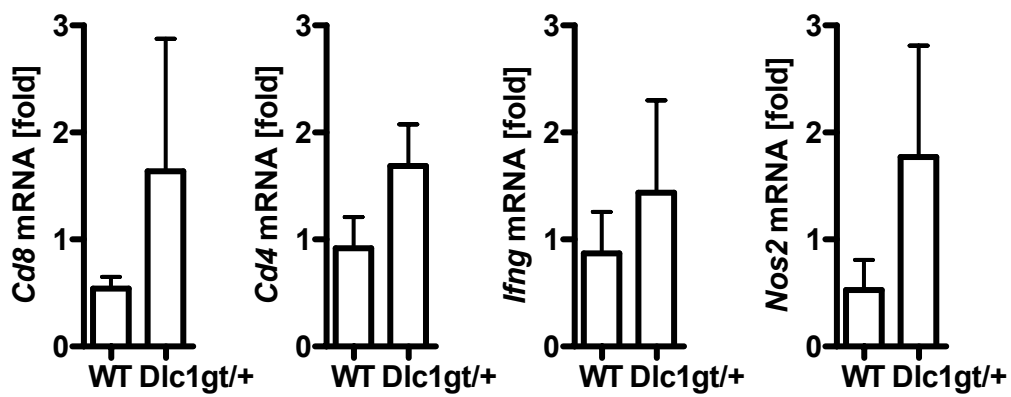

B

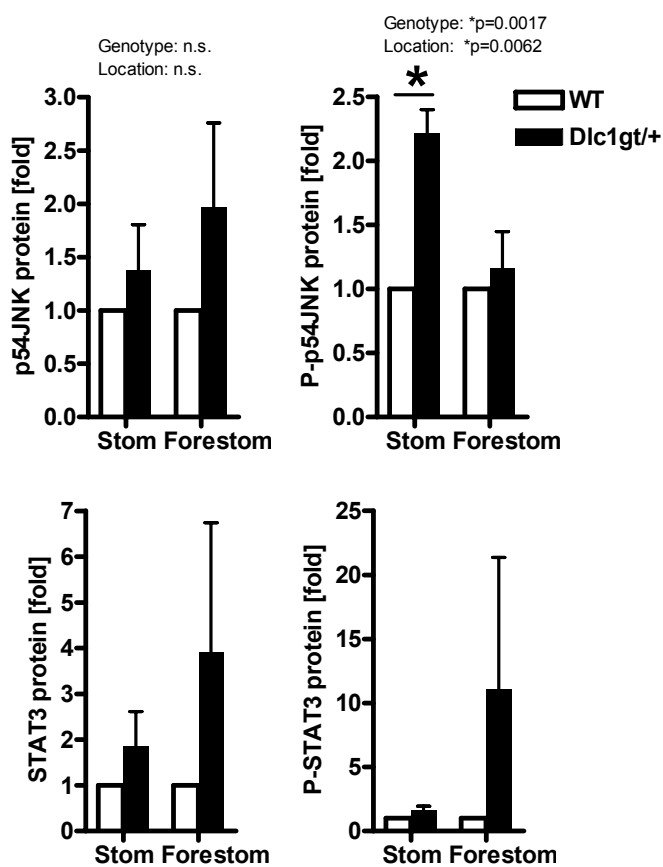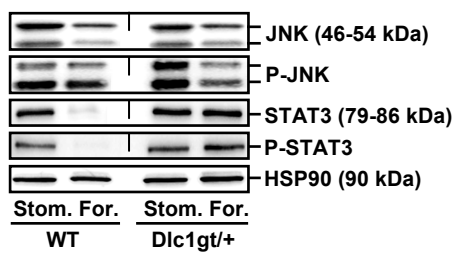

Supplement: Supplementary file 17 — Supplementary Figure S14 [file 41420_2022_1134_MOESM17_ESM.pdf]

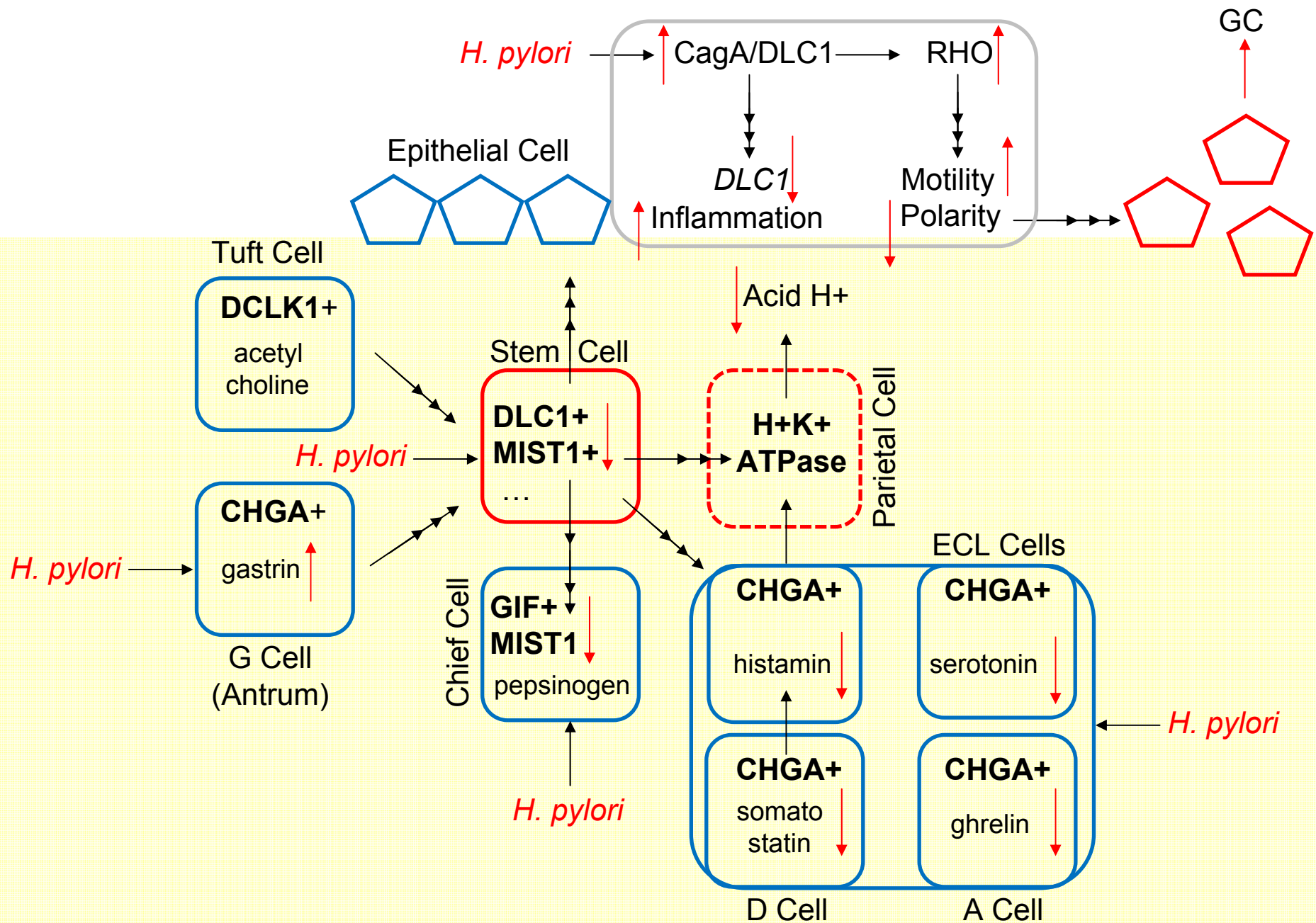

Supplement: Supplementary file 18 — Supplementary Figure S15 [file 41420_2022_1134_MOESM18_ESM.pdf]
