# Supplementary material for: Functional antagonism between CagA and DLC1 in gastric cancer
Source: Cell Death Discov. 2022 Aug 13;8:358. doi: 10.1038/s41420-022-01134-x (PMC9376073; doi:10.1038/s41420-022-01134-x)
Supplement: Supplementary file 19 — Original Western Blots [file 41420_2022_1134_MOESM19_ESM.pdf]

Original Western Blots

Main & supplement figures

Fig1E (left panel)

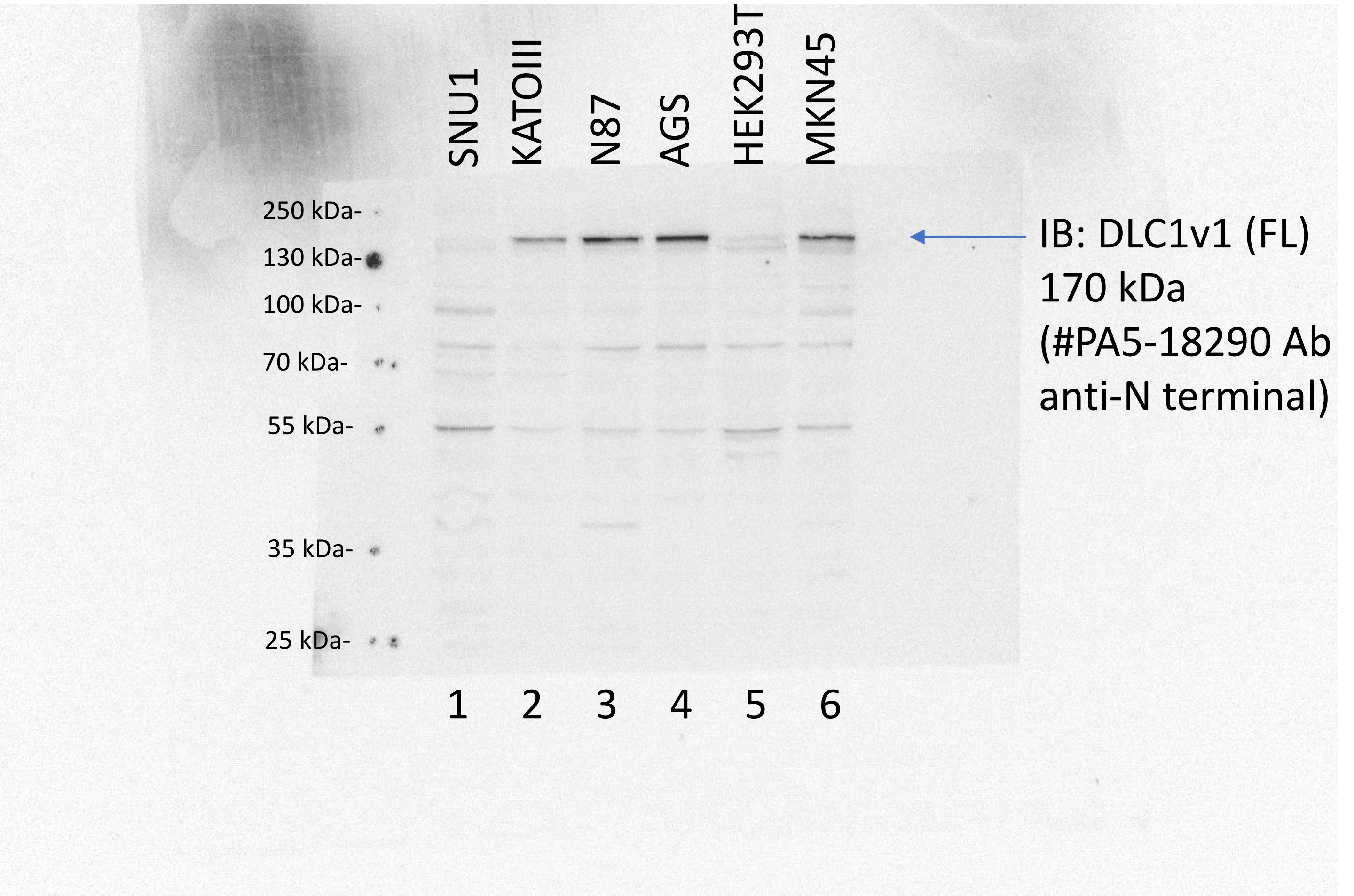

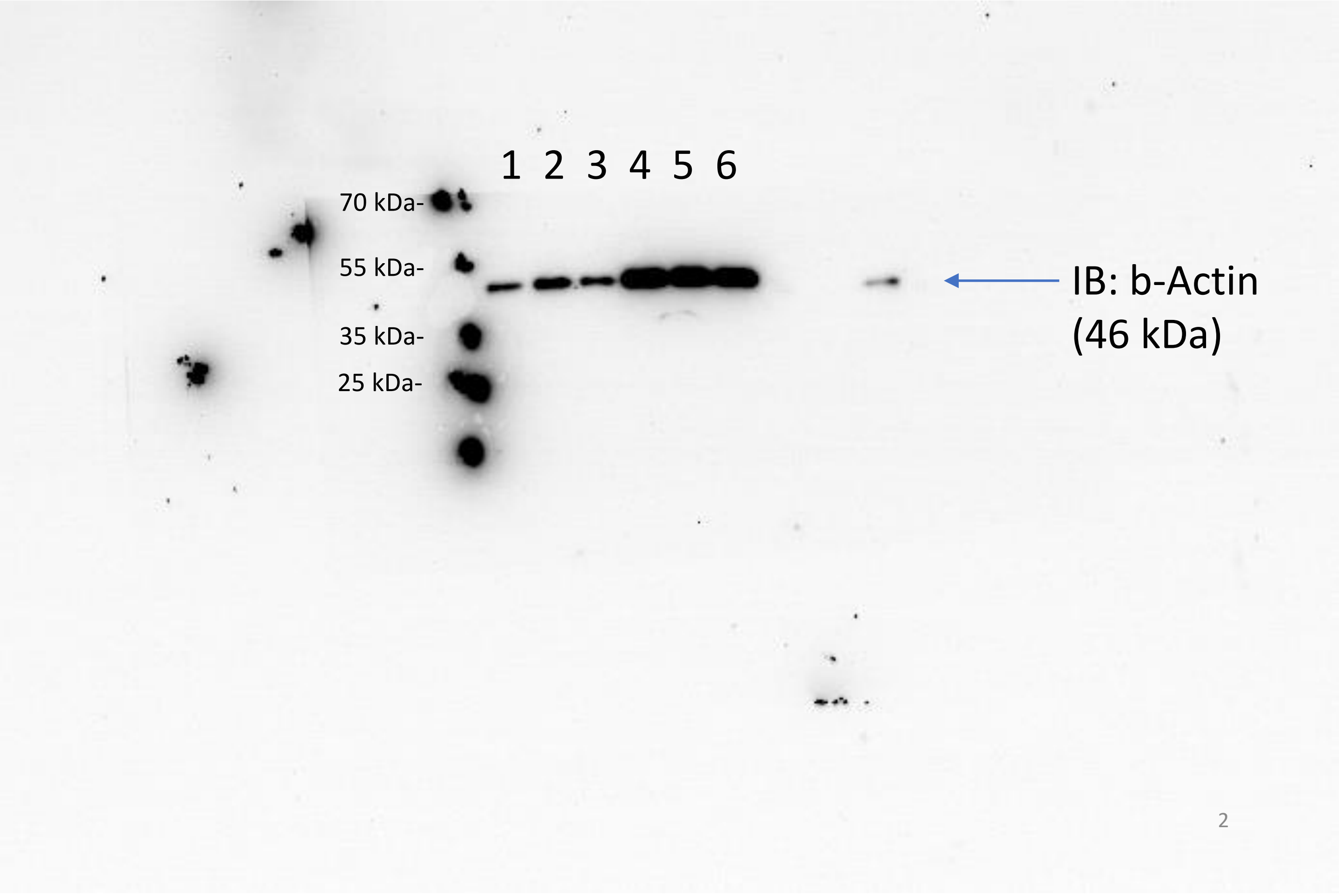

Fig1E (right panel)

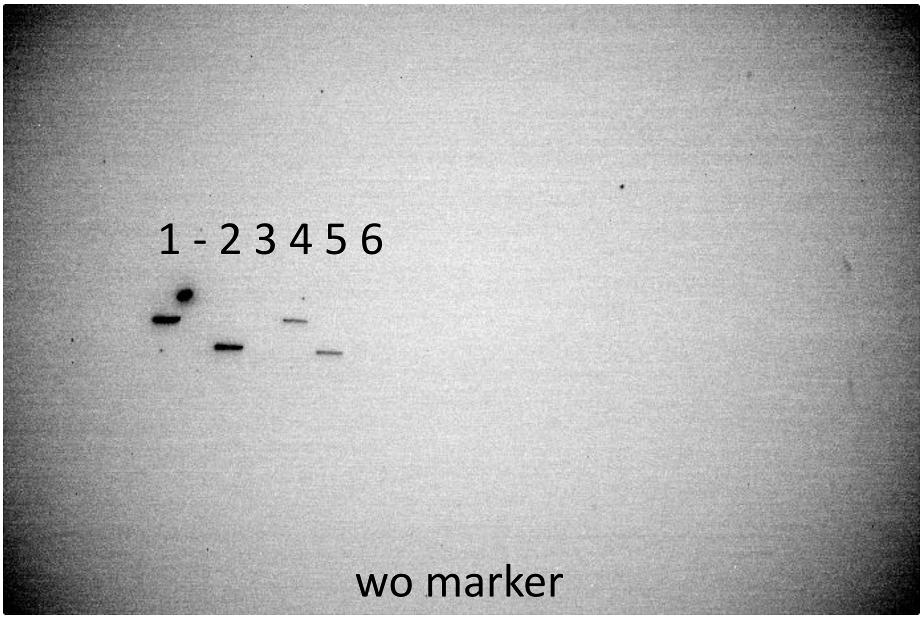

HEK293T:
1=DLC1v1 (FL)
- = empty lane
2=DLC1v4
3=EV
AGS:
4=DLC1v1
5=DLC1v4
6=EV

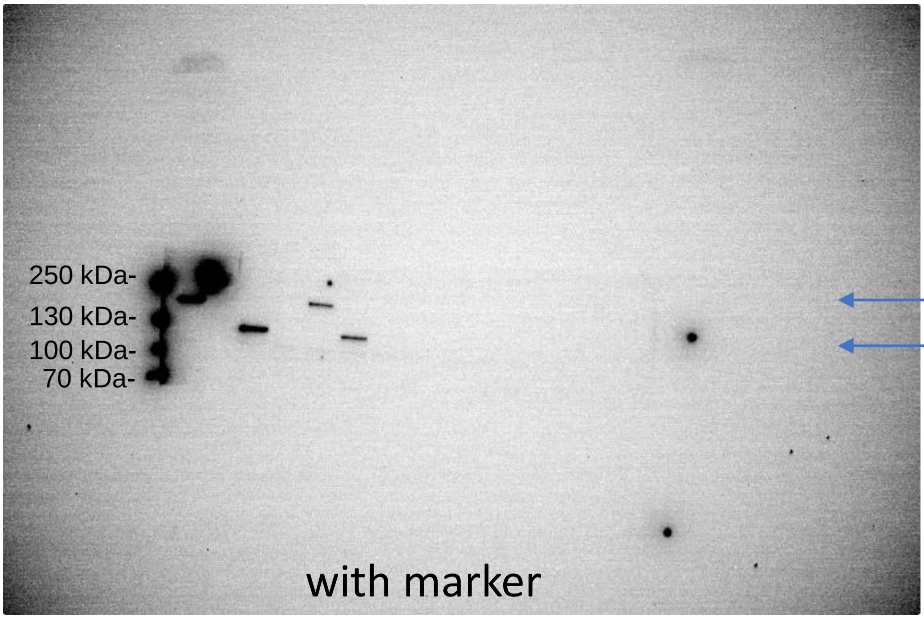

IB: anti-FLAG Ab
DLC1v1 (FL)
170 kDa
DLC1v4
110 kDa

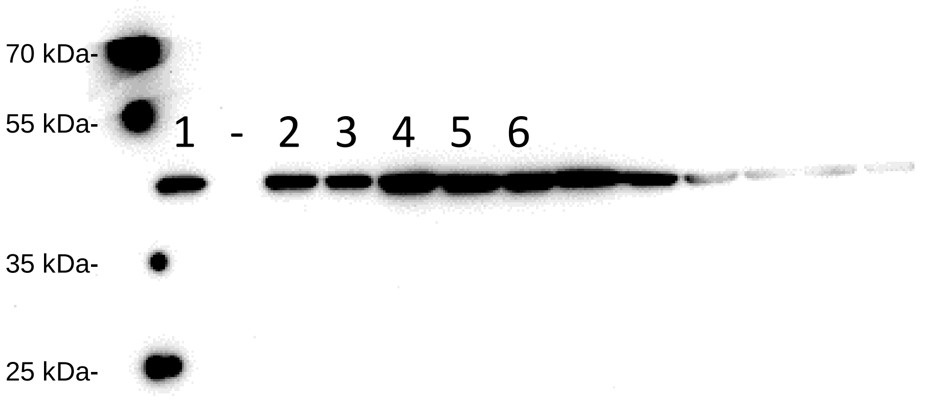

IB: b-Actin
(46 kDa)

Fig3A

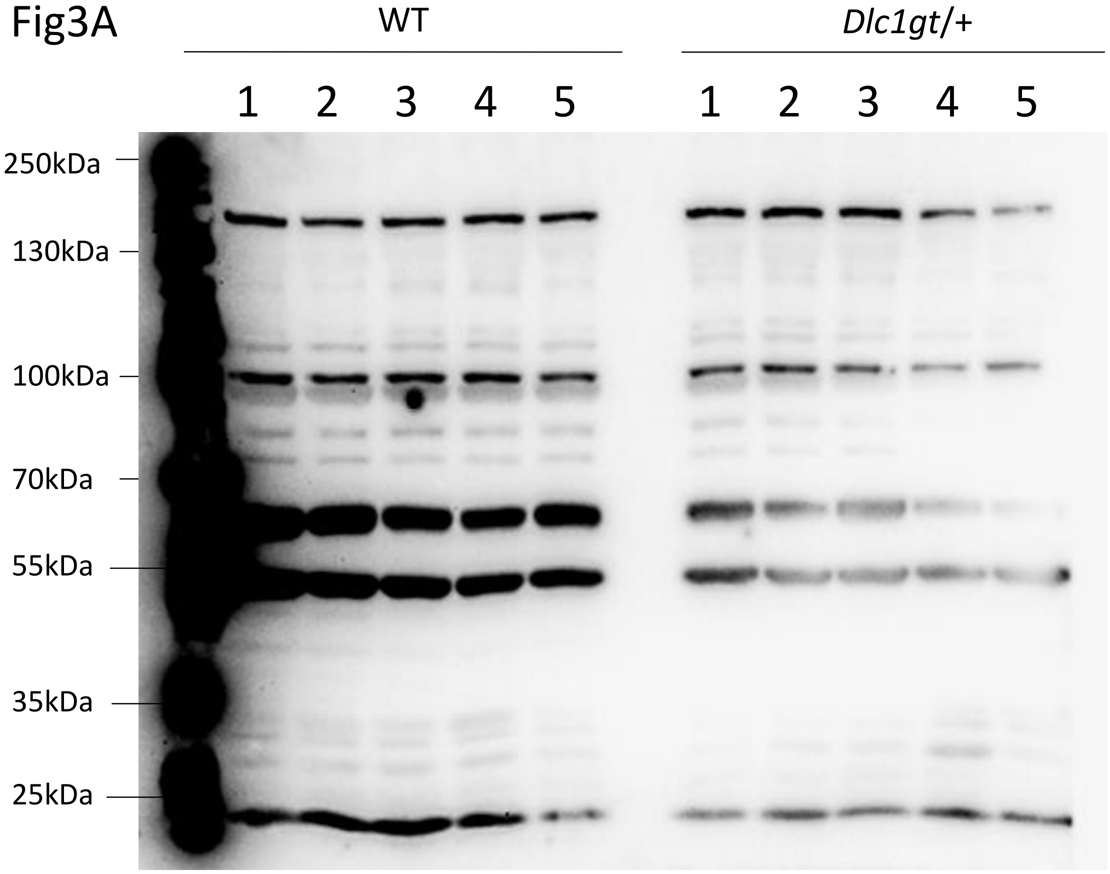

IB:
DLC1v1 mouse
170 kDa (FL)
(#H260/C12 Ab
anti-N terminal)

Liver tissue

n=5 mice
per genotype

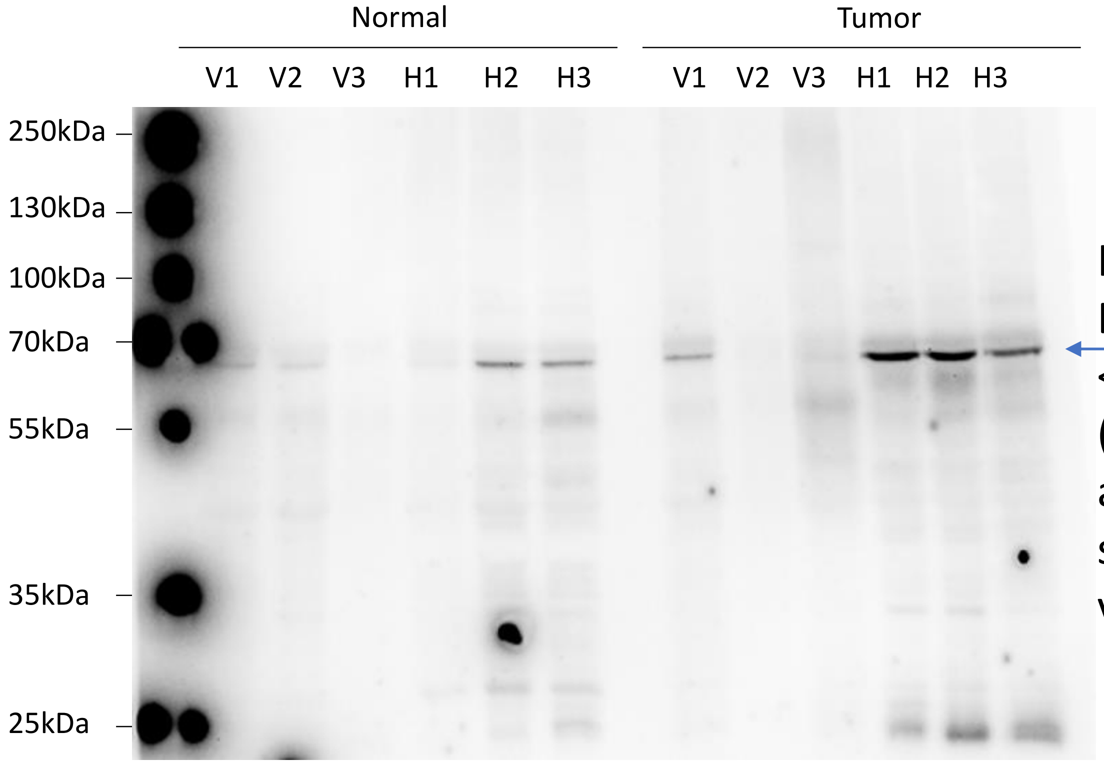

IB:
DLC1 mouse
<70 kDa
(#H260/C12 Ab
anti-N terminal)
small cancer
variant

Stomach tissue

n=3 mice per
genotype

H = Corpus (stomach)
V = Forestomach

Fig5D (top)

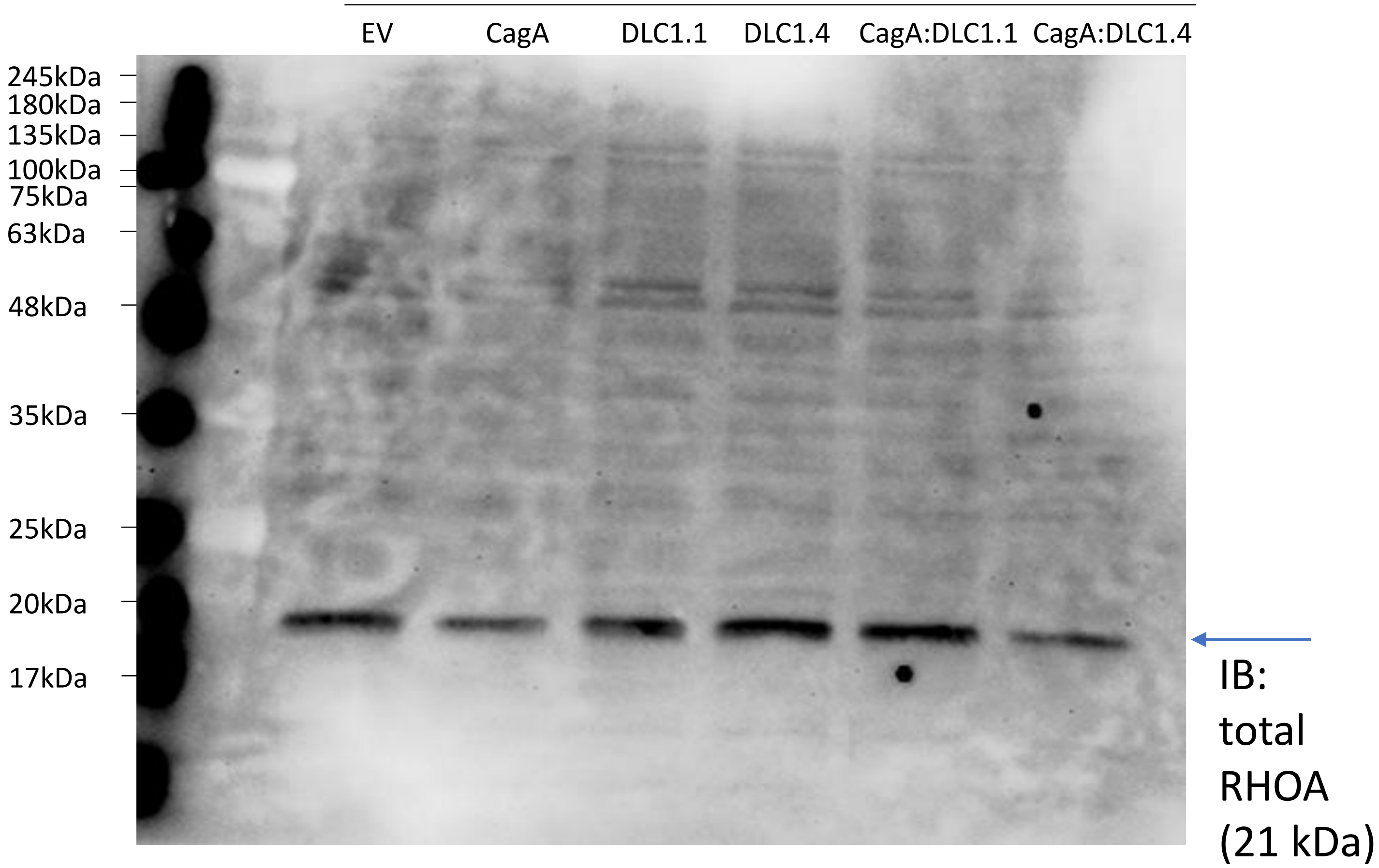
Input
EV
CagA
DLC1.1
DLC1.4
CagA:DLC1.1
CagA:DLC1.4
245kDa
180kDa
135kDa
100kDa
75kDa
63kDa
48kDa
35kDa
25kDa
20kDa
17kDa
IB: total RHOA (21 kDa)

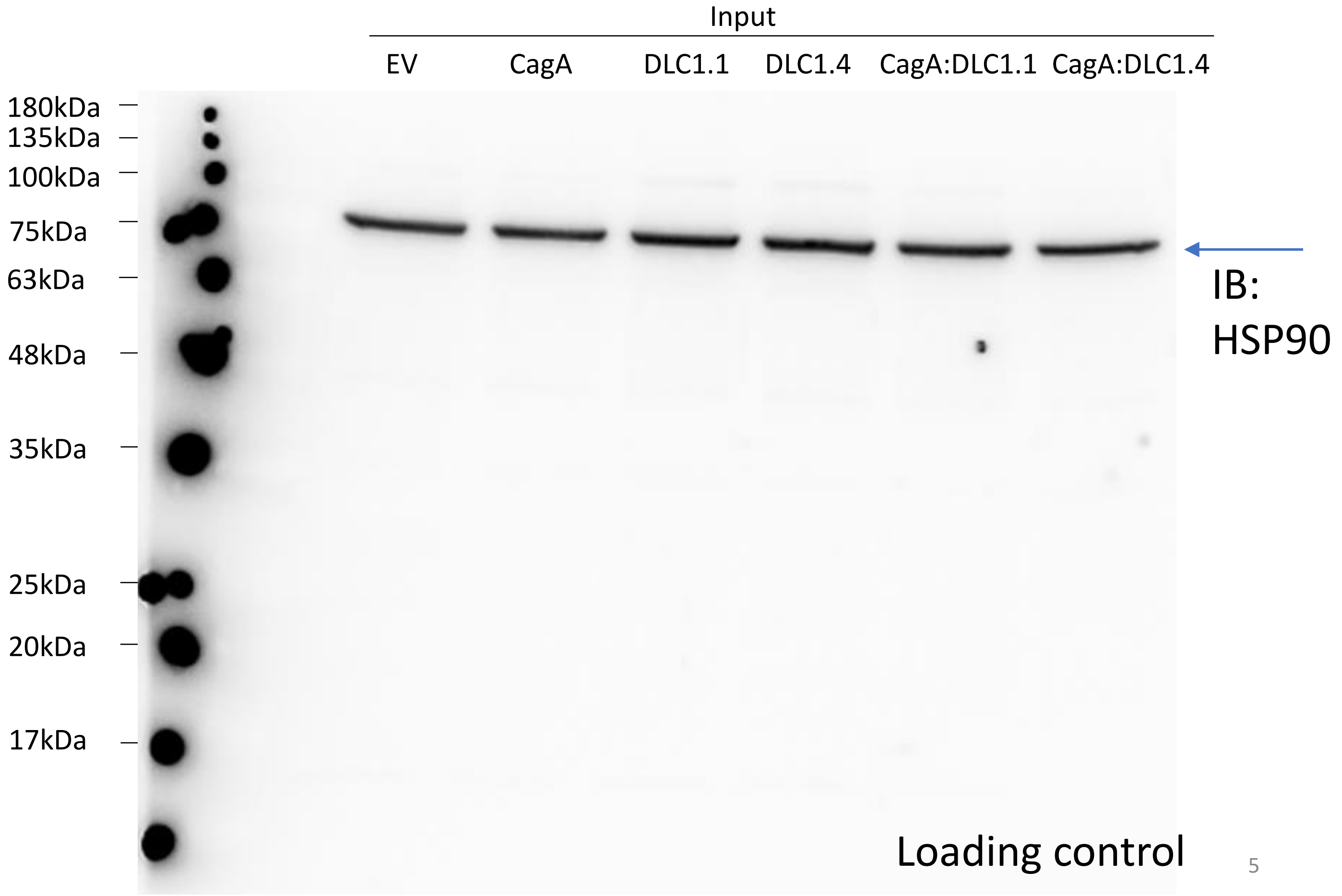
Input
EV
CagA
DLC1.1
DLC1.4
CagA:DLC1.1
CagA:DLC1.4
180kDa
135kDa
100kDa
75kDa
63kDa
48kDa
35kDa
25kDa
20kDa
17kDa
IB: HSP90
Loading control

Fig5D (top)

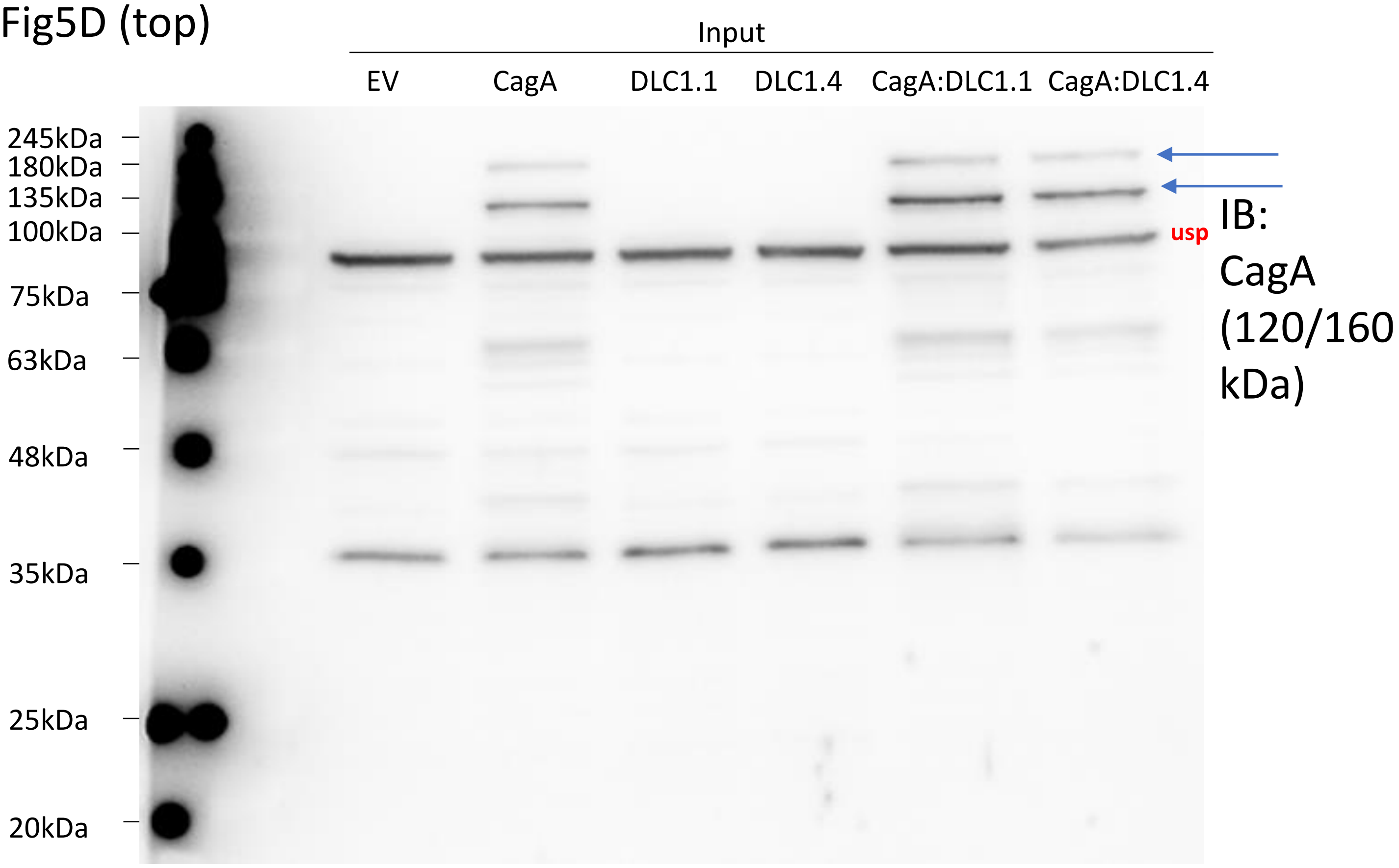

same membrane
re-incubated with HSP90 Ab

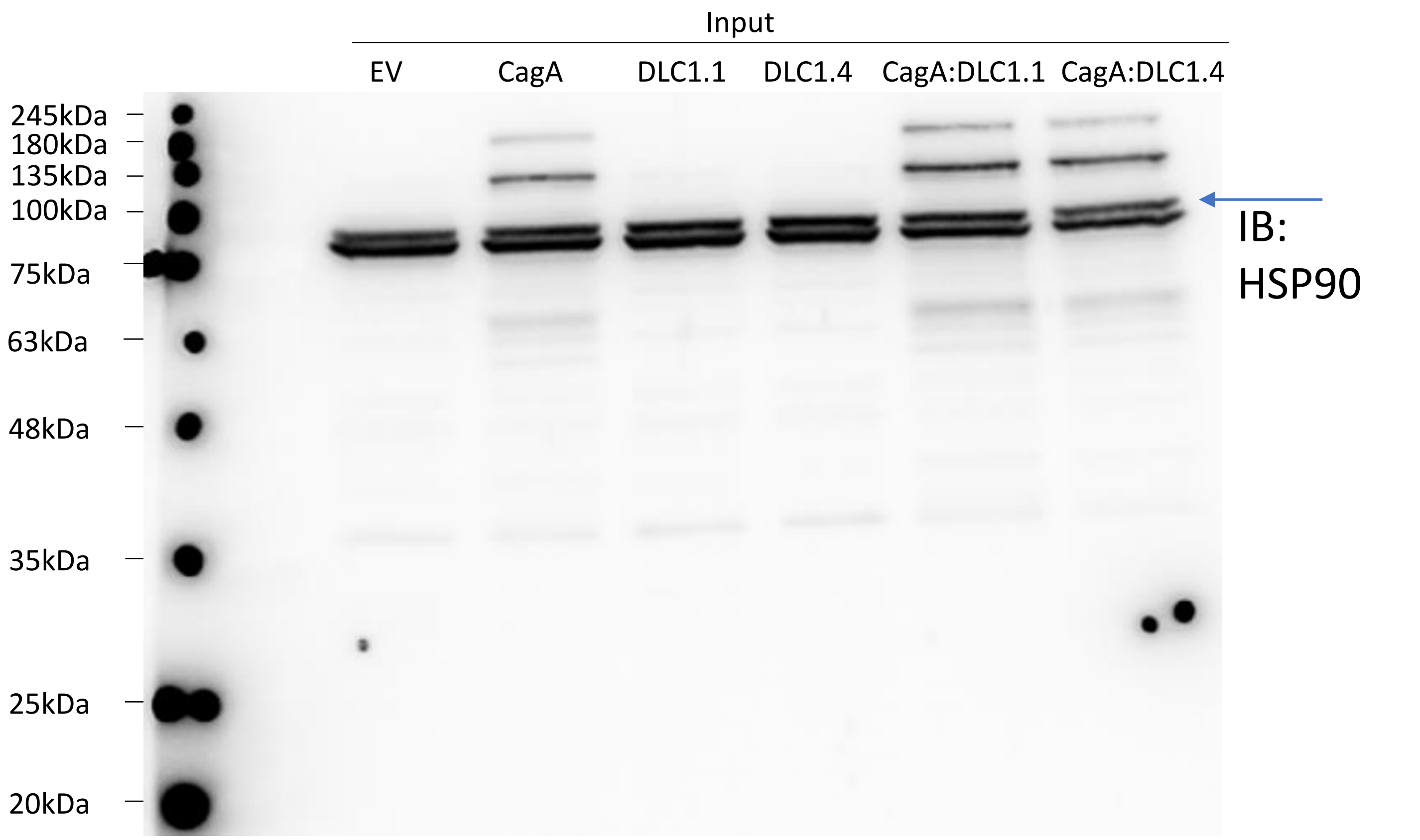

Loading control

Fig5D (top)

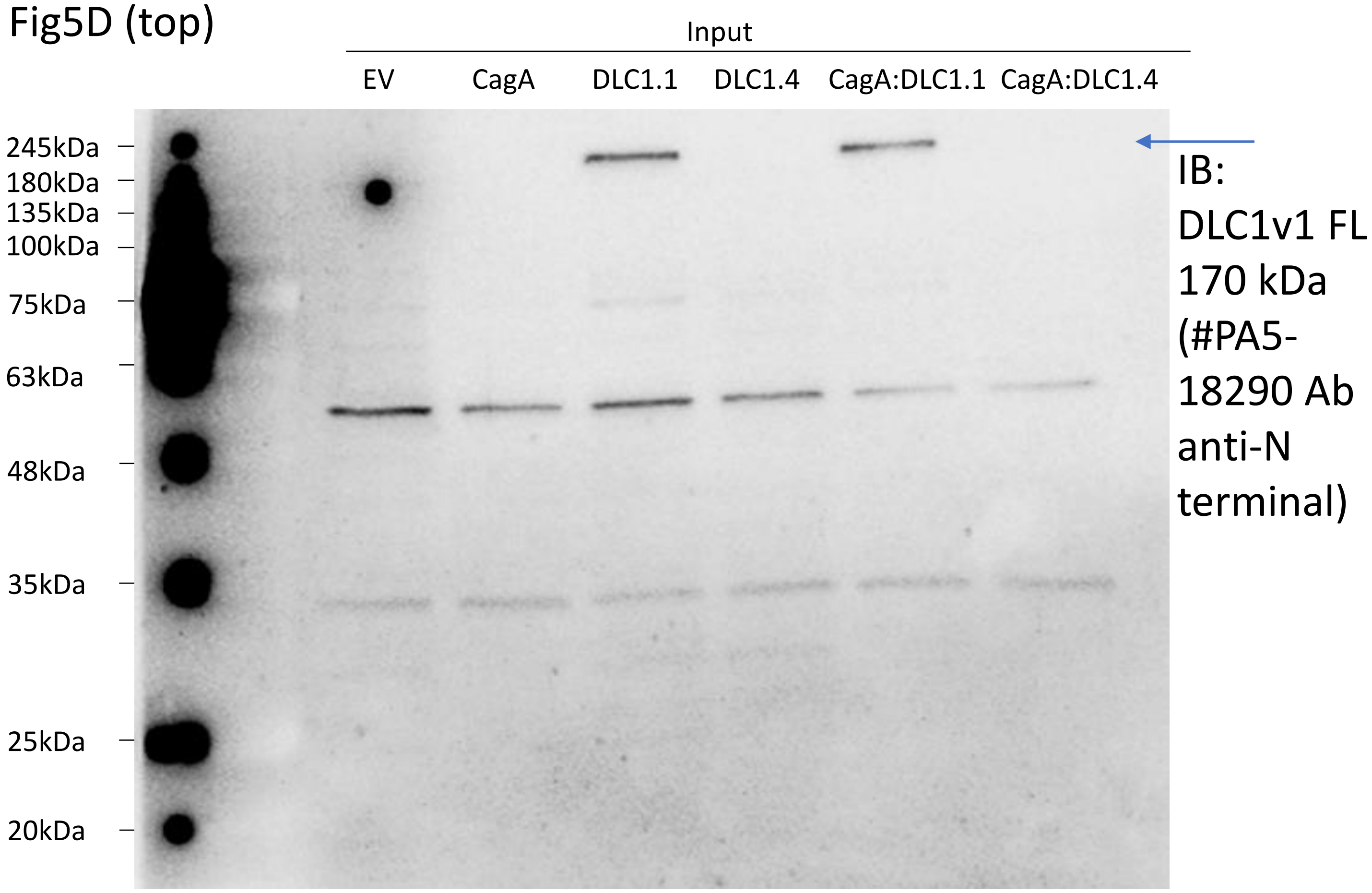
Input
EV
CagA
DLC1.1
DLC1.4
CagA:DLC1.1
CagA:DLC1.4
245kDa
180kDa
135kDa
100kDa
75kDa
63kDa
48kDa
35kDa
25kDa
20kDa
IB: DLC1v1 FL 170 kDa (#PA5-18290 Ab anti-N terminal)

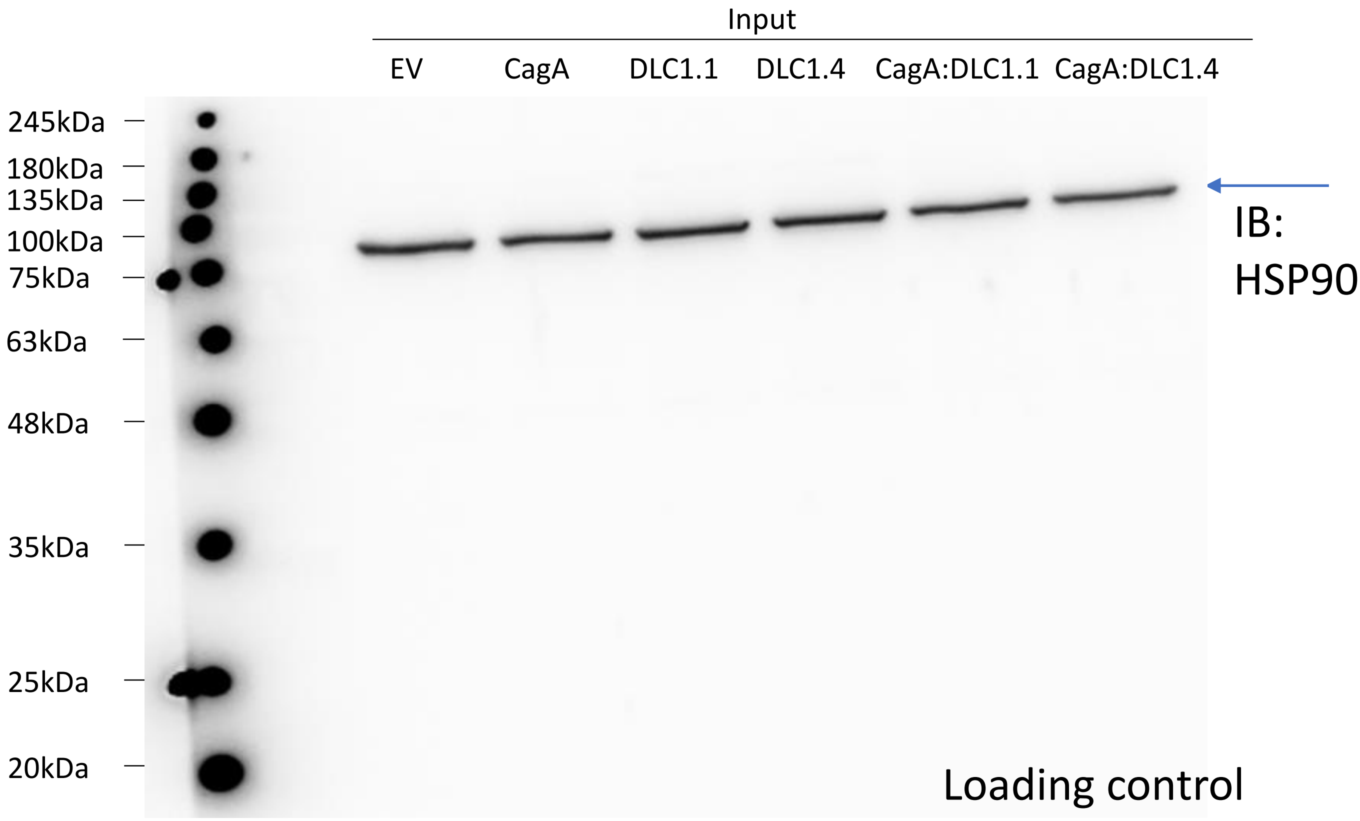
Input
EV
CagA
DLC1.1
DLC1.4
CagA:DLC1.1
CagA:DLC1.4
245kDa
180kDa
135kDa
100kDa
75kDa
63kDa
48kDa
35kDa
25kDa
20kDa
IB: HSP90
Loading control

Fig5D (top)

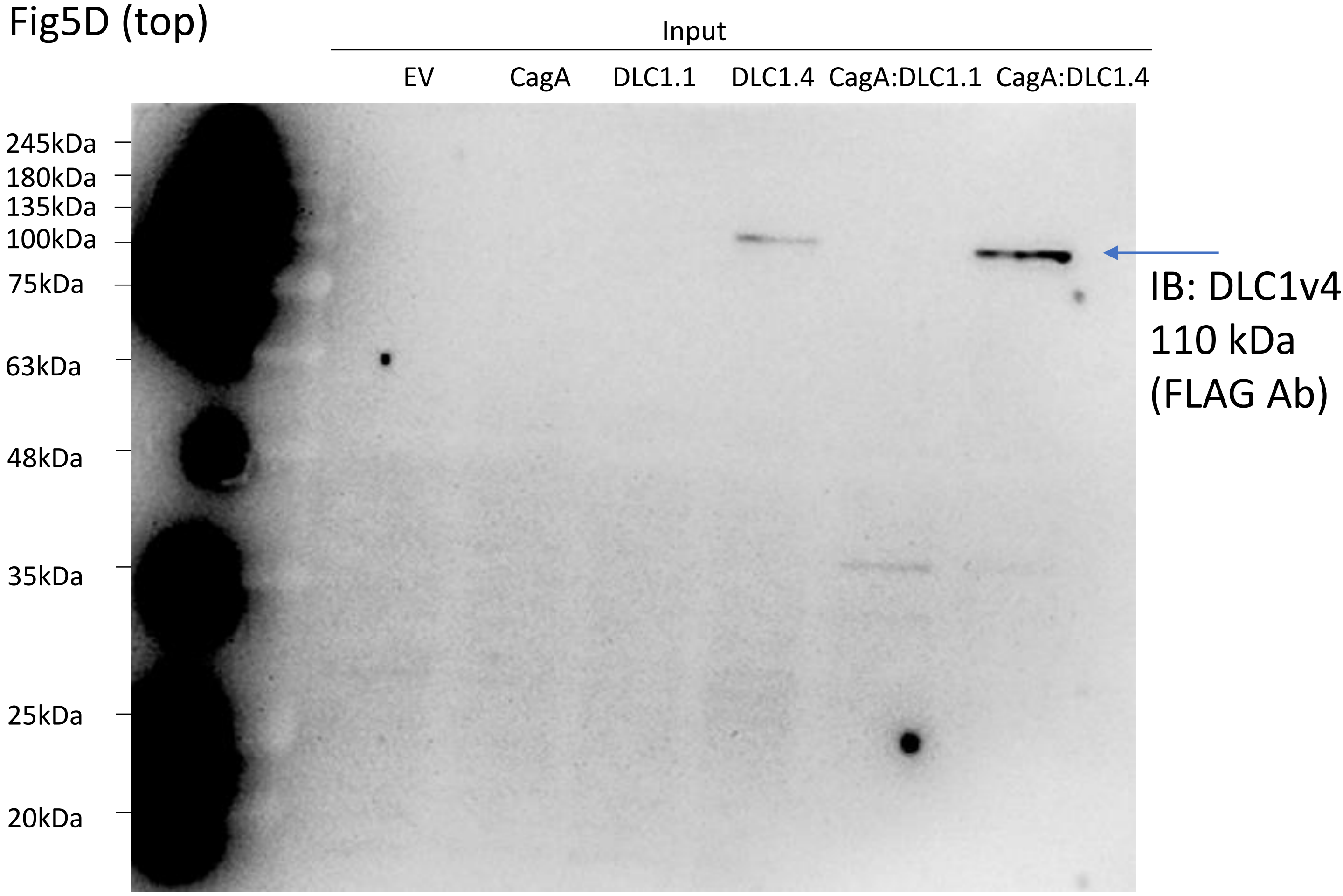
Input
EV
CagA
DLC1.1
DLC1.4
CagA:DLC1.1
CagA:DLC1.4
245kDa
180kDa
135kDa
100kDa
75kDa
63kDa
48kDa
35kDa
25kDa
20kDa
IB: DLC1v4
110 kDa
(FLAG Ab)

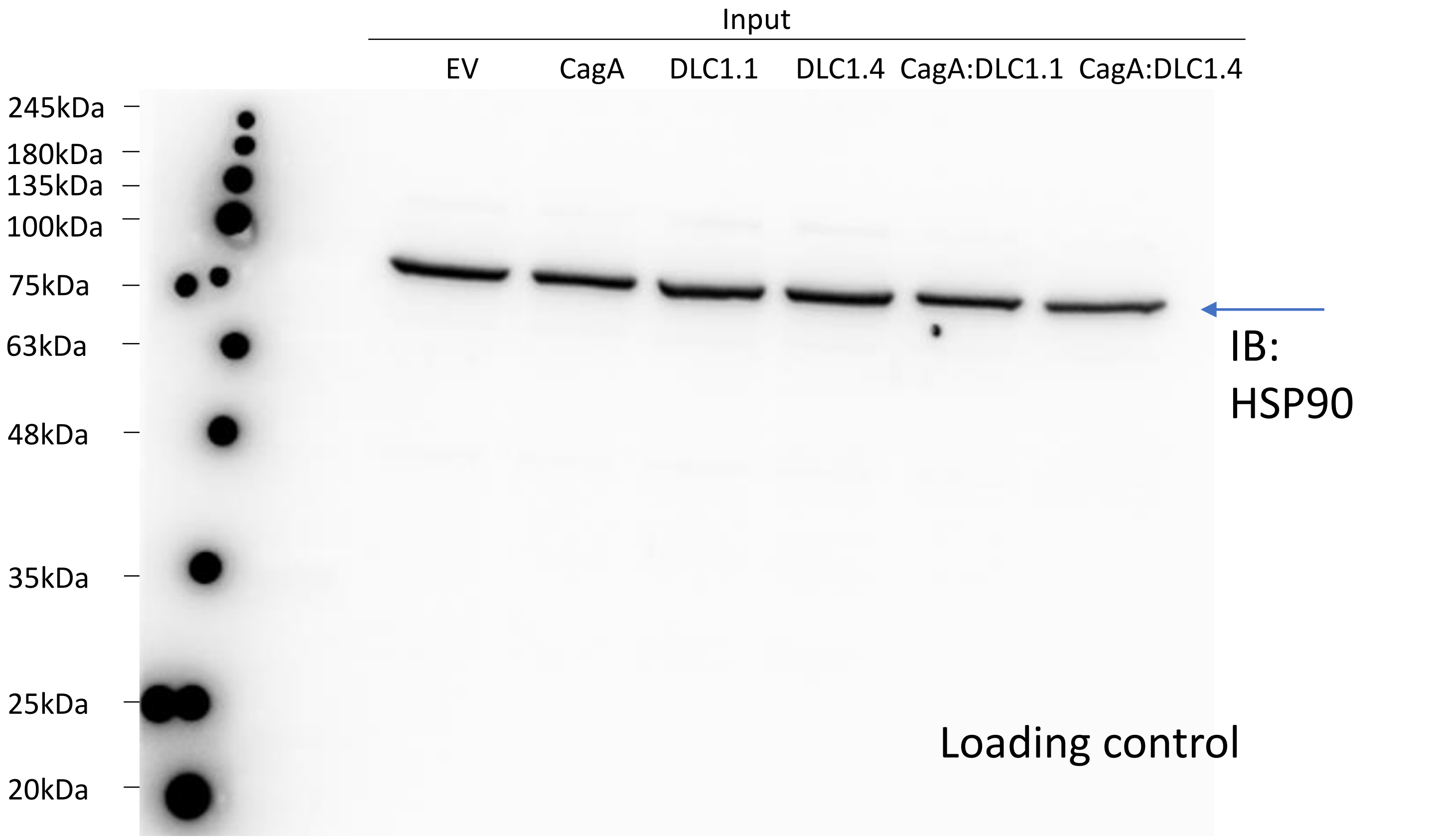
Input
EV
CagA
DLC1.1
DLC1.4
CagA:DLC1.1
CagA:DLC1.4
245kDa
180kDa
135kDa
100kDa
75kDa
63kDa
48kDa
35kDa
25kDa
20kDa
IB:
HSP90
Loading control

Fig5D (top)

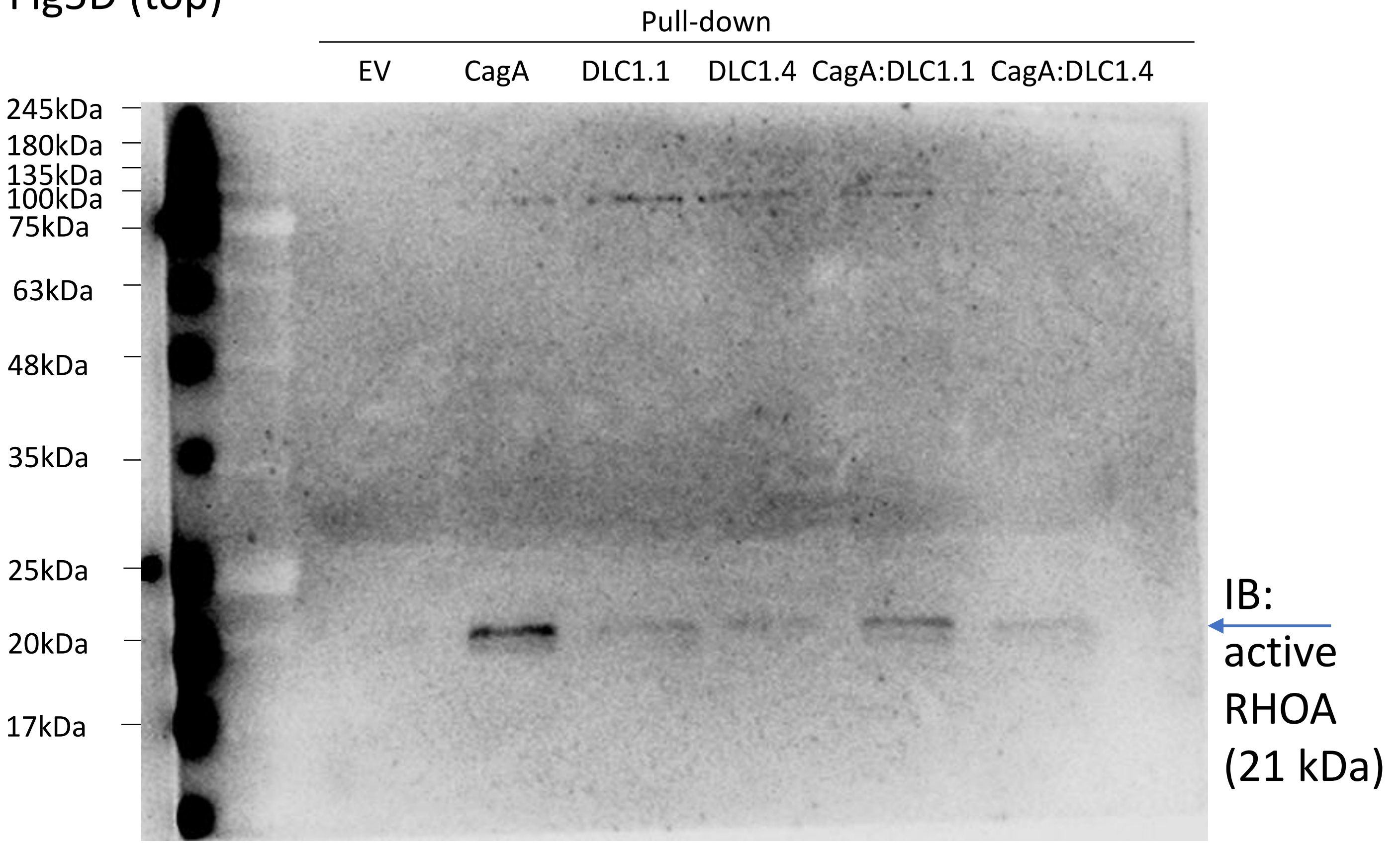
Pull-down
EV
CagA
DLC1.1
DLC1.4
CagA:DLC1.1
CagA:DLC1.4
245kDa
180kDa
135kDa
100kDa
75kDa
63kDa
48kDa
35kDa
25kDa
20kDa
17kDa
IB: active RHOA (21 kDa)

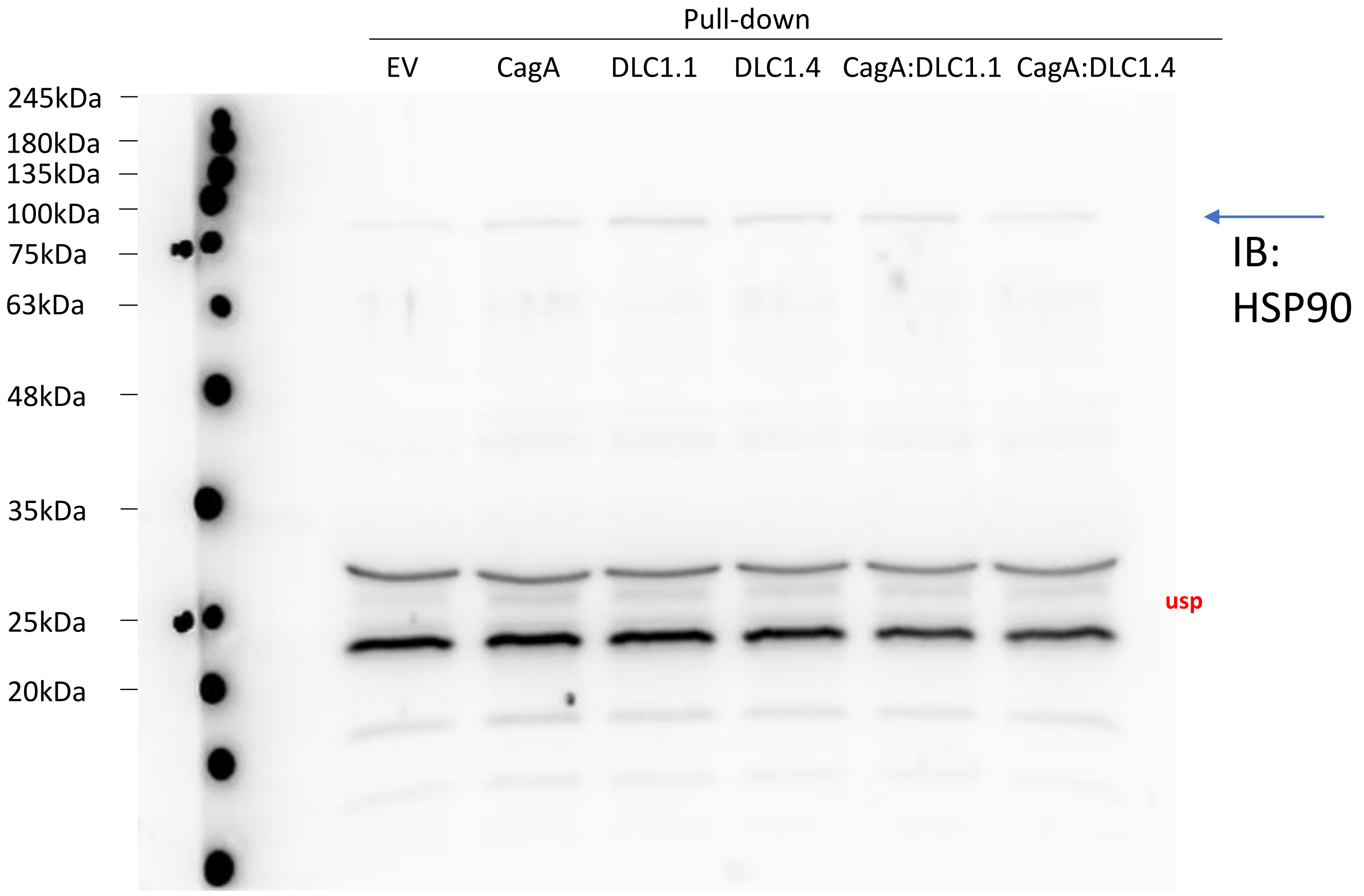
Pull-down
EV
CagA
DLC1.1
DLC1.4
CagA:DLC1.1
CagA:DLC1.4
245kDa
180kDa
135kDa
100kDa
75kDa
63kDa
48kDa
35kDa
25kDa
20kDa
IB: HSP90
usp
Purity control

Fig5D (bottom)

Pulldown AGS | Pulldown TsA

EV CagA DLC1.1 DLC1.4 CagA +v1 CagA +v4 EV CagA

245kDa
180kDa
135kDa
100kDa
75kDa
63kDa
48kDa
35kDa
25kDa
20kDa
17kDa

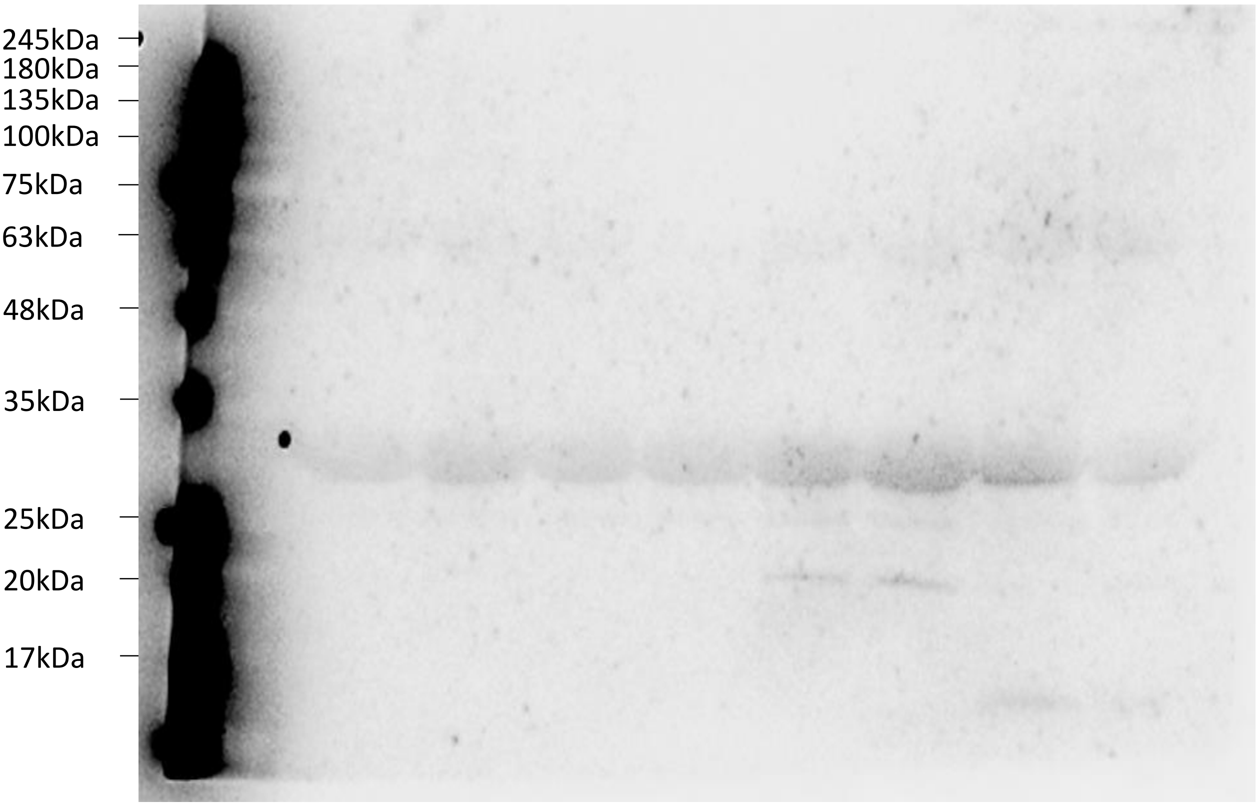

IB: active RHOA (21 kDa)

Short exposure

Pulldown AGS | Pulldown TsA

--- EV CagA DLC1.1 DLC1.4 CagA +v1 CagA +v4 EV CagA ---

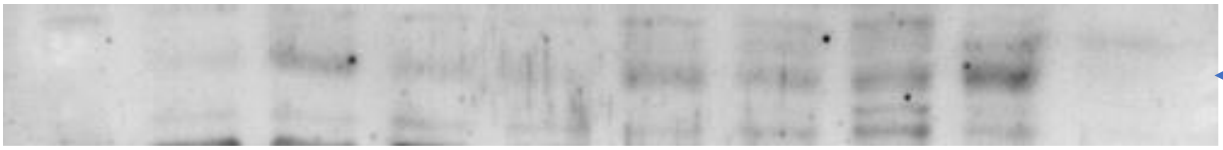

IB: active RHOA (21 kDa)

Long exposure
(no full membrane available)

Fig5D (bottom)

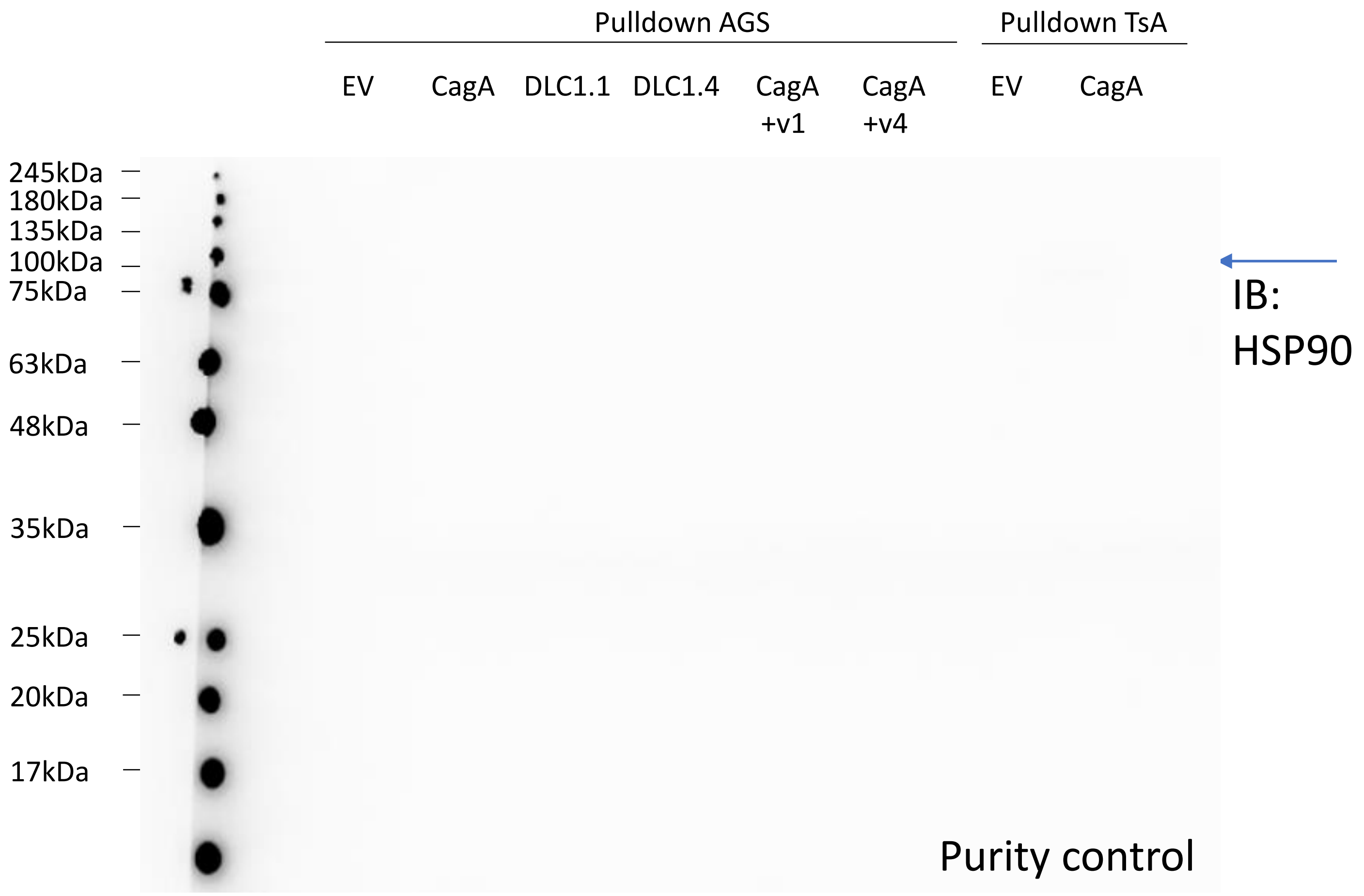

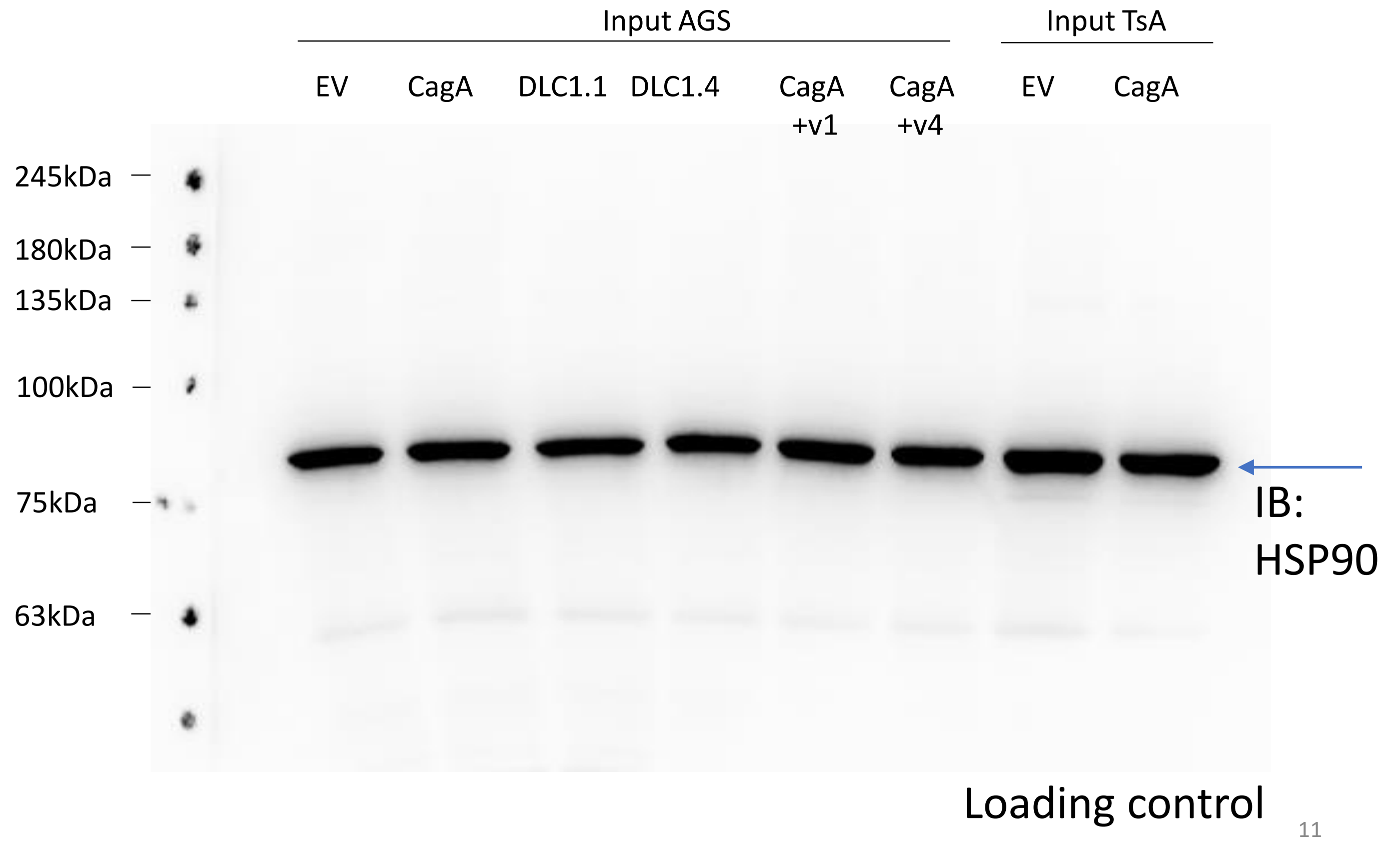

Fig5D (bottom)

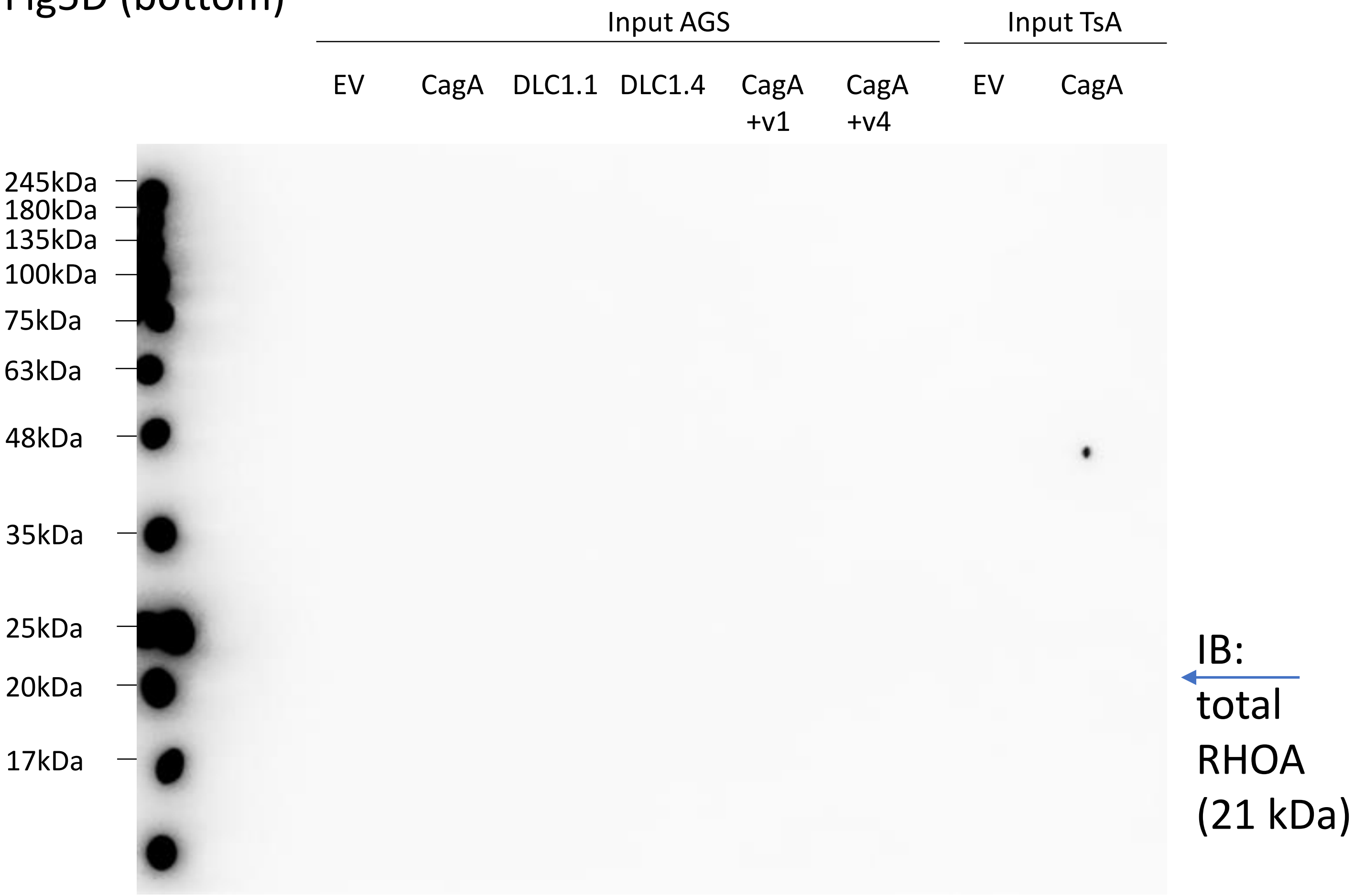

Short exposure

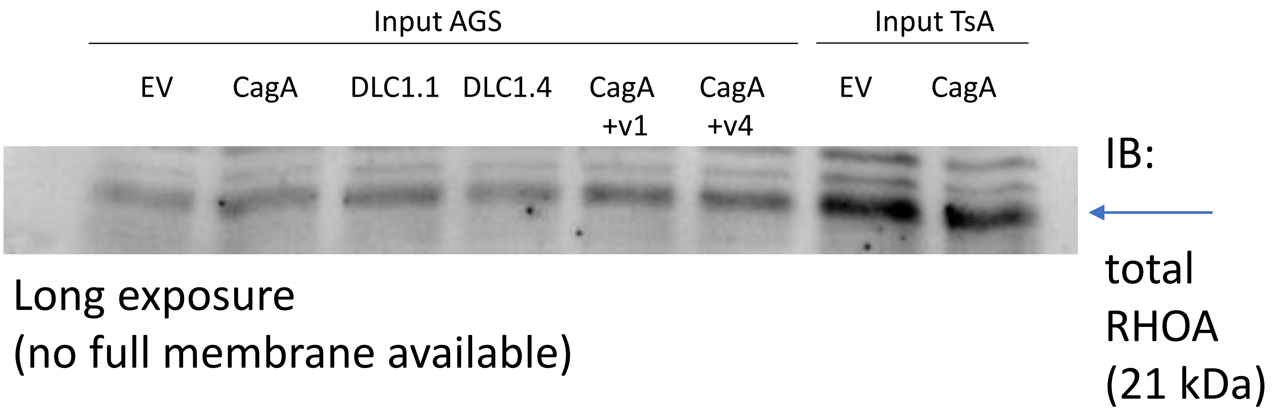

Long exposure
(no full membrane available)

Fig5D (bottom)

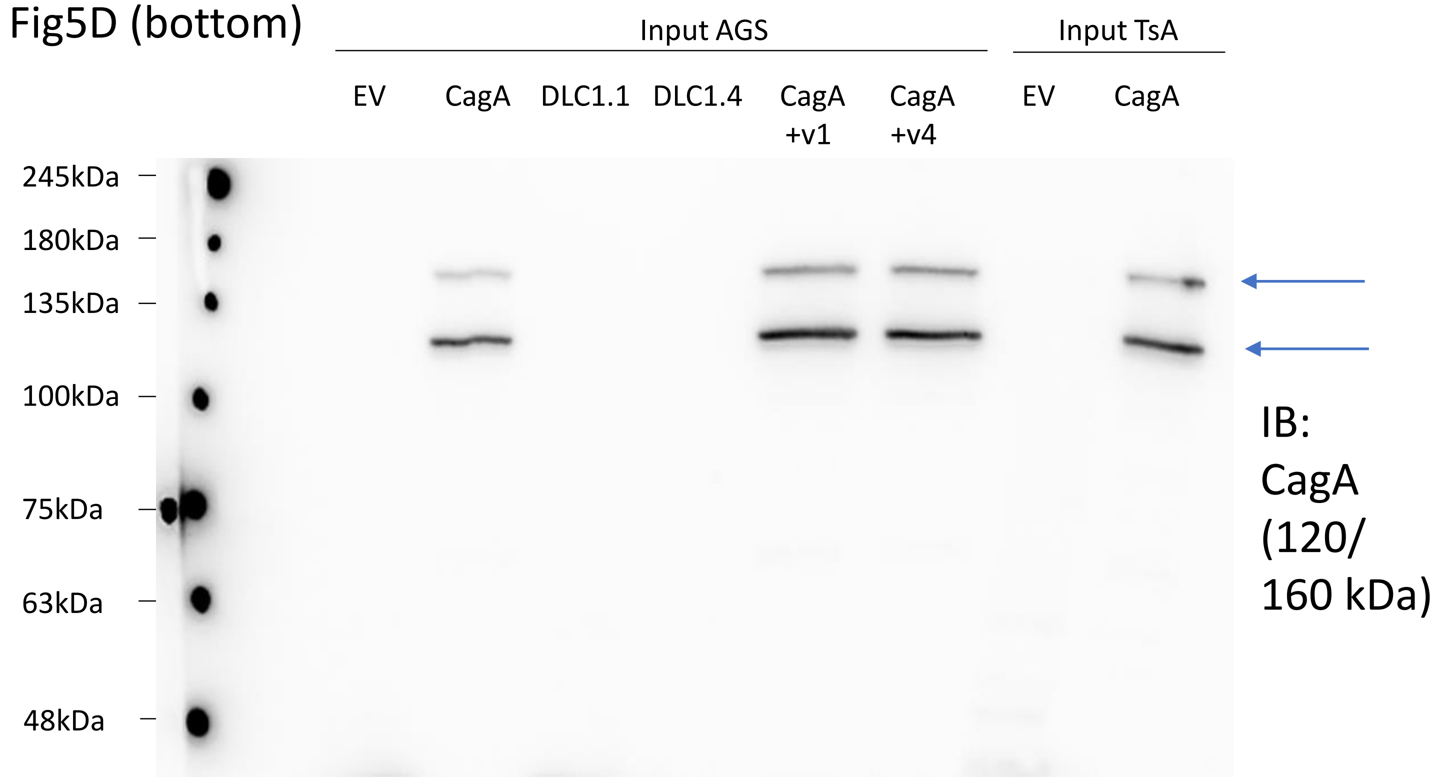

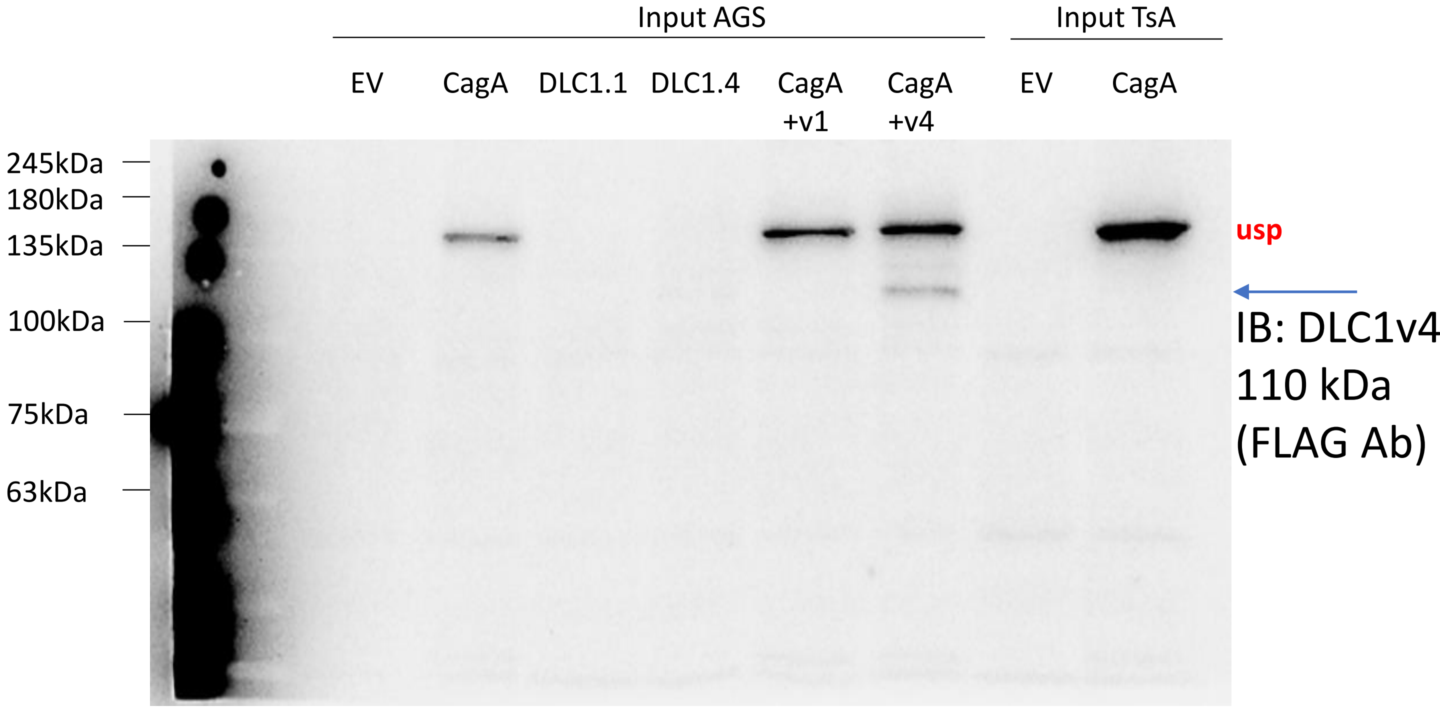

Short exposure (weak overexpression)

Fig5D (bottom)

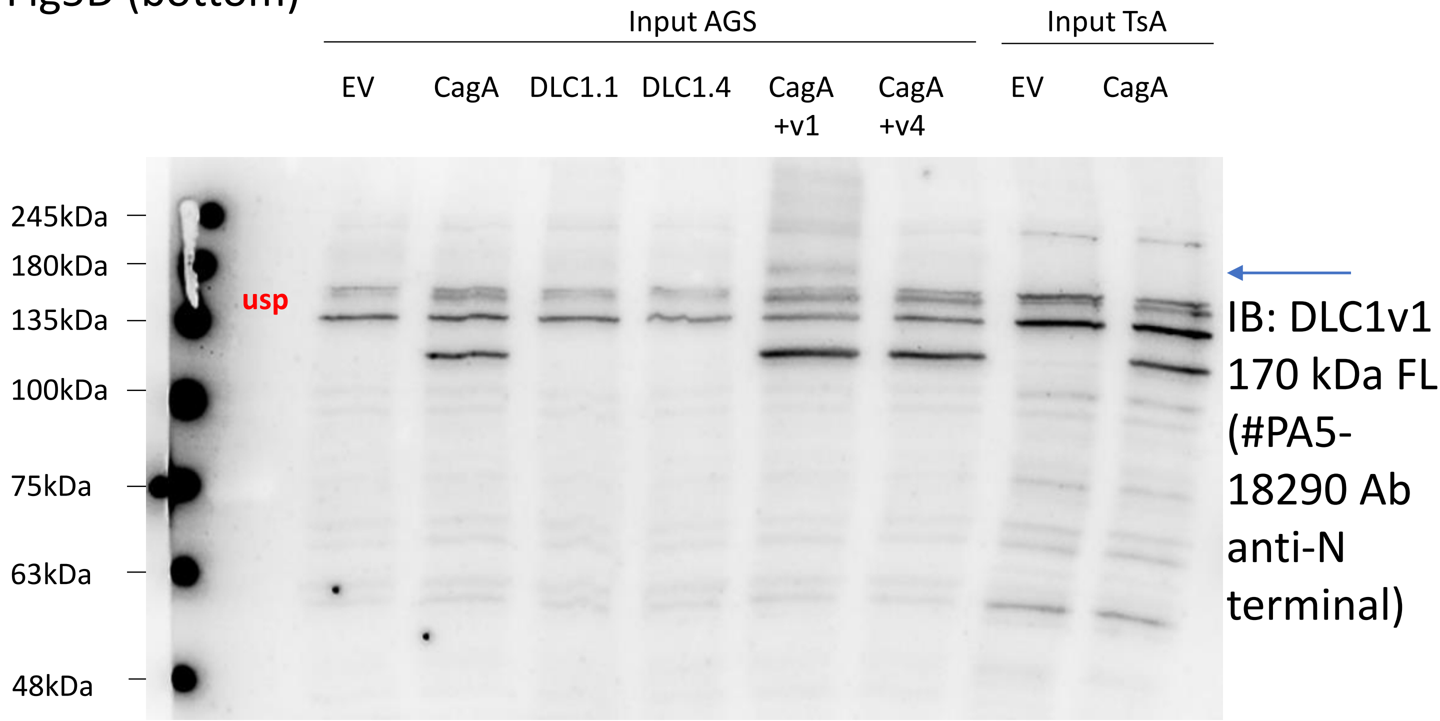

Short exposure (weak overexpression)

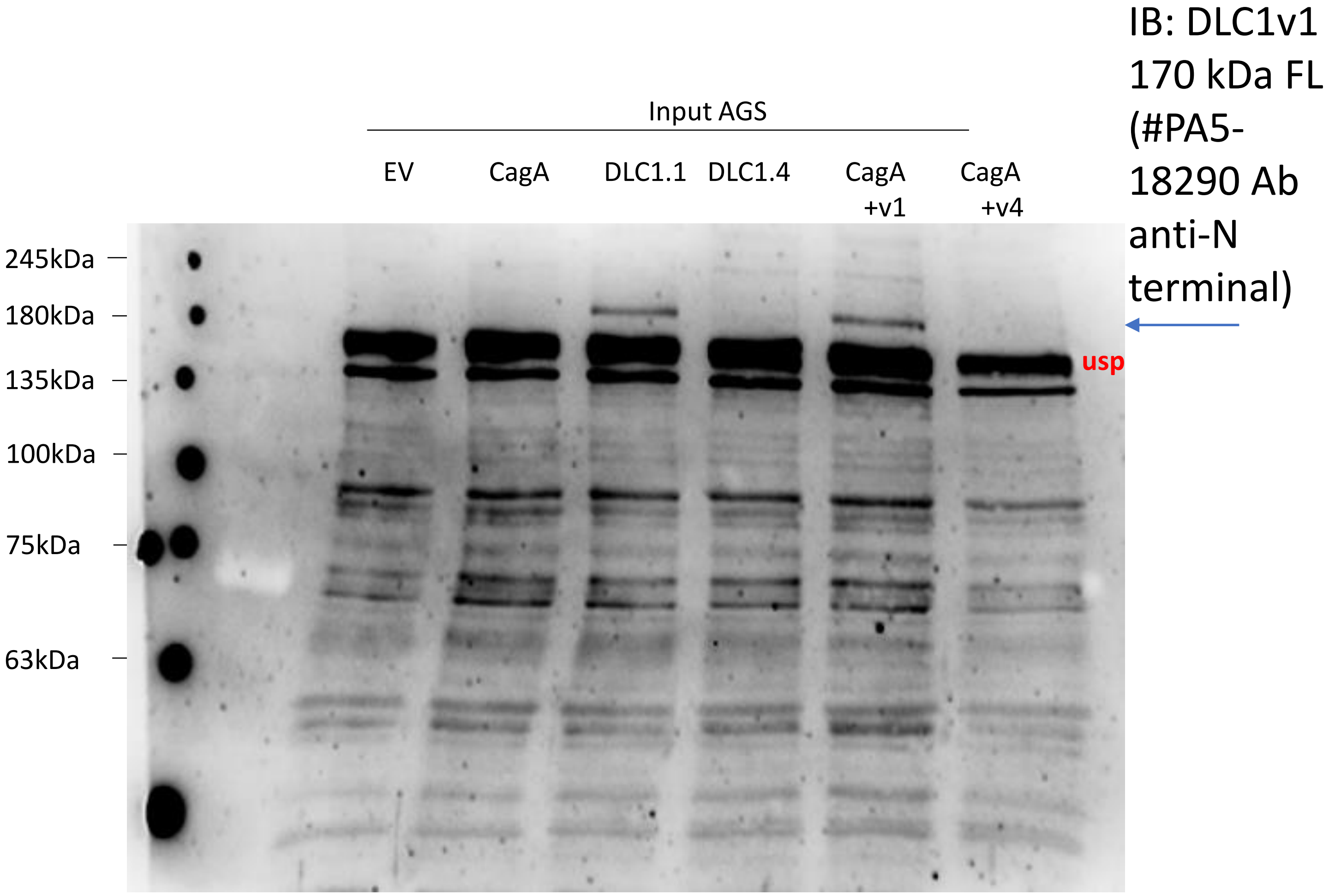

Long exposure

Fig6C

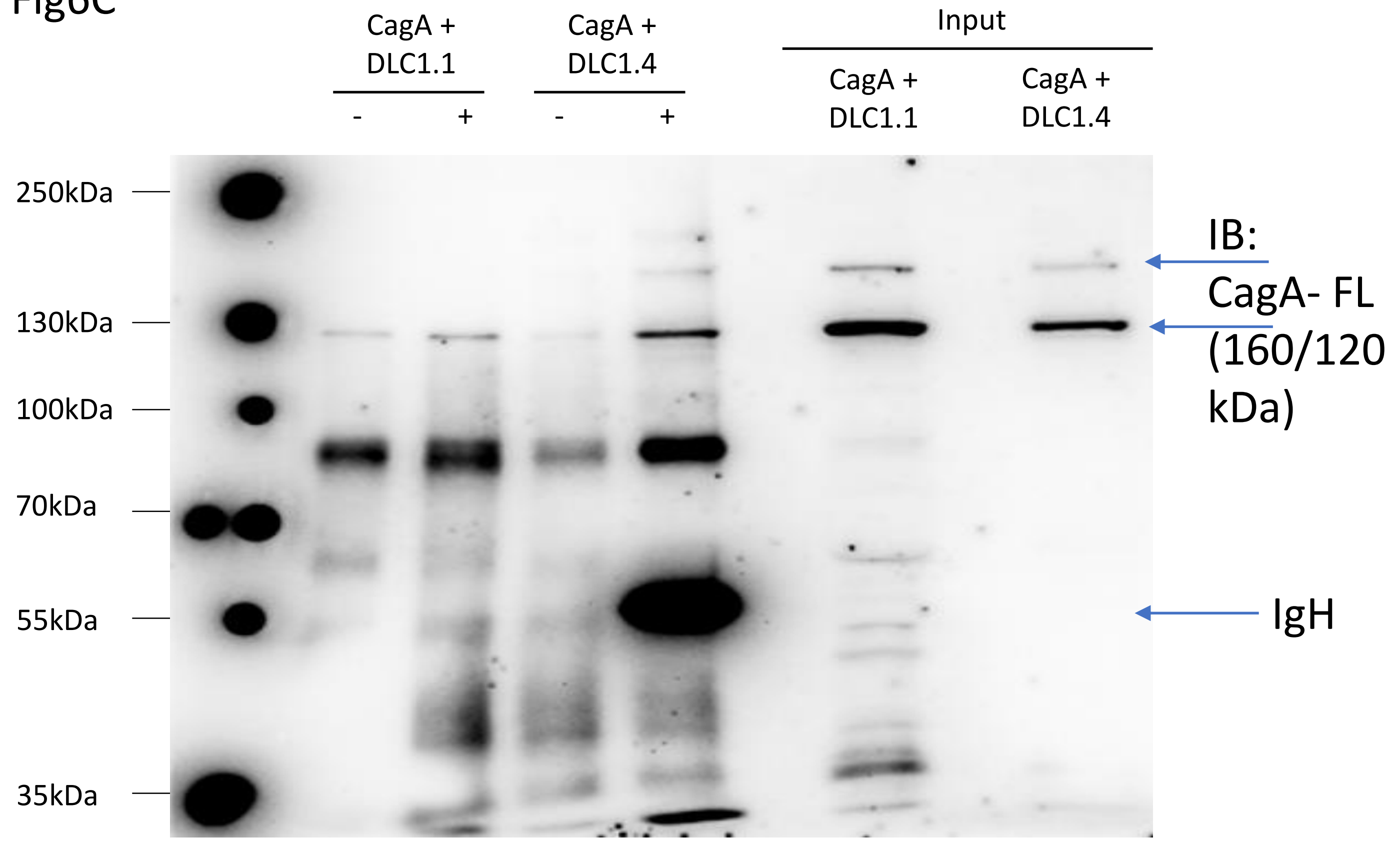
CagA + DLC1.1
CagA + DLC1.4
Input
CagA + DLC1.1
CagA + DLC1.4
- + - +
250kDa
130kDa
100kDa
70kDa
55kDa
35kDa
IB:
CagA- FL (160/120 kDa)
IgH

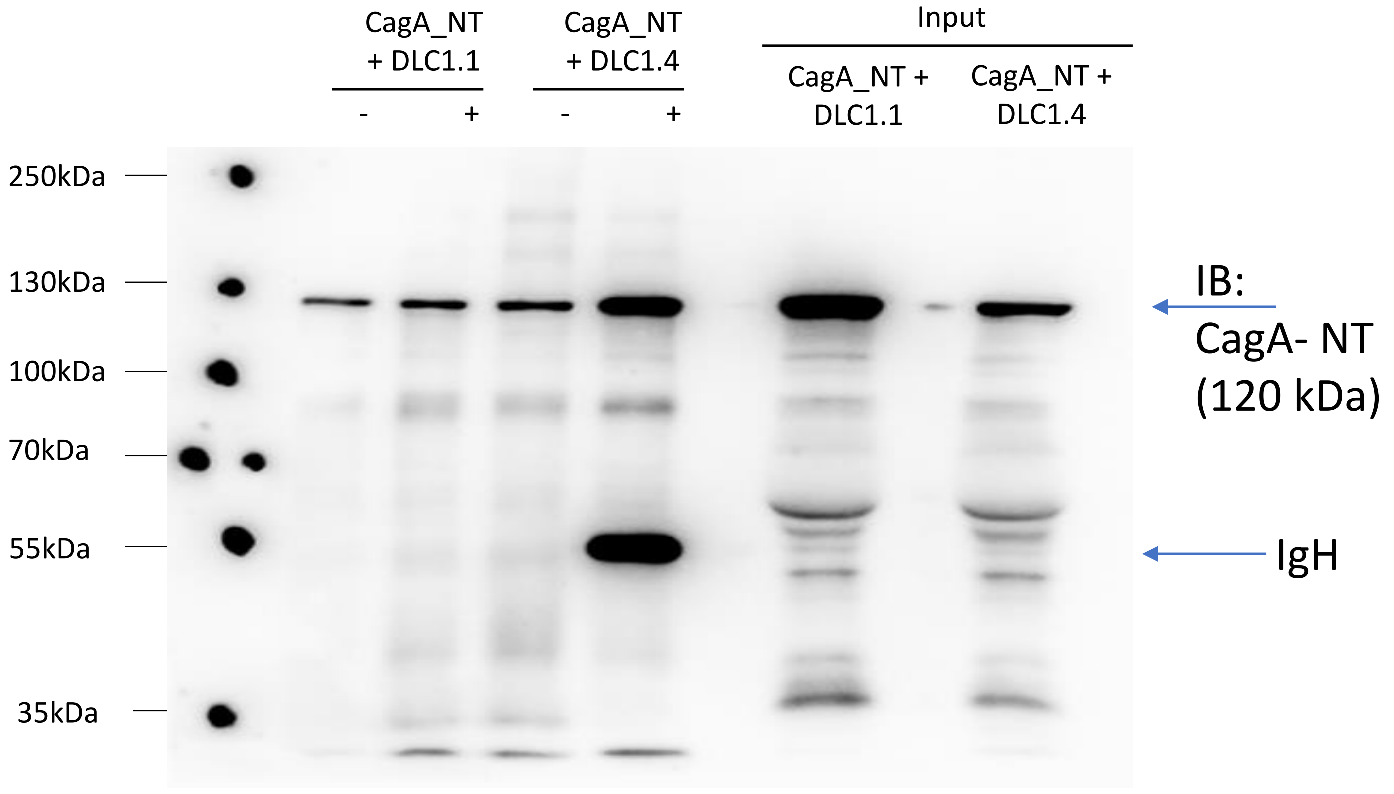
CagA_NT + DLC1.1
CagA_NT + DLC1.4
Input
CagA_NT + DLC1.1
CagA_NT + DLC1.4
- + - +
250kDa
130kDa
100kDa
70kDa
55kDa
35kDa
IB:
CagA- NT (120 kDa)
IgH

Fig6C

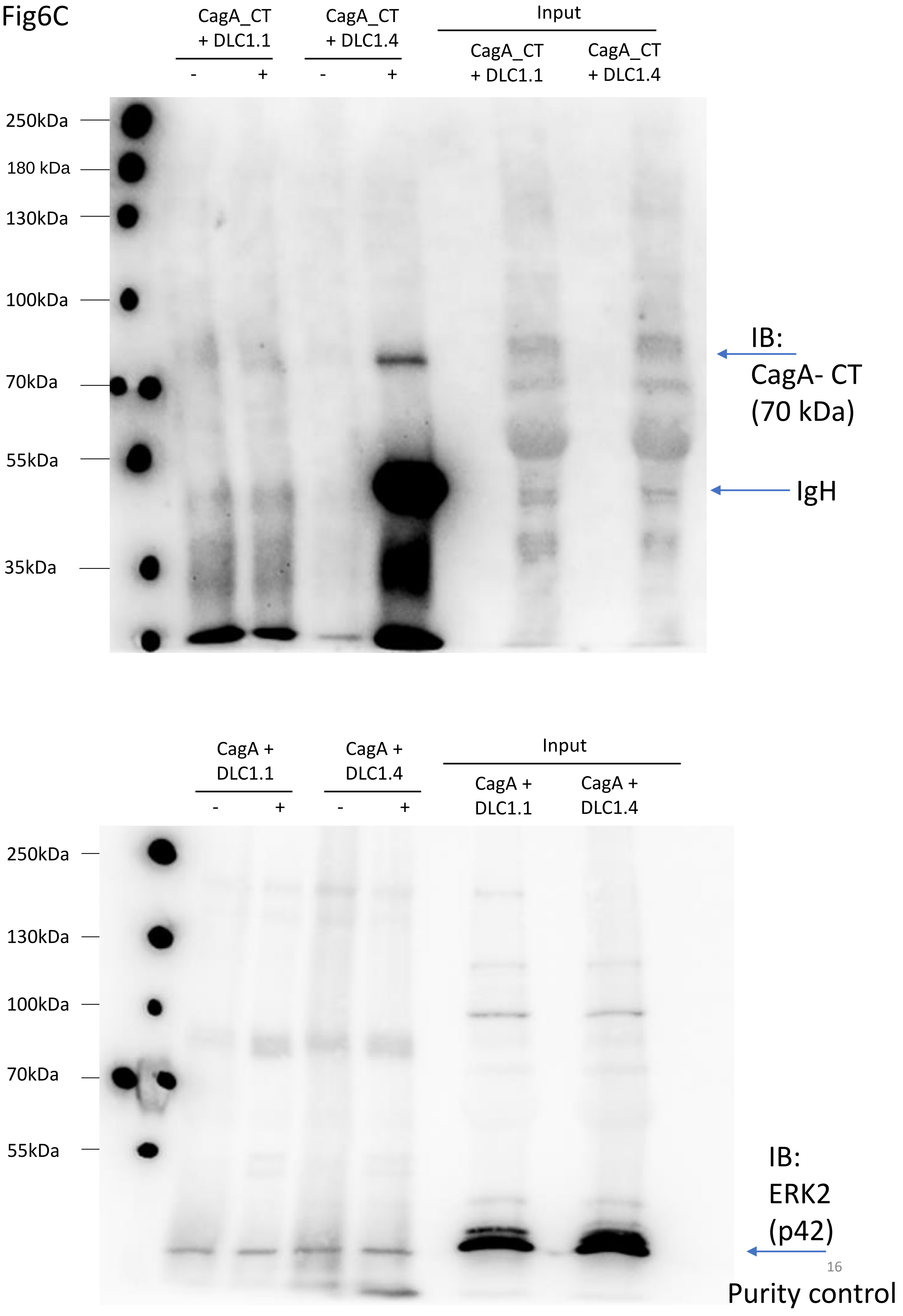
CagA_CT + DLC1.1
CagA_CT + DLC1.4
Input
- + - +
CagA_CT + DLC1.1
CagA_CT + DLC1.4
250kDa
180 kDa
130kDa
100kDa
70kDa
55kDa
35kDa
IB: CagA- CT (70 kDa)
IgH
CagA + DLC1.1
CagA + DLC1.4
Input
- + - +
CagA + DLC1.1
CagA + DLC1.4
250kDa
130kDa
100kDa
70kDa
55kDa
IB: ERK2 (p42)
Purity control

Fig6C

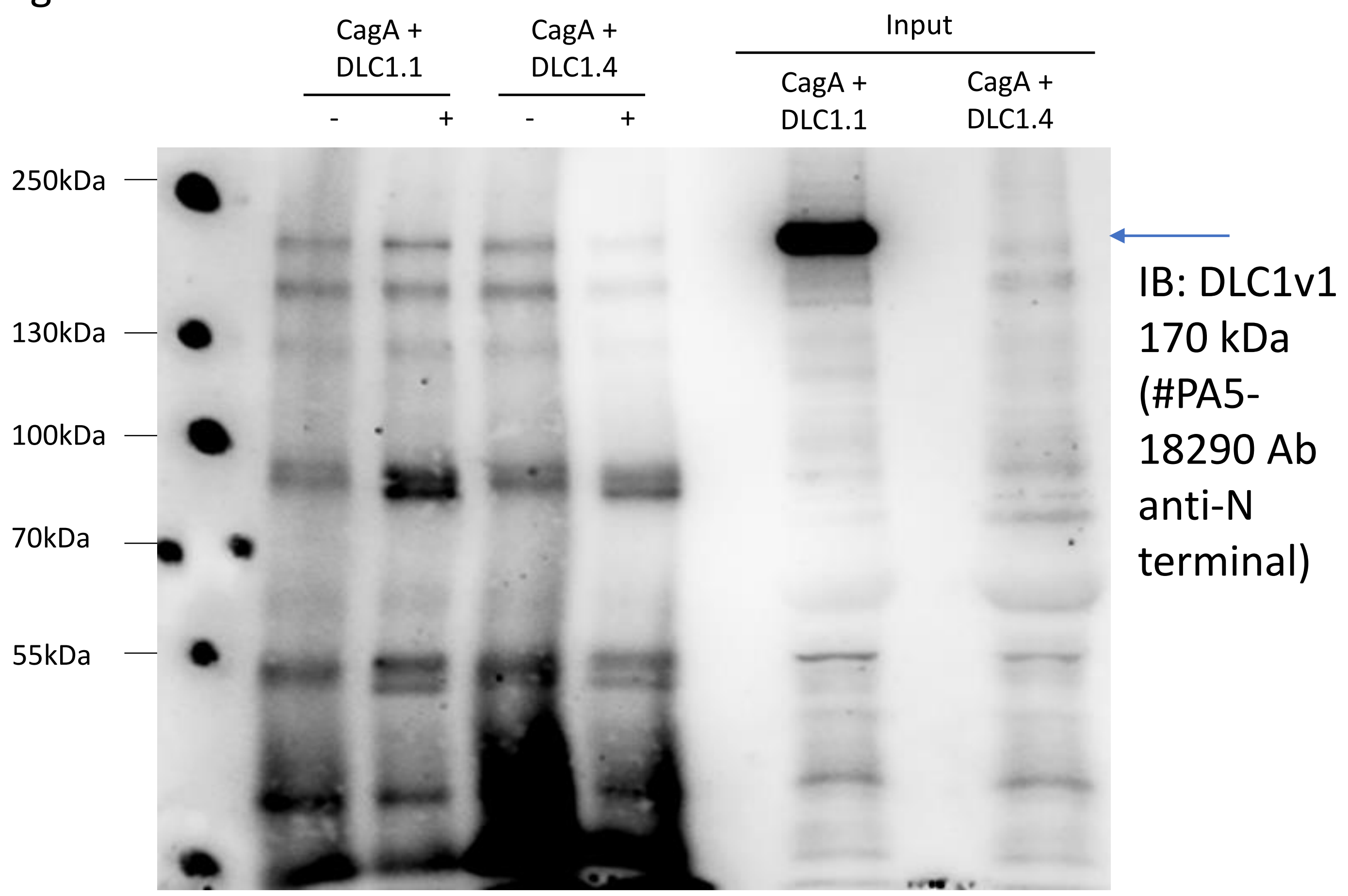
CagA + DLC1.1
CagA + DLC1.4
-
+
-
+
Input
CagA + DLC1.1
CagA + DLC1.4
250kDa
130kDa
100kDa
70kDa
55kDa
IB: DLC1v1
170 kDa
(#PA5-
18290 Ab
anti-N
terminal)

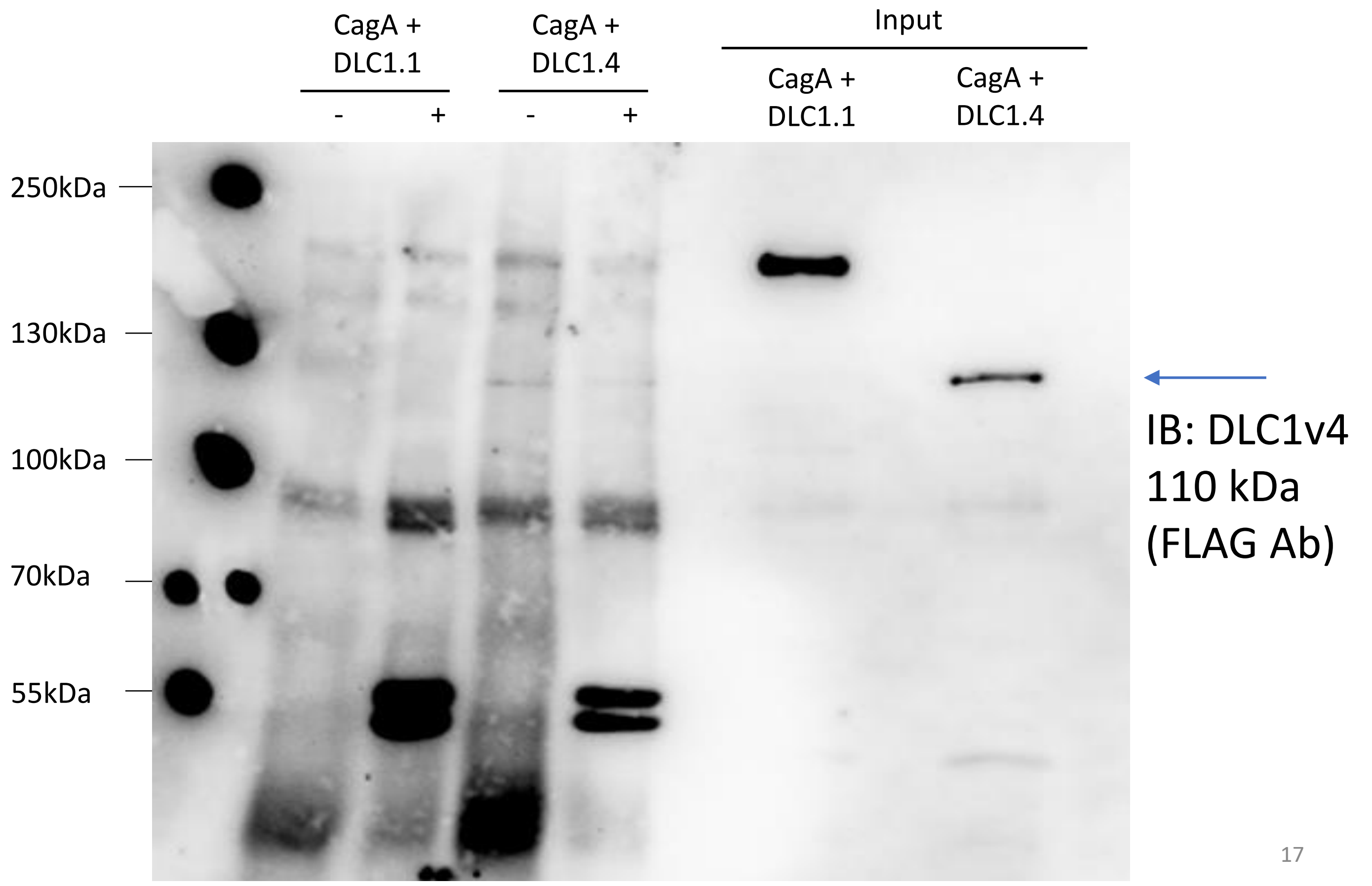
CagA + DLC1.1
CagA + DLC1.4
-
+
-
+
Input
CagA + DLC1.1
CagA + DLC1.4
250kDa
130kDa
100kDa
70kDa
55kDa
IB: DLC1v4
110 kDa
(FLAG Ab)

S9a

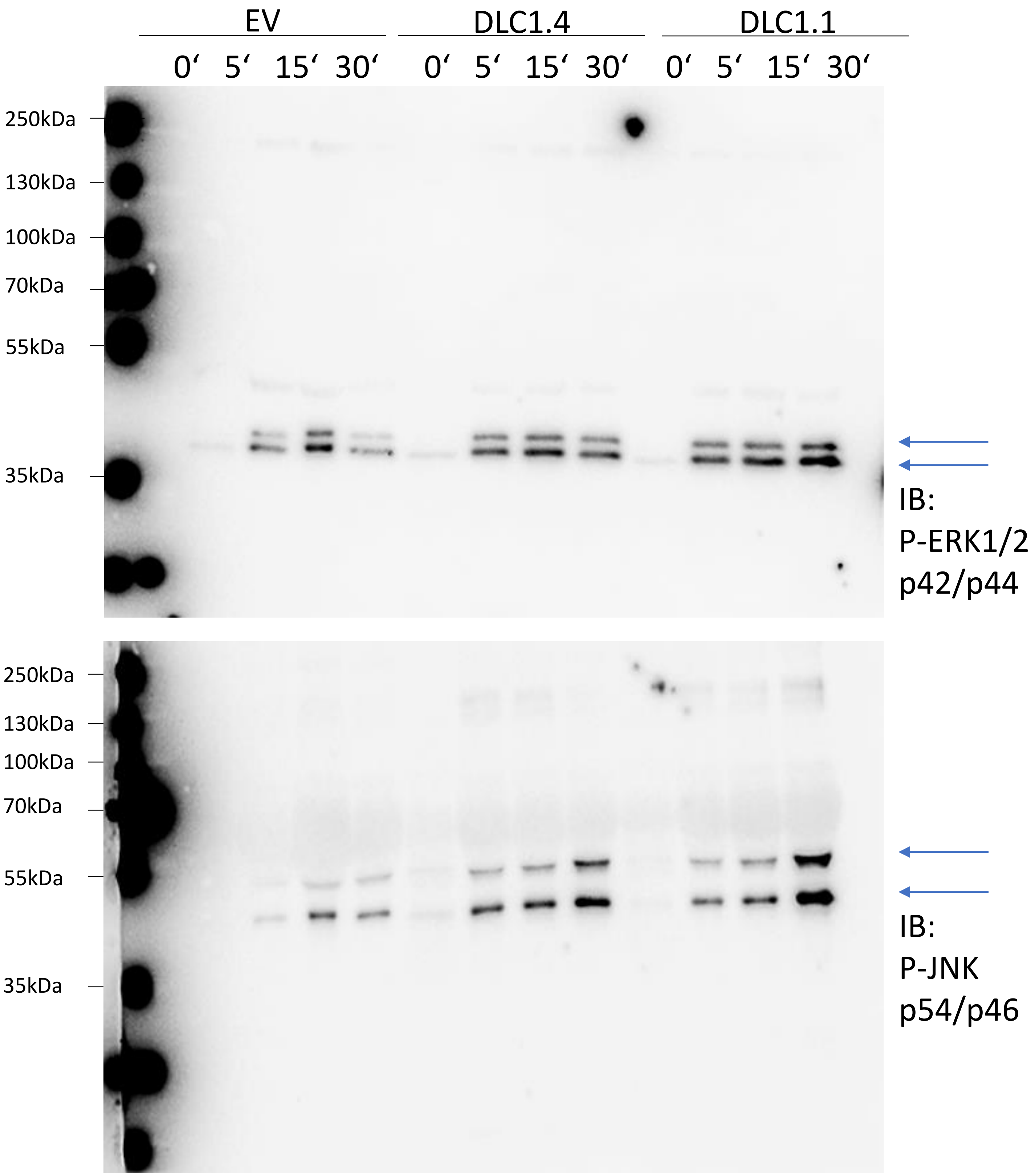

EV
DLC1.4
DLC1.1
0‘ 5‘ 15‘ 30‘ 0‘ 5‘ 15‘ 30‘ 0‘ 5‘ 15‘ 30‘
250kDa
130kDa
100kDa
70kDa
55kDa
35kDa
IB:
P-ERK1/2
p42/p44
250kDa
130kDa
100kDa
70kDa
55kDa
35kDa
IB:
P-JNK
p54/p46

S9a

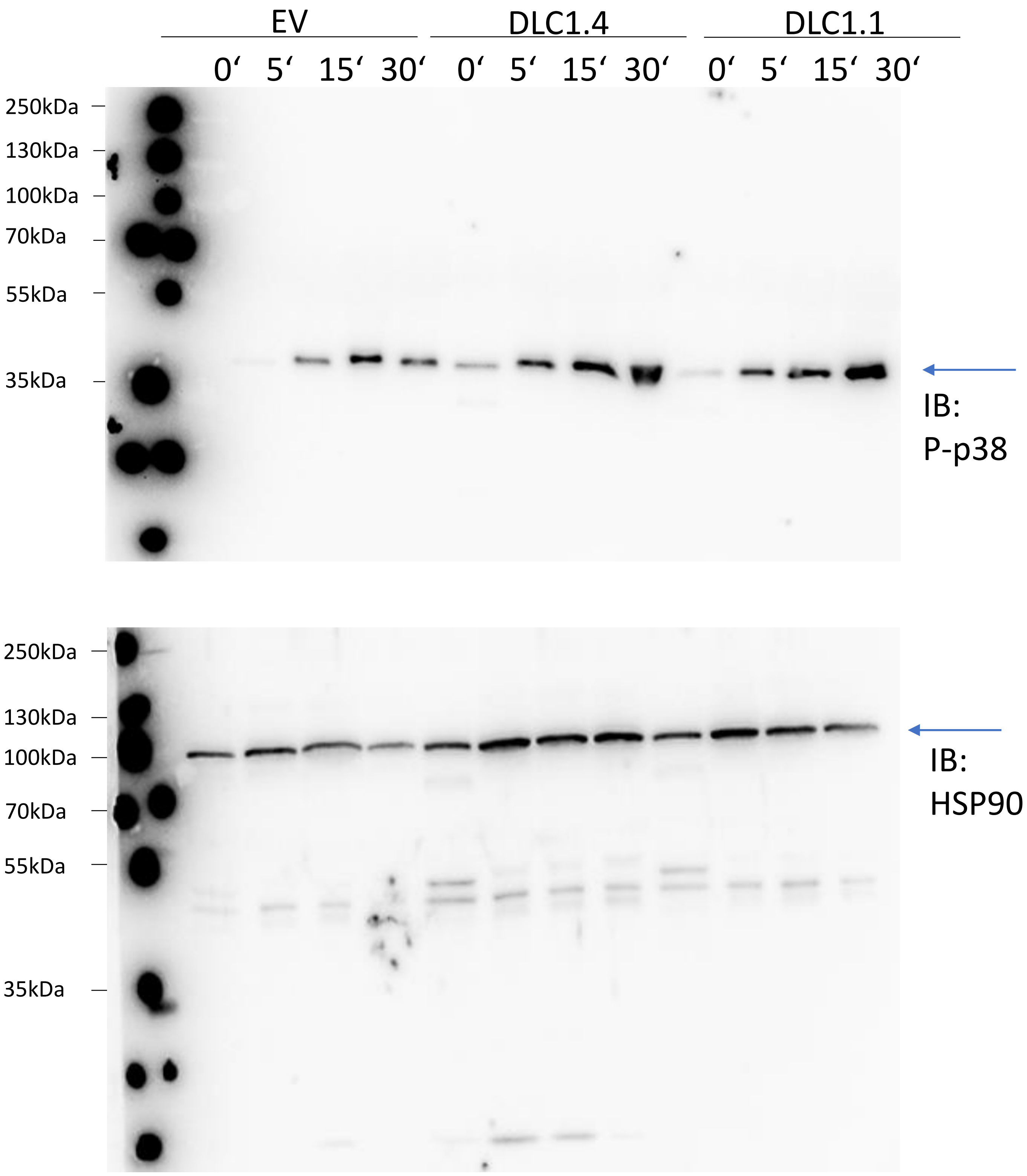
EV
DLC1.4
DLC1.1
0‘ 5‘ 15‘ 30‘ 0‘ 5‘ 15‘ 30‘ 0‘ 5‘ 15‘ 30‘
250kDa
130kDa
100kDa
70kDa
55kDa
35kDa
IB: P-p38
250kDa
130kDa
100kDa
70kDa
55kDa
35kDa
IB: HSP90

S9c(left)

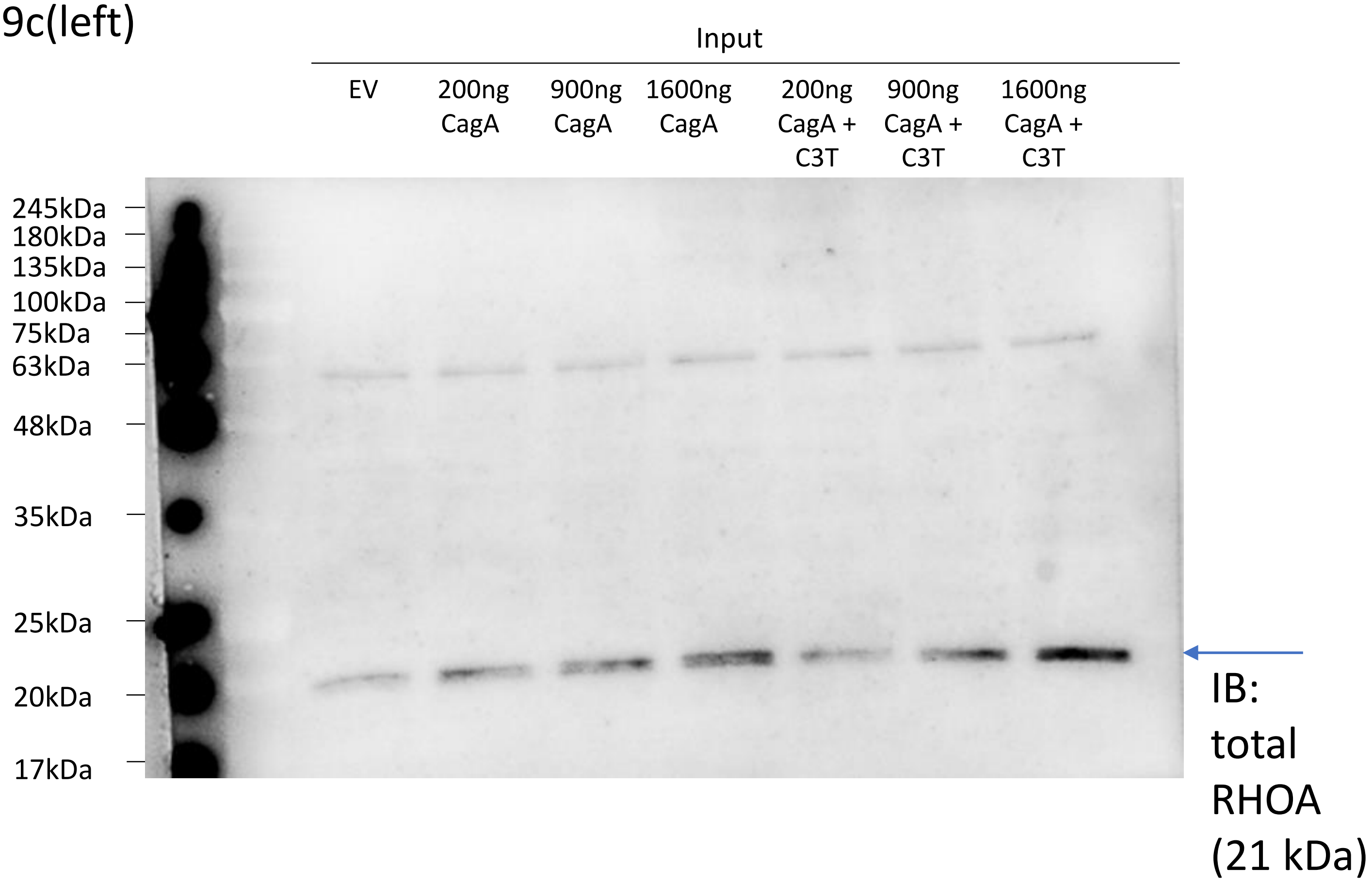

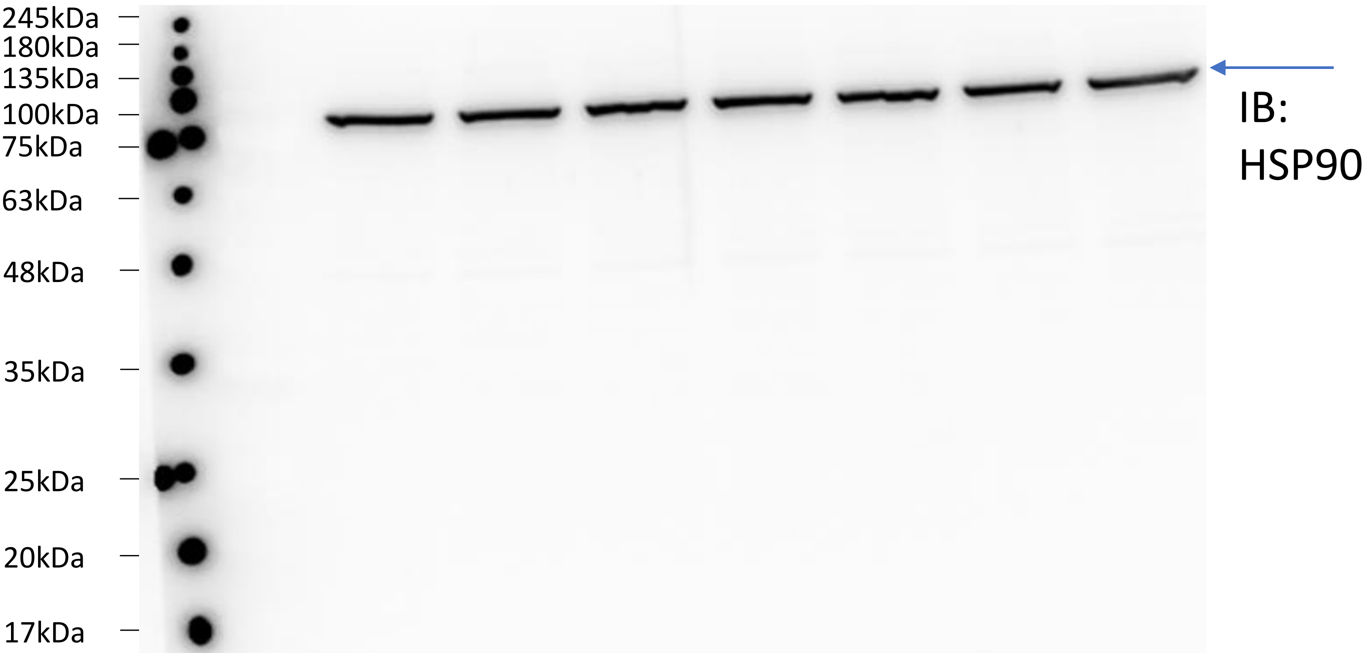

Loading control

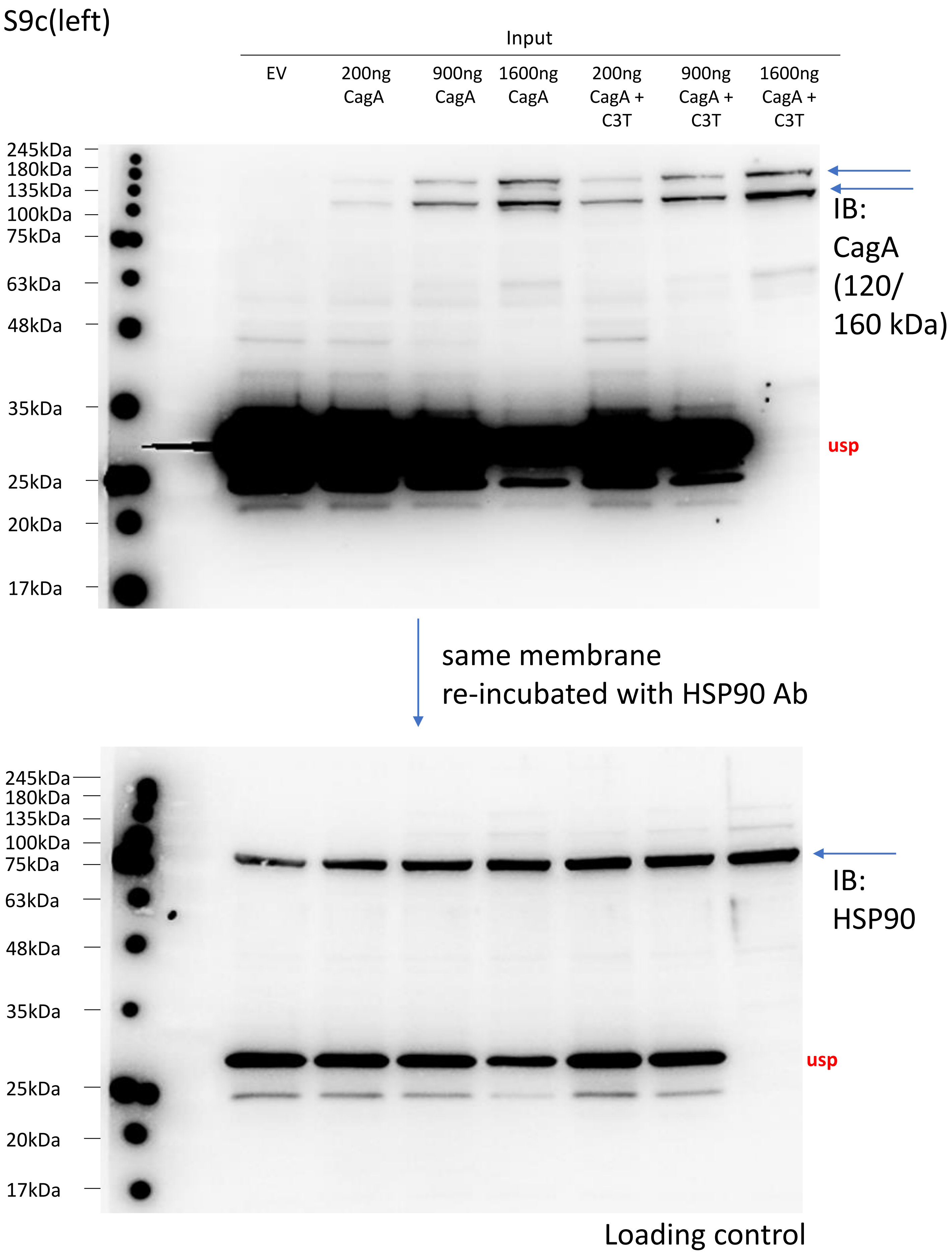
S9c(left)
Input
EV
200ng CagA
900ng CagA
1600ng CagA
200ng CagA + C3T
900ng CagA + C3T
1600ng CagA + C3T
245kDa
180kDa
135kDa
100kDa
75kDa
63kDa
48kDa
35kDa
25kDa
20kDa
17kDa
IB: CagA (120/ 160 kDa)
usp
same membrane
re-incubated with HSP90 Ab
245kDa
180kDa
135kDa
100kDa
75kDa
63kDa
48kDa
35kDa
25kDa
20kDa
17kDa
IB: HSP90
usp
Loading control

S9c(left)

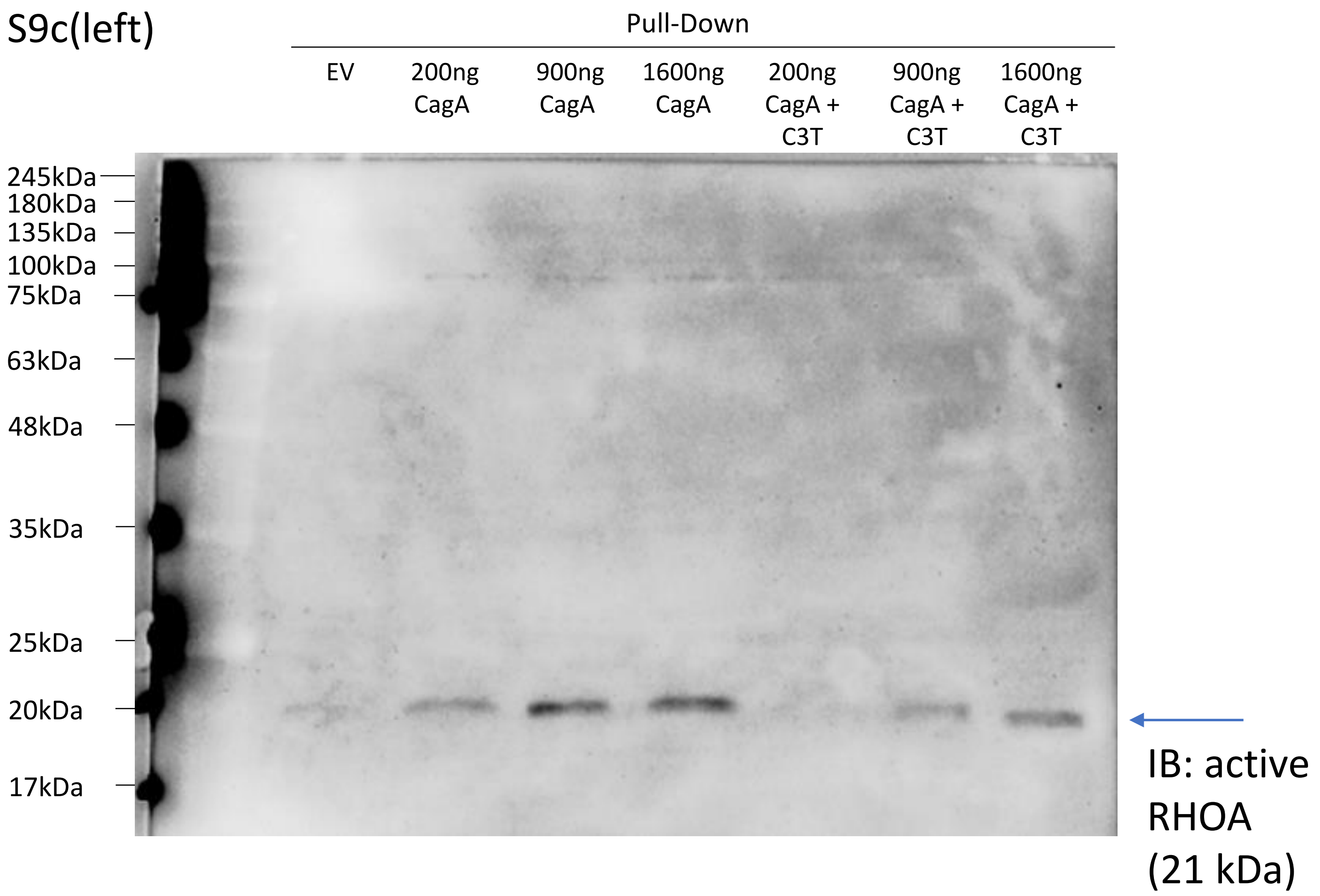

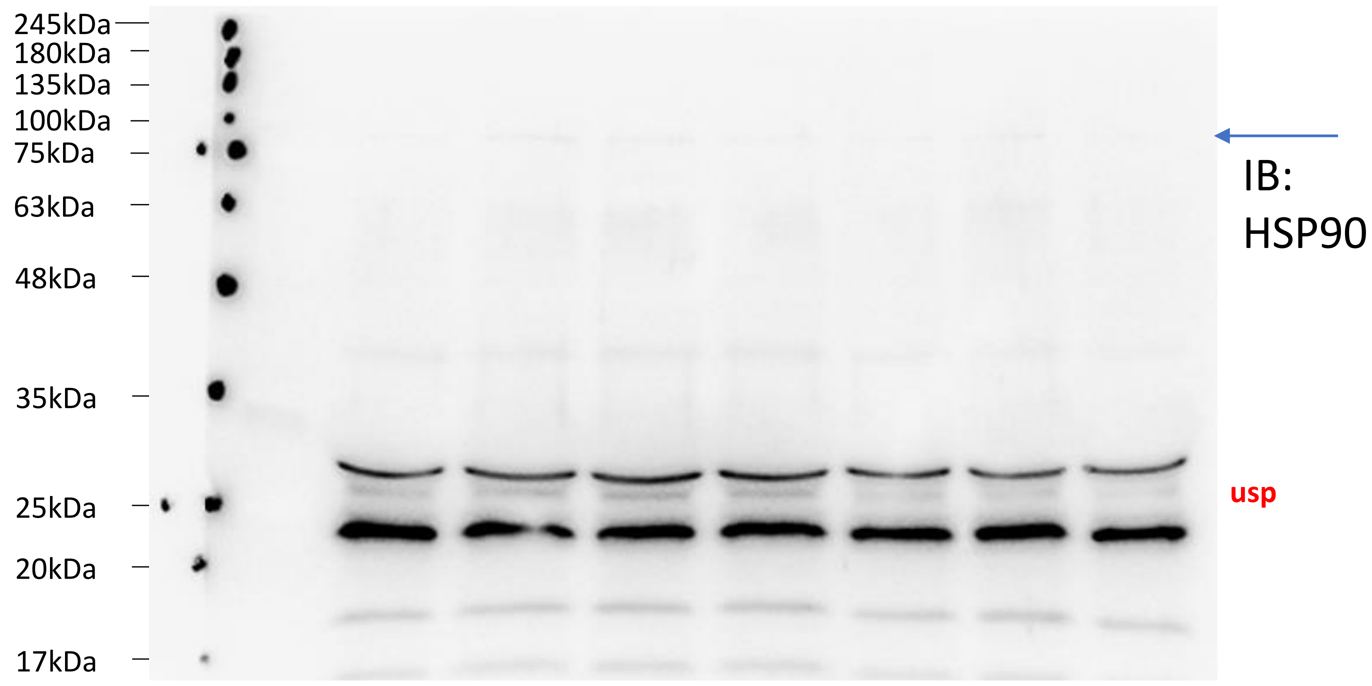

purity control

S9c(right)

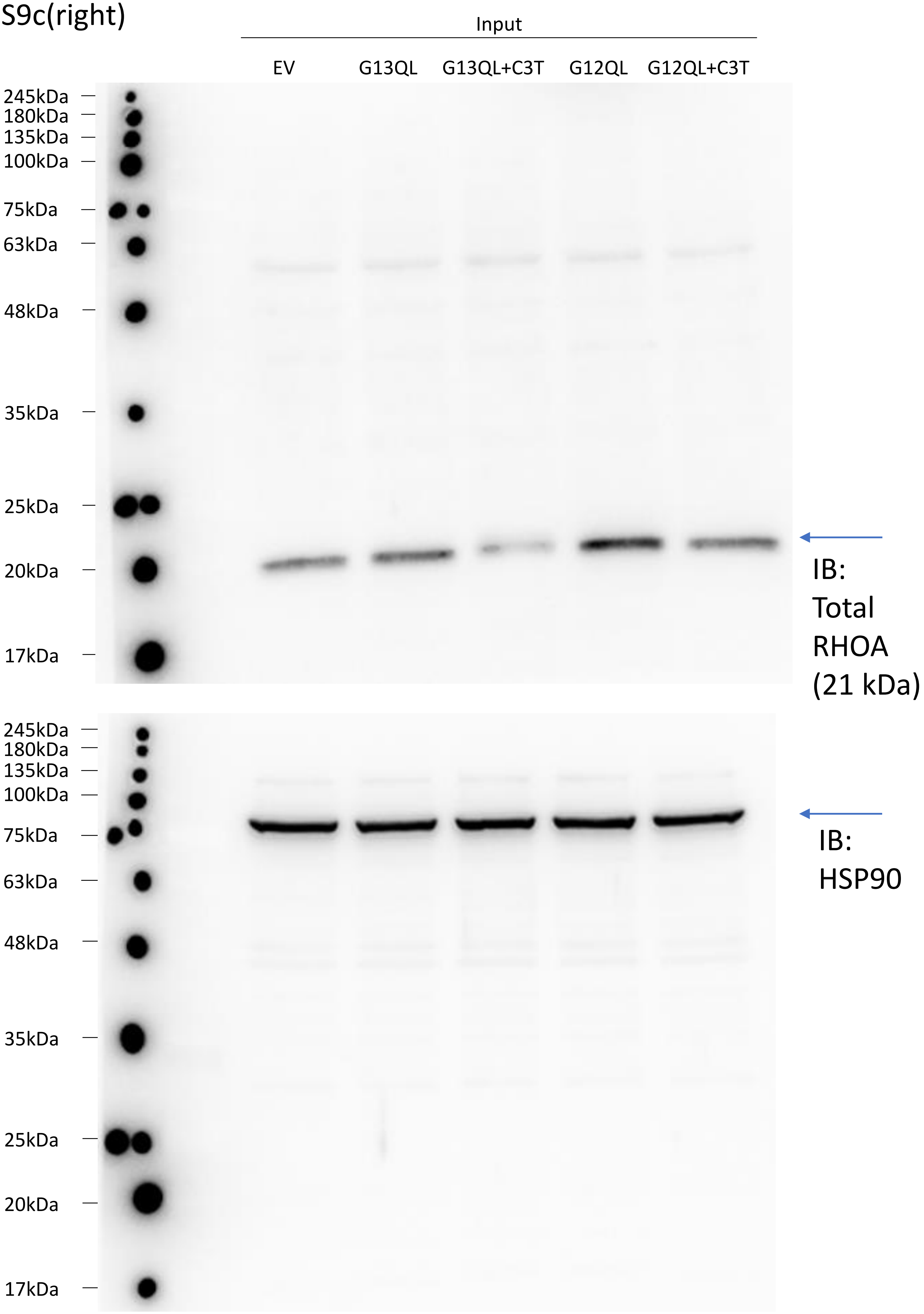

Loading control

S9c(right)

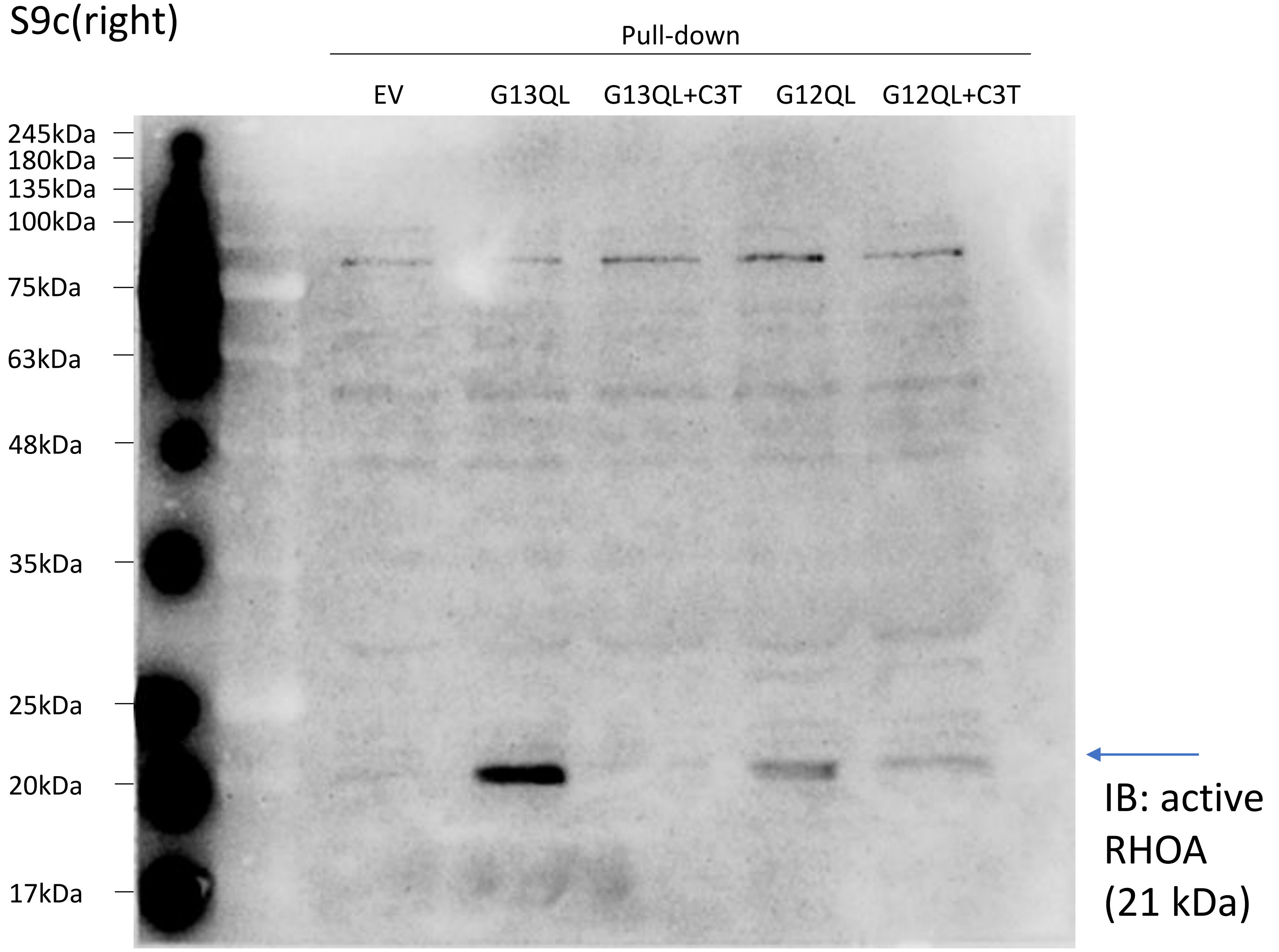

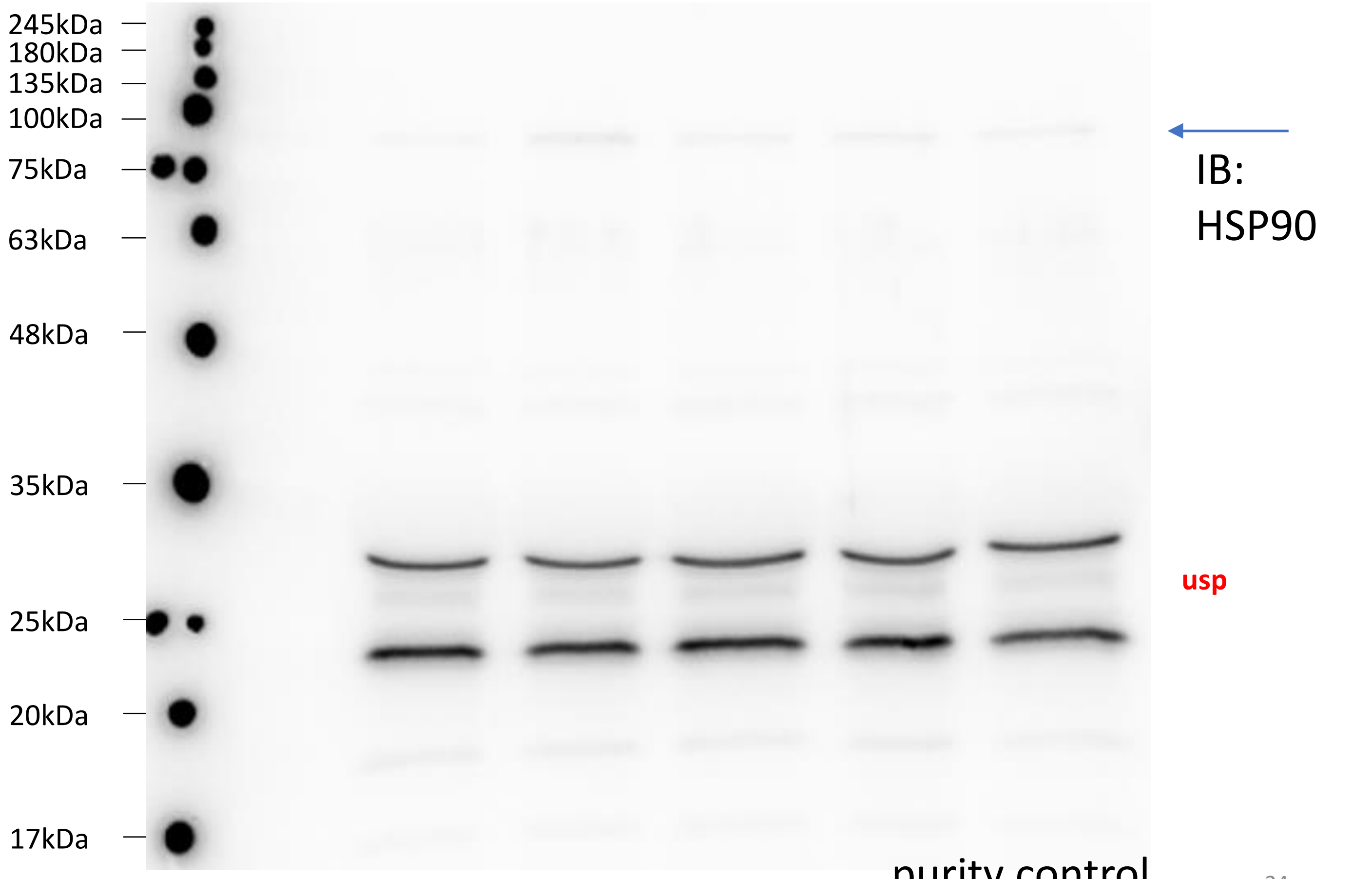

purity control

S10

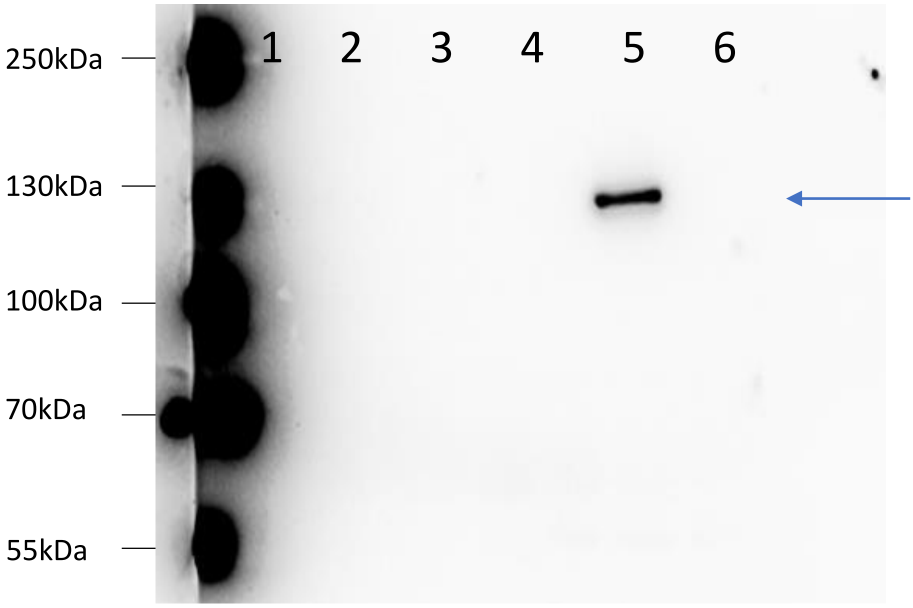

IB: anti-FLAG Ab
DLC1v4
110 kDa

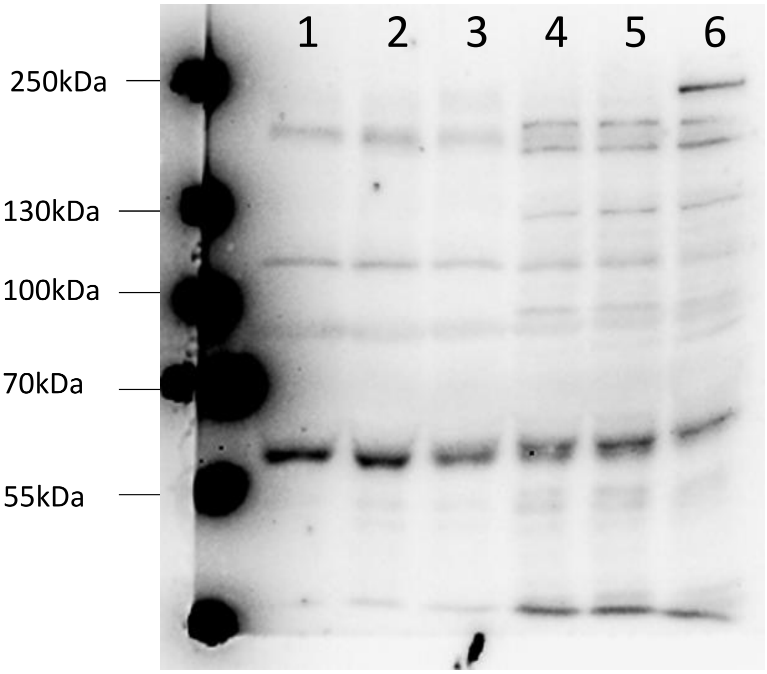

IB:
DLC1v1 FL
170 kDa
(#PA5-18290 Ab
anti-N terminal)

AGS:
1=EV
2=DLC1v4
3=DLC1v1
HEK293T:
4=EV
5=DLC1v4
6=DLC1v1

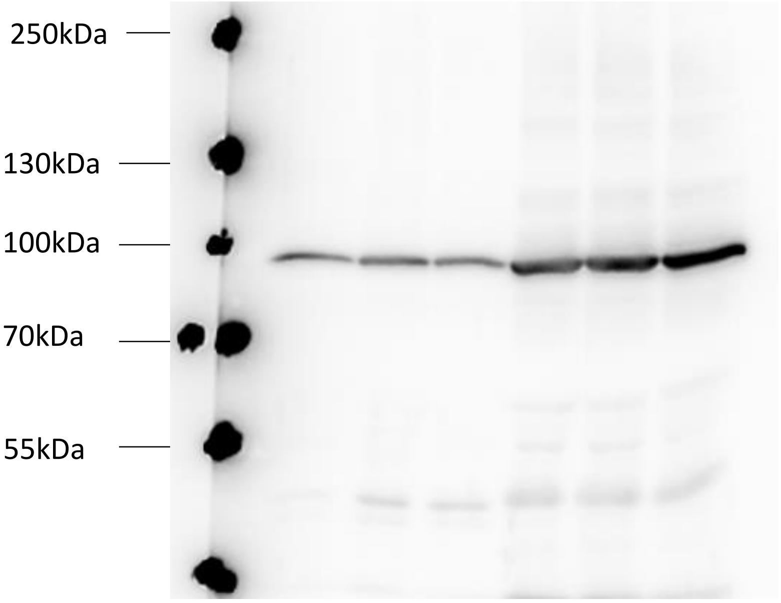

IB:
HSP90

S11a

Liver tissue

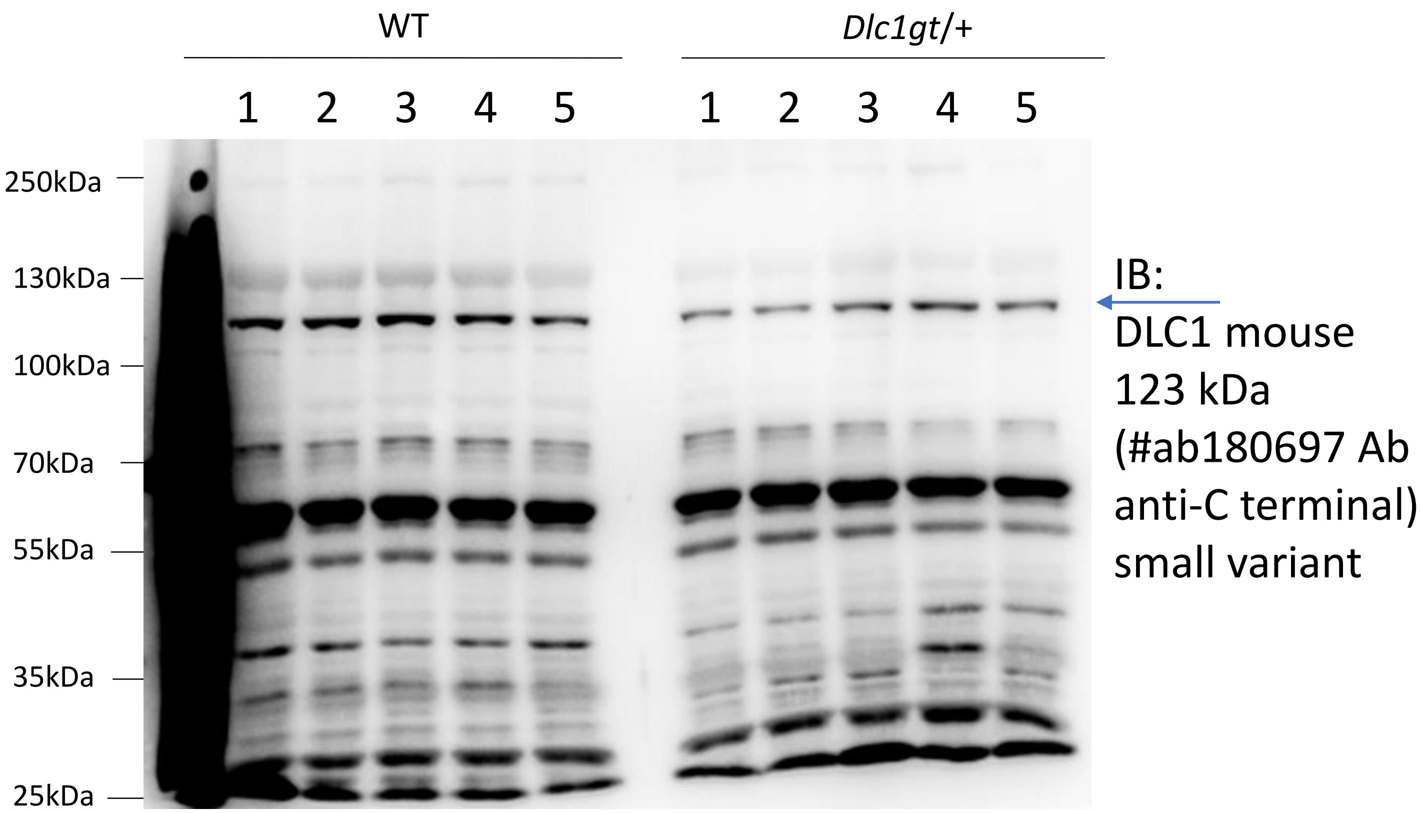

n=5 mice
per genotype

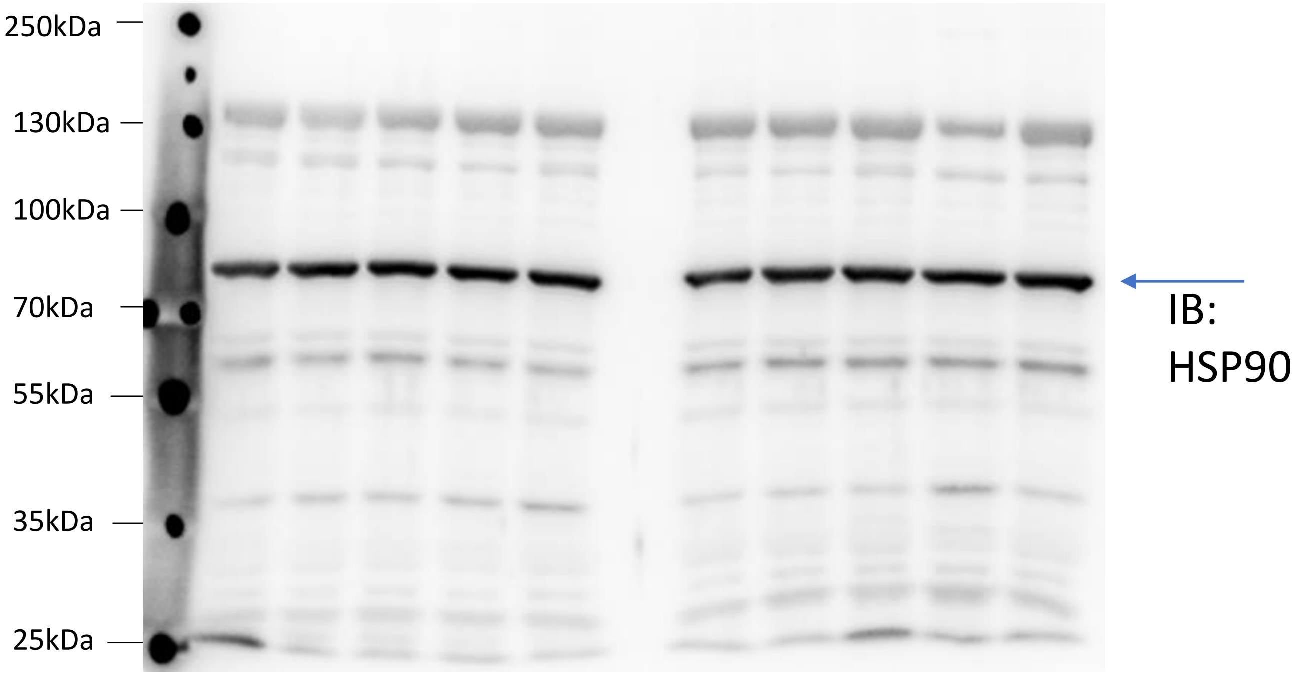

S14b

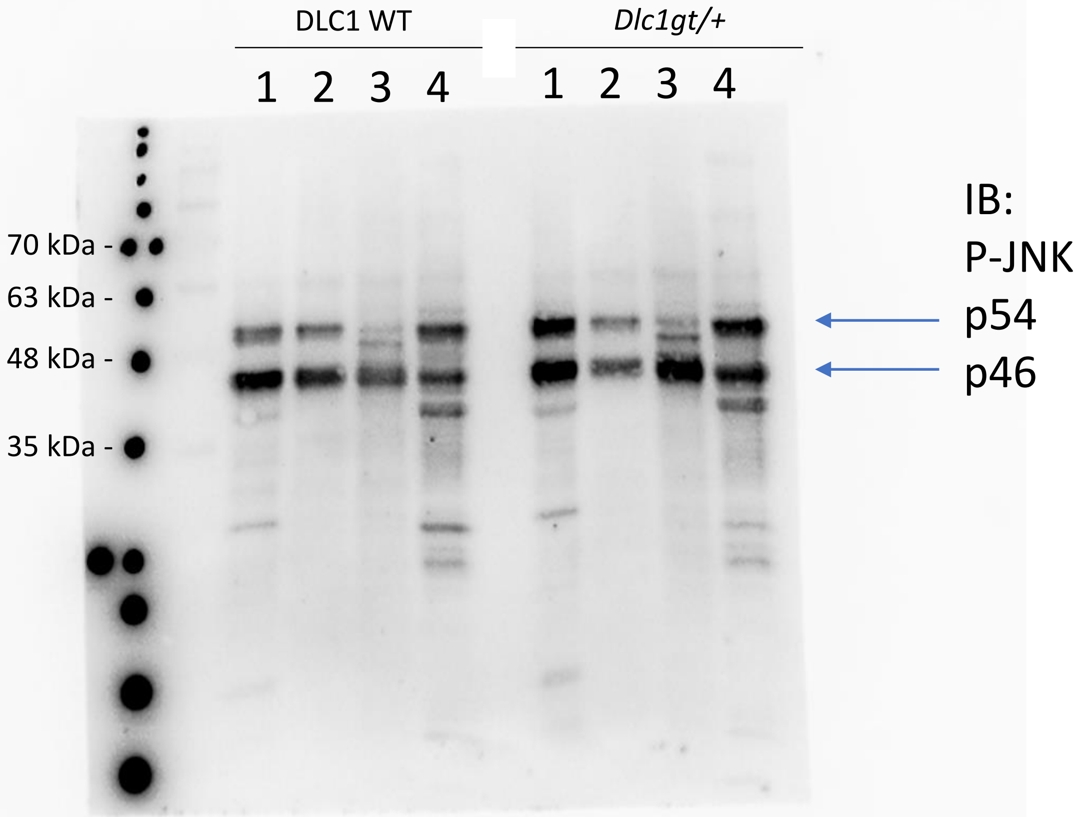

1=Corpus
2=Forestomach
3=Small Intestine
4=Liver

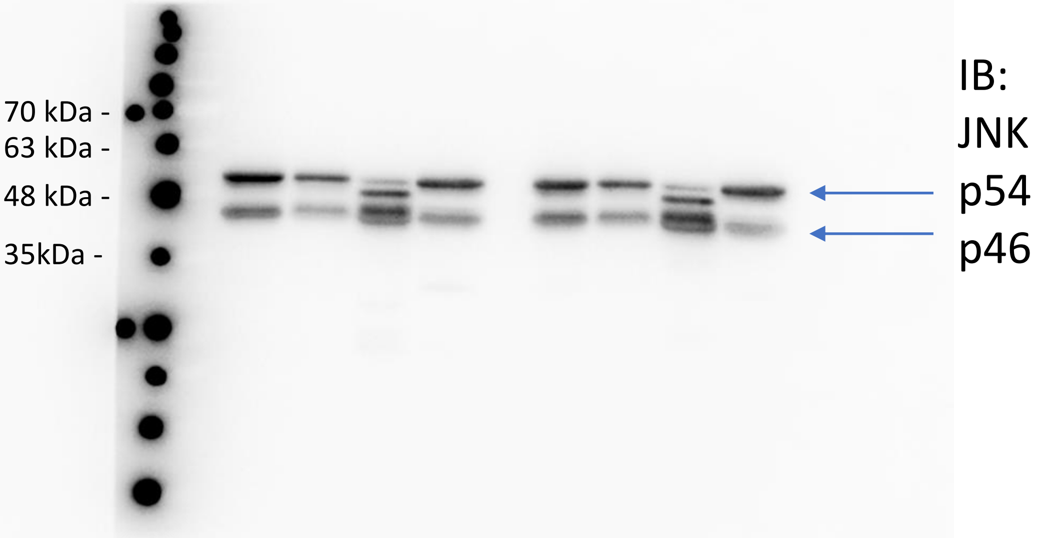

S14b

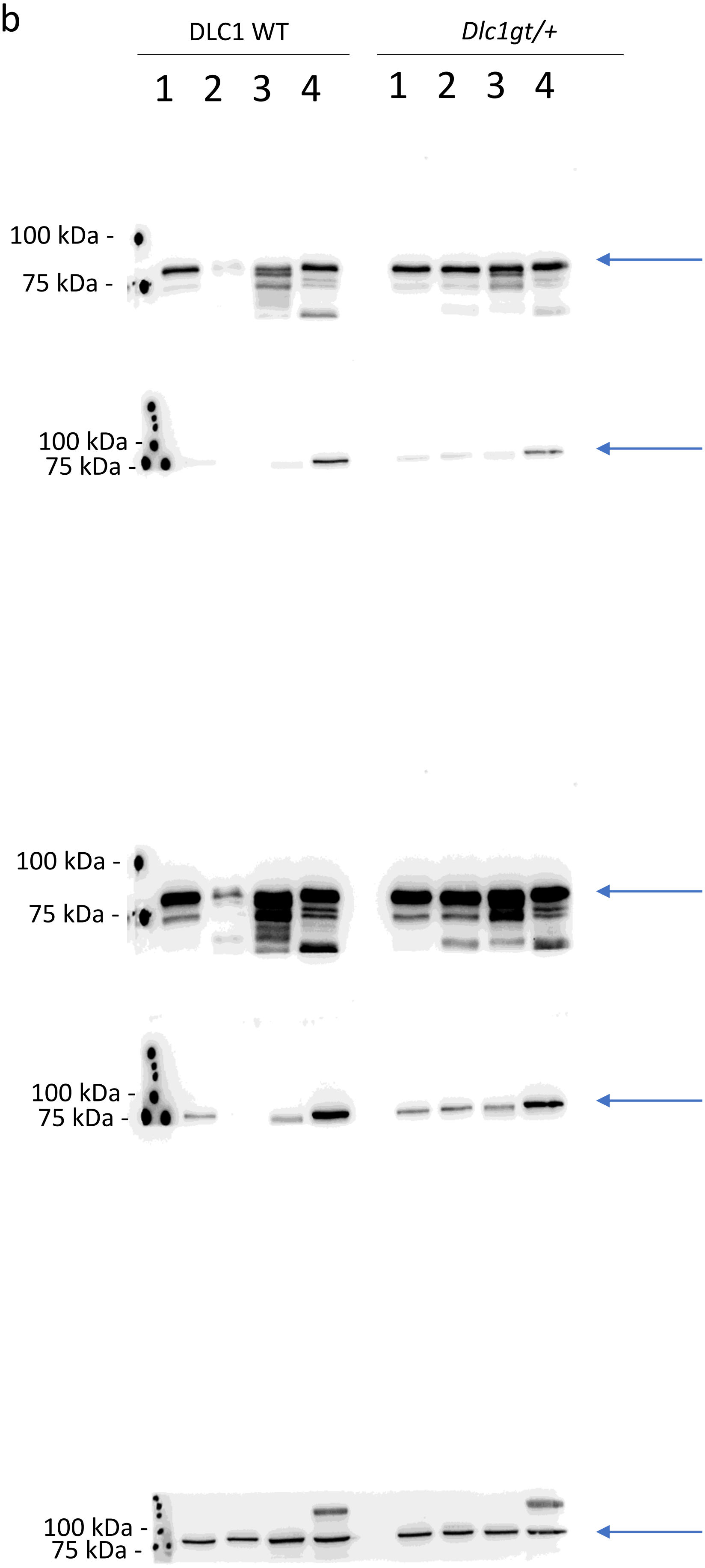

Short exposure
IB:
STAT3
(p79/p86)

IB:
P-STAT3
(p79/p86)

Long exposure
IB:
STAT3
(p79/p86)

IB:
P-STAT3
(p79/p86)

1=Corpus (stomach)
2=Forestomach
3=Small Intestine
4=Liver

IB:
HSP90
(no full membrane available)
